# Author Correction: The vacuolar fusion regulated by HOPS complex promotes hyphal initiation and penetration in *Candida albicans*

**DOI:** 10.1038/s41467-024-49022-5

**Published:** 2024-05-29

**Authors:** Yu Liu, Ruina Wang, Jiacun Liu, Mengting Fan, Zi Ye, Yumeng Hao, Fei Xie, Ting Wang, Yuanying Jiang, Ningning Liu, Xiaoyan Cui, Quanzhen Lv, Lan Yan

**Affiliations:** 1 Center for Basic Research and Innovation of Medicine and Pharmacy (MOE), School of Pharmacy, Naval Medical University, Shanghai, 200433 PR China; 2 School of Chemistry and Molecular Engineering, East China Normal University, Shanghai, 200241 PR China; 3 School of Medicine, Tongji University, Shanghai, 200092 PR China; 4 State Key Laboratory of Systems Medicine for Cancer, Center for Single-Cell Omics, School of Public Health, Shanghai Jiao Tong University School of Medicine, Shanghai, 200025 PR China

**Keywords:** Fungal pathogenesis, Infection, Fungal infection

Correction to: *Nature Communications* 10.1038/s41467-024-48525-5, published online 16 May 2024

The previous version of this Article did not acknowledge Ningning Liu, Xiaoyan Cui and Quanzhen Lv as corresponding authors. This has now been corrected in the HTML and PDF versions of the Article.

