# Author Correction: The vacuolar fusion regulated by HOPS complex promotes hyphal initiation and penetration in *Candida albicans*

**DOI:** 10.1038/s41467-024-52300-x

**Published:** 2024-09-13

**Authors:** Yu Liu, Ruina Wang, Jiacun Liu, Mengting Fan, Zi Ye, Yumeng Hao, Fei Xie, Ting Wang, Yuanying Jiang, Ningning Liu, Xiaoyan Cui, Quanzhen Lv, Lan Yan

**Affiliations:** 1 Center for Basic Research and Innovation of Medicine and Pharmacy (MOE), School of Pharmacy, Naval Medical University, Shanghai, 200433 PR China; 2 School of Chemistry and Molecular Engineering, East China Normal University, Shanghai, 200241 PR China; 3 School of Medicine, Tongji University, Shanghai, 200092 PR China; 4 State Key Laboratory of Systems Medicine for Cancer, Center for Single-Cell Omics, School of Public Health, Shanghai Jiao Tong University School of Medicine, Shanghai, 200025 PR China

**Keywords:** Fungal pathogenesis, Infection, Fungal infection

Correction to: *Nature Communications* 10.1038/s41467-024-48525-5, published online 16 May 2024

The original version of this Article contained an error in Fig. 2, in which the microscopy images were incorrectly labelled in panel f. This has been corrected in both the PDF and HTML versions of the Article.

Original Fig. 2f
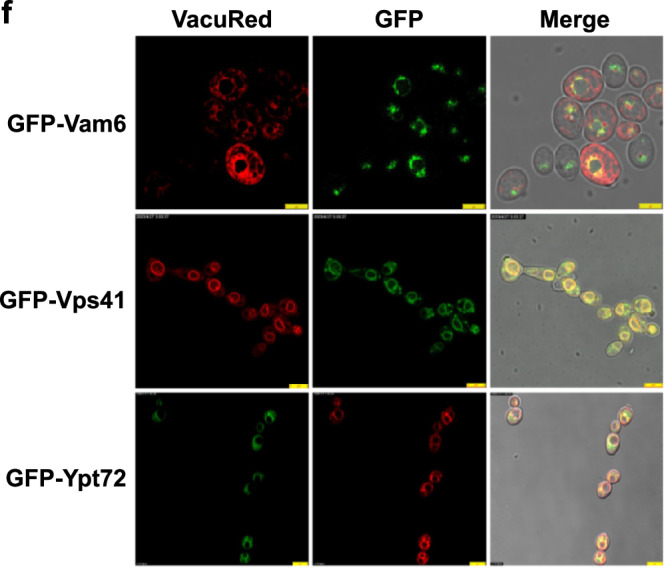


Updated Fig. 2f
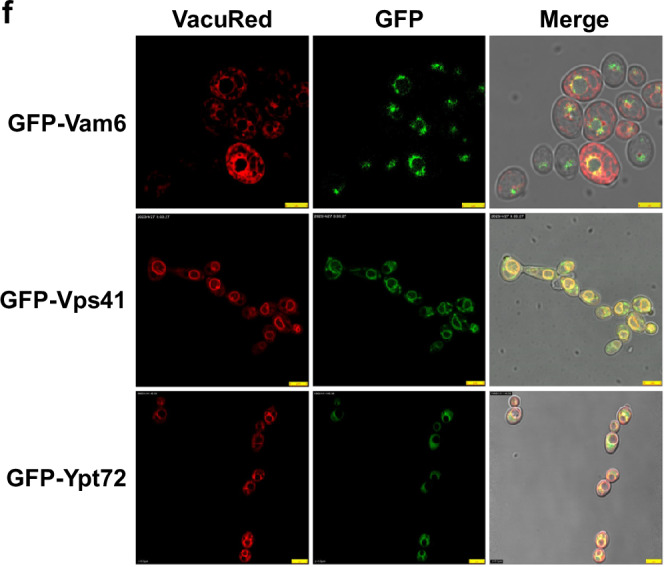


The original version of the Supplementary Information associated with this Article contained an error in Supplementary Fig. 2 in which there is a duplicate microscopy image of the hyphae of SC5314 in panel q, shown also in Fig. 2h of the main text. The HTML has been updated to include a corrected version of the Supplementary Information.

The original version of the Supplementary Information associated with this Article contained an error in Supplementary Fig. 4 in which colony images were duplicated in panel a. The HTML has been updated to include a corrected version of the Supplementary Information.

## Supplementary information

Updated Supplementary Information

