# Supplementary material for: The vacuolar fusion regulated by HOPS complex promotes hyphal initiation and penetration in Candida albicans
Source: Nat Commun. 2024 May 16;15:4131. doi: 10.1038/s41467-024-48525-5 (PMC11099166; doi:10.1038/s41467-024-48525-5)

The vacuolar fusion regulated by HOPS complex promotes hyphal initiation and penetration in *Candida albicans*

Supplementary Methods

Strain constructions

Strain *vam6*Δ/Δ. Strain *vam6*Δ/Δ was constructed using SC5314 *his1*$^{-}$ as the parent strain. Transformation mix contained a *VAM6* sgRNA expression cassette created using primers LY22-13 and LY22-14, a *NAT1* sgRNA expression cassette created using primers LY20-13 and LY20-14, Cas9, and an *CdHIS1* deletion cassette amplified from pMH01 and pMH02 using primers LY19-7 and LY19-8 which contain approximately 80 bp of homology upstream or downstream of the *VAM6* coding region. Transformants were selected on CSM media lacking histidine. The presence of the product of primers LY22-17 and LY20-11, and the absence of the product of primers LY22-17 and LY22-18, were used to evaluate the correct strain by genomic PCR. Gel images were obtained on the Tanon-2500B with the AllDoc_x software.

Strain *vam6*Δ/Δ *VAM6*. A *VAM6* coding cassette was amplified from SC5314 genomic DNA using primers LY19-54 and LY19-55, containing concatenating homology to a *NAT1* marker. A *NAT1* marker was then amplified from pNAT using LY21-62 and LY21-63. The above products were transformed into the *vam6* null mutant and selected for nourseothricin resistance. The presence of the product of primers LY20-17 and LY22-18 was used to confirm the correct re-introduction of *VAM6* by genomic PCR.

Strain *vps41*Δ/Δ. Strain *vps41*Δ/Δ was constructed using SC5314 *his1*$^{-}$ as the parent strain. Transformation mix contained a *VPS41* sgRNA expression

cassette created using primers LY20-45 and LY20-46, a *NAT1* sgRNA expression cassette created using primers LY20-13 and LY20-14, Cas9, and an *CdHIS1* deletion cassette amplified from pMH01 and pMH02 using primers LY20-49 and LY20-50 which contain approximately 80 bp of homology upstream or downstream of the *VPS41* coding region. Transformants were selected on CSM media lacking histidine. The presence of the product of primers LY20-55 and LY20-11, and the absence of the product of primers LY20-55 and LY20-56, were used to evaluate the correct strain by genomic PCR.

Strain *vps41*Δ/Δ *VPS41*. A *VPS41* coding cassette was amplified from SC5314 genomic DNA using primers LY21-70 and LY21-71, containing concatenating homology to a *NAT1* marker. A *NAT1* marker was then amplified from pNAT using LY21-62 and LY21-63. The above products were transformed into the *vps41* null mutant and selected for nourseothricin resistance. The presence of the product of primers LY20-17 and LY20-56 was used to confirm the correct re-introduction of *VPS41* by genomic PCR.

Strain *ypt72*Δ/Δ. Strain *ypt72*Δ/Δ was constructed using SC5314 *his1*$^{-}$ as the parent strain. Transformation mix contained a *YPT72* sgRNA expression cassette created using primers LY20-67 and LY20-68, a *NAT1* sgRNA expression cassette created using primers LY20-13 and LY20-14, Cas9, and an *CdHIS1* deletion cassette amplified from pMH01 and pMH02 using primers LY20-71 and LY20-72 which contain approximately 80 bp of homology upstream or downstream of the *YPT72* coding region. Transformants were selected on CSM media lacking histidine. The presence of the product of primers LY20-73 and LY20-11, and the absence of the product of primers LY20-73 and LY20-74, were used to evaluate the correct strain by genomic PCR.

Strain *ypt72*Δ/Δ *YPT72*. A *YPT72 coding* cassette was amplified from SC5314 genomic DNA using primers LY21-72 and LY21-73, containing concatenating

homology to a *NAT1* marker. A *NAT1* marker was then amplified from pNAT using LY21-62 and LY21-63. The above products were transformed into the *ypt72* null mutant and selected for nourseothricin resistance. The presence of the product of primers LY20-17 and LY20-74 was used to confirm the correct re-introduction of *YPT72* by genomic PCR.

Strain GFP-Vam6. The product was first amplified by primers LY21-122 and LY21-123 using the plasmid pCPC160 as a template, which was then used as a template to amplify by primers LY21-124 and LY21-125. Such product was transformed into SN152 (his1$^-$ leu2$^-$ arg4$^-$) selected on CSM media lacking arginine. The presences of the product of primers LY21-126 and LY21-103, and the primers LY21-127 and LY21-104, were used to confirm the correct construction of N-terminal GFP-fused *VAM6* by genomic PCR.

Strain GFP-Vps41. The product was amplified by primers LY23-33 and LY23-34 using the plasmid pCPC158 as a template, which was then transformed into SN152 (his1$^-$ leu2$^-$ arg4$^-$) selected on CSM media lacking leucine. The presence of the product of primers LY23-35 and LY21-103, and the primers LY20-56 and LY21-105, were used to confirm the correct construction of N-terminal GFP-fused *VPS41* by genomic PCR.

Strain GFP-Ypt72. The product was amplified by primers LY23-36 and LY23-37 using the plasmid pCPC158 as a template, which was then transformed into SN152 (his1$^-$ leu2$^-$ arg4$^-$) selected on CSM media lacking leucine. The presence of the product of primers LY20-73 and LY21-103, and the primers LY20-74 and LY21-105, were used to confirm the correct construction of N-terminal GFP-fused *YPT72* by genomic PCR.

Strain *GTR1*^{OE} *vam6*Δ/Δ. The plasmid pJK1277 (kindly provided by Prof. Julia Koehler) was linearized with BsrGI enzyme, and then transformed into the *vam6* null mutant screened by nourseothricin resistance. The presence of the product of primers LY22-50 and LY22-51 was used to confirm the correct construction by genomic PCR.

Strain GFP-Yvc1 *vam6*Δ/Δ. Strain GFP-Yvc1 *vam6*Δ/Δ was constructed using strain GFP-Yvc1 as the parent strain. Transformation mix contained a *VAM6* sgRNA expression cassette created using primers LY22-13 and LY22-14, Cas9, and a *NAT* deletion cassette amplified from pNAT using primers LY22-19 and LY22-20 which contain approximately 80 bp of homology upstream or downstream of the *VAM6* coding region. Transformants were selected on CSM media containing nourseothricin. The presence of the product of primers LY22-17 and LY20-15, and the absence of the product of primers LY22-17 and LY22-18, were used to evaluate the correct strain by genomic PCR.

Strain *vac8*Δ/Δ. Strain *vac8*Δ/Δ was constructed using SC5314 *his1*$^{-}$ as the parent strain. Transformation mix contained a *VAC8* sgRNA expression cassette created using primers LY23-142 and LY23-143, Cas9, and an *CdHIS1* deletion cassette amplified from pMH01 and pMH02 using primers LY23-144 and LY23-145 which contain approximately 80 bp of homology upstream or downstream of the *VAC8* coding region. Transformants were selected on CSM media lacking histidine. The presence of the product of primers LY23-146 and LY20-11, and the absence of the product of primers LY23-146 and LY23-147, were used to evaluate the correct strain by genomic PCR.

Vacuole staining by FM4-64

Vacuole morphology in yeast and hyphal cells were visualized using lipophilic styryl dye FM4-64 (Invitrogen, T3166) as reported before with minor modifications[1]. *C. albicans* strains grown overnight were adjusted to an OD_{600} of 0.5 in fresh YPD medium and allowed to grow for 90-120 min. *C. albicans* strains were then incubated with FM4-64 (32 μM) for 20 min at 30 °C in a humidified incubator and washed with fresh YPD three times. The stained strains were then grown in YPD medium for 60 min at 30 °C as yeast-form cells, or in RPMI 1640 plus 10% FBS medium for 90-120 min at 37 °C to induce the hyphae. The dye was excited by a 558 nm laser and fluorescence was collected at 734 nm. Fluorescence of FM4-64 was acquired on a Leica TCS SP5 II confocal microscope. Images were processed using LAS AF Lite (2.6.1 7314).

Time-growth curve assay

C. albicans strains were incubated to the exponential growth phase, and then resuspended in YPD medium with a starting OD_{600nm} of 0.1. Strains were incubated at 30 °C for 24 h. OD_{600} values were recorded at 0, 2, 4, 6, 8, 10, 12, and 24 h using a Tecan plate reader (Infinite F200 PRO, Switzerland).

Supplementary Table 1. *C. albicans* strains and plasmids used in this study.

Strains	Parental Strain	Genotype	Notes
LY849		SC5314 wild type	SC5314 wild type strain
LY803	LY849	SC5314 *his1Δ::r3NAT1r3/his1Δ::r3NAT1r3*	*his1Δ/his1Δ*
LY817	LY803	*his1Δ::r3NAT1r3/his1Δ::r3NAT1r3 VAM6Δ::r1HIS1r1/VAM6Δ::r1HIS1r1*	*vam6Δ/Δ*
LY857	LY803	*his1Δ::r3/his1Δ::r3 VAM6Δ::r1HIS1r1/VAM6Δ::r1HIS1r1*	*vam6Δ/Δ*
LY864	LY857	*his1Δ::VAM6::r3NAT1r3/his1Δ::VAM6::r3NAT1r3 VAM6Δ::r1HIS1r1/VAM6Δ::r1HIS1r1*	*vam6Δ/Δ VAM6* ectopic expression strain
LY943	SN152	*arg4/arg4; leu2/leu2; his1/his1; URA3/ura3 imm434; IRO1/iro1 imm434 ARG4::pADH1-GFP VAM6*	GFP Vam6
LY954	LY857	*his1Δ::r3/his1Δ::r3 VAM6Δ::r1HIS1r1/VAM6Δ::r1HIS1r1 pACT1-GTR1*	GTR1^{OE} *vam6Δ/Δ*
LY953	NKF05	*ura3Δ:: imm434/ura3Δ:: imm434 his1::HisG/His1::HisG arg4::HisG/arg4::HisG pACT1-YVC1-GFP vam6Δ::NAT/vam6Δ::NAT*	Yvc1-GFP *vam6Δ/Δ*
LY885	LY803	*his1Δ::r3NAT1r3/his1Δ::r3NAT1r3 VPS41Δ::r1HIS1r1/VPS41Δ::r1HIS1r1*	*vps41Δ/Δ*
LY924	LY803	*his1Δ::r3/his1Δ::r3 VPS41Δ::r1HIS1r1/VPS41Δ::r1HIS1r1*	*vps41Δ/Δ*
LY929	LY924	*his1Δ::VPS41::r3NAT1r3 VPS41Δ::r1HIS1r1/VPS41Δ::r1HIS1r1*	*vps41Δ/Δ VPS41* ectopic expression strain
LY974	SN152	*arg4/arg4; leu2/leu2; his1/his1; URA3/ura3 imm434; IRO1/iro1 imm434 LEU2::pADH1-GFP VPS41*	GFP Vps41
LY905	LY803	*his1Δ::r3NAT1r3/his1Δ::r3NAT1r3 YPT72Δ::r1HIS1r1/YPT72Δ::r1HIS1r1*	*ypt72Δ/Δ*
LY925	LY803	*his1Δ::r3/his1Δ::r3 YPT72Δ::r1HIS1r1/YPT72Δ::r1HIS1r1*	*ypt72Δ/Δ*
LY931	LY925	*his1Δ::YPT72::r3NAT1r3 YPT72Δ::r1HIS1r1/YPT72Δ::r1HIS1r1*	*ypt72Δ/Δ YPT72* ectopic expression strain
LY973	SN152	*arg4/arg4; leu2/leu2; his1/his1; URA3/ura3 imm434; IRO1/iro1 imm434 LEU2::pADH1-GFP YPT72*	GFP Ypt72

LY982		*his1Δ::r3NAT1r3/his1Δ::r3NAT1r3 VAC8Δ::r1HIS1r1/VAC8Δ::r1HIS1r1*	*vac8*Δ/Δ
**Plasmid**	**Resistance**	**Description**	
pV1093	Ampicillin	used to amplify the CAS9 expression and sgRNA expression cassettes	
pMH01	Ampicillin	used to amplify the *Candida dubliniensis HIS1* marker	
pMH02	Ampicillin	used to amplify the *Candida dubliniensis HIS1* marker	
pNAT	Ampicillin	used to amplify the nourseothricin-resistance marker (*NAT*)	
pCPC160	Ampicillin	used to amplify the fragment containing *ARG4* marker and GFP	
pCPC158	Ampicillin	used to amplify the fragment containing *LEU2* marker and GFP	
pJK1277	Ampicillin	used to overexpress *GTR1*	

Supplementary Table 2. All primers used in this study.

Number	Name	Sequence	Description
LY20-1		ATCTCATTAGATTTGGAACTTGTGGGTT	Forward primer for amplification of *Cacas9* cassette
LY20-2		TTCGAGCGTCCCAAAACCTTCT	Reverse primer for amplification of *Cacas9* cassette
LY20-3		GCGCAAGAAGCCTCAACT	Forward primer for amplification of target gene deletion cassette
LY20-4		GAGCTACAGGGCTTGACC	Reverse primer for amplification of target gene deletion cassette
LY20-7		ACAAATATTTAAACTCGGGACCTGG	Reverse primer for amplification of sgRNA scaffold
LY20-8		AAGAAAGAAAGAAAACCAGGAGTGAA	Forward primer for amplification of SNR52 promoter
LY20-9		GCGGCCGCAAGTGATTAGACT	Forward nested primers for third round PCR for construction of sgRNA expression cassette
LY20-10		GCAGCTCAGTGATTAAGAGTAAAGATGG	Reverse nested primers for third round PCR for construction of sgRNA expression cassette
LY20-11	CdHIS1 Int Chk/R	GGCGCAACAGATATATTGGTGCTCG	Reverse primer to detect His expression cassette
LY20-13		GTCACGACGTTGTAAAACGAGTTTTAGAGCTAGAAATAGCAAGTTAAA	Forward primer for amplification of sgRNA scaffold with overlapping *NAT1* guide sequence
LY20-14		TCGTTTTACAACGTCGTGACCAAATTAAAAATAGTTTACGCAAGTC	Reverse primer for amplification of SNR52 promoter with overlapping *NAT1* guide sequence
LY20-15	NAT1 Int Chk/R	TCAATGGTGGATCAACTGGAACTTC	Reverse primer to detect Nat expression cassette
LY20-16	NAT1 Chk/F	GGCTACTGAATTCGCCAGAG	Forward primer to detect Nat expression cassette
LY20-17	CaHIS1 UP Chk/F	GGACTCGACAGGTACCTGGAGGATGAG	Forward primer to detect His expression cassette
LY21-62	Nat Re/F	TTTCCCAGTCACGACGTTGTAAAACGACGGCCAGTGAA	Forward primer for amplification of NAT cassette

		TT	
LY21-63	Nat Re/R	TGGTATAAGACAGATTGAGTCAAATTGAAGTAGATCTATAAGTATATATGTATAATATTTATGAGAAACTATCACTTCTTGTGGAATTGTGAGCGGATA	Reverse primer for amplification of NAT cassette
LY21-103	CmLeu Int Chk/R	GCGGCCGTTACTAGTGGAT	Reverse primer to detect Leu2 or Arg4 expression cassette
LY21-104	GFP Int Chk/F	GGCTGACAAACAAAAGAATGG	Forward primer to detect Gfp expression cassette
LY21-105	pADH1 Int Chk/F	TCCCTGGTCTTATCTTCTCCAG	Forward primer to detect Promoter-Adh1 expression cassette
LY22-13		TGACTTTGGTGTGTCTTCGTGTTTTAGAGCTAGAAATAGCAAGTTAAA	Forward primer for amplification of sgRNA scaffold with overlapping *VAM6* guide sequence
LY22-14		ACGAAGACACACCAAAGTCACAAATTAAAAATAGTTTACGCAAGTC	Reverse primer for amplification of SNR52 promoter with overlapping *VAM6* guide sequence
LY19-7		ATAGCAAAACCTAAAGAACTGGAGTTTCTATGTCATCAACATTGTACATTTTCTATCCGTGTATCGAATAAAGAGATTAACTCGAGGTCGACGGTATCG	Forward primer for amplification of *VAM6* deletion cassette
LY19-8		TATTAATATAAAACTGTAAATATGAGACTTTTTAAAAATTACTTGTATGATAAGATATATATTTAATTAAACTAACGTATCCAATACGCAAACCGCC	Reverse primer for amplification of *VAM6* deletion cassette
LY22-17	VAM6 Chk/F	CTTTGGAAGAATACAGACAGTT	Forward primer at the upstream of *VAM6* ORF
LY22-18	VAM6 Int Chk/R	CAATTTCAAATATCTCGAGGGA	Reverse primer in the *VAM6* ORF
LY19-54	VAM6 Re/F	CCATTGAAATTTTTTATTTTTTTTTGGTGAAGATTTTTCCCACACAACTTCTTCTTTTACTTAACCACCAACTACCGATATTTGAACAATTGGTTATATATAGTCTTCAATGTATATT	Forward primer for amplification of *VAM6* ORF for re-introduction
LY19-55	VAM6 Re/R	CGACGTCGGGCCCAATTCGCCCTATAGTGAGTCGTATTACAATTCACTGGCCGTCGTTTTACAACGTCGTGACTGGG	Reverse primer for amplification of *VAM6* ORF for re-introduction

		AAAAGTTGAAGTAATCTCACAAGATACAAACAA	
LY21-122	VAM6 F1	AAATTCAAAGCTAAACCCAACCAGCACAGACGGAAGAGATGCCACCTGACGTCTAAGAAA	Forward primer for amplification of the upstream of marker
LY21-123	VAM6 R1	TCTATCCAAAGGCTCTATCACATCTATATTCATTACCATCTGCGTTATCCTCGAGTTCTT	Forward primer for amplification of the upstream of marker
LY21-124	VAM6 F2	AATTACACGTCCTACAGATGCATTGTTTGTTTAAGGAAAAATTCAAAGCTAAACCCAAC	Forward primer for amplification of the upstream of marker
LY21-125	VAM6 R2	TATCTTGTTATTCTTAATCTCATTTGAAGAAATTCTTATTCTATCCAAAGGCTCTATCAC	Forward primer for amplification of the upstream of marker
LY21-126	VAM6 GFP Chk/F	GCTGGAGAGAGTGGACAATTT	Forward primer at the upstream of *VAM6* ORF to detect Gfp expression cassette
LY21-127	VAM6 Int Chk/R	CAACAAAAAGCTGCTGTTGCT	Reverse primer to detect Gfp expression cassette
LY22-50	GTR1 Chk/F	GCTCCAGAACATAGAGGTCAT	
LY22-51	NAT Chk/R with GTR1	TATCTGCCAGGGGCAAAAGTA	
LY 22-19	VAM6 NAT1 Adap/F	ATAGCAAAACCTAAAGAACTGGAGTTTCTATGTCATCAACATTGTACATTTTCTATCCGTGTATCGAATAAAGAGATTAATTTCCCAGTCACGACGTT	Forward primer for amplification of *VAM6* deletion cassette
LY 22-20	VAM6 NAT1 Adap/R	TATTAATATAAAACTGTAAATATGAGACTTTTTAAAAATTACTTGTATGATAAGATATATATTTAATTAAACTAACGTATGTGGAATTGTGAGCGGATA	Reverse primer for amplification of *VAM6* deletion cassette
LY20-45		AAAAAATTATTTGCAAGCAGTTTTAGAGCTAGAAATAGCAAGTTAAA	Forward primer for amplification of sgRNA scaffold with overlapping *VPS41* guide sequence
LY20-46		TGCTTGCAAATAATTTTTTCAAATTAAAAATAGTTTACGCAAGTC	Reverse primer for amplification of SNR52 promoter with overlapping *VPS41* guide sequence
LY20-49		GCATAATTGTTGTAGTTTACATTTTTTTAAGAGCCTACT	Forward primer for amplification of *VPS41* deletion

		AGCATAACCACTAGCAGTAATCAATCACCTTGCCAAACTACTCGAGGTCGACGGTATCG	cassette
LY20-50		TAAACTAATTTTTGAAATGCTAACCAGACCCAACATCATTTATGATATACACTGCCACTTTTGTATGGCTGAGACTTTGTCCAATACGCAAACCGCC	Reverse primer for amplification of *VPS41* deletion cassette
LY20-55	VPS41 Chk/F	AATCATGTGGCCAGTTAGGC	Forward primer at the upstream of *VPS41* ORF
LY20-56	VPS41 Int Chk/R	CACAACTGTTCCATCCATCG	Reverse primer in the *VPS41* ORF
LY21-70	VPS41 Re/F	CCATTGAAATTTTTATTTTTTTTGGTGAAGATTTTTCCCACACAACTTCTTCTTTTACTTAACCACCAACTACCGATATGGTAAAAACTGAACTTTGGTTTAGATTTG	Forward primer for amplification of *VPS41* ORF for re-introduction
LY21-71	VPS41 Re/R	CGACGTCGGGCCCAATTCGCCCTATAGTGAGTCGTATTACAATTCACTGGCCGTCGTTTTACAACGTCGTGACTGGGAAATTTGATTTGACTCTTAGATATATAGTGTTCACTTG	Reverse primer for amplification of *VPS41* ORF for re-introduction
LY23-33	VPS41 F	AGAGAGAGAAGAAGACCGCGACAAATTTAATAGTGAGAAACGAACAATGCCACCATAAATACGTATAGAAAATCTTTATGCCACCTGACGTCTAAGAAA	Forward primer for amplification of the upstream of marker
LY23-34	VPS41 R	AATCCTTGACAATTGACTCTTCATGTTCATTTTCTGCCGTTATTTCATTCGATTCTTTGGAGATATTGGTATCTGTCATCTGCGTTATCCTCGAGTTCT	Reverse primer for amplification of the upstream of marker
LY23-35	VPS41 GFP Chk/F	CGGGGSYTTTGACTATGGTGG	Forward primer at the upstream of *VPS41* ORF to detect Gfp expression cassette
LY20-67		CATCAATATAAAGCGACTATGTTTTAGAGCTAGAAATAGCAAGTTAAA	Forward primer for amplification of sgRNA scaffold with overlapping *YPT72* guide sequence
LY20-68		ATAGTCGCTTTATATTGATGCAAATTAAAAATAGTTTACGCAAGTC	Reverse primer for amplification of SNR52 promoter with overlapping *YPT72* guide sequence
LY20-71		TATATCATTGAACGATACAGAGTTTAATTTAATTTAATTC	Forward primer for amplification of *YPT72* deletion

		AATTCAATTTAATTAATTAATCATATACACTTAATTTTCACTCGAGGTCGACGGTATCG	cassette
LY20-72		AAACAACAATTATAAACGAATTCGTGATATCTATTTGTTCTCTTTCTTCACCTGTGTATTTGAAATTGATTGTTATATTACCAATACGCAAACCGCC	Reverse primer for amplification of *YPT72* deletion cassette
LY20-73	YPT72 GFP Chk/F	GGTCCTCCAGGAACCAATGC	Forward primer at the upstream of *YPT72* ORF to detect Gfp expression cassette
LY20-74	YPT72 Int Chk/R	CTTGACCAGCGGTATCCCAG	Reverse primer in the *YPT72* ORF
LY21-72	YPT72 Re/F	CCATTGAAATTTTTATTTTTTTTGGTGAAGATTTTCCCACACAACTTCTTCTTTTACTTAACCACCAACTACCGATATGCACAAATTTTAATCCTGACCAAAACGCA	Forward primer for amplification of *YPT72* ORF for re-introduction
LY21-73	YPT72 Re/R	CGACGTCGGGCCCAATTCGCCCTATAGTGAGTCGTATTACAATTCACTGGCCGTCGTTTTACAACGTCGTGACTGGGAAAGTAAAAAAGGATATTTGAAATTAGAATGTATATTAG	Reverse primer for amplification of *YPT72* ORF for re-introduction
LY23-36	YPT72 F	GGCTAATTGGAGTAATTAATATTCCTTTCACTCCAAGCTATACATAATACAATTATACACTTGCAATTGATACTATTATGCCACCTGACGTCTAAGAAA	Forward primer for amplification of the upstream of marker
LY23-37	YPT72 R	TAAGTGAGGTTTTACCAACACCAGAGTCTCCTAATATAATGACTTTTAATAATGTTTTCTTTCTAGATGATGATGACATCTGCGTTATCCTCGAGTTCT	Reverse primer for amplification of the upstream of marker
LY22-70	VAM6/F	TGTGCTACCCAGGTCGTTTC	Forward prime for real time RT-PCR
LY22-71	VAM6/R	CTTTGACTTGTTCATCGGTGTT	Reverse primer for real time RT-PCR
LY22-72	GTR1/F	CGACGTTTAGGTGCCACTATT	Forward prime for real time RT-PCR
LY22-73	GTR1/R	TGTTTGACCTCCACAATCCC	Reverse primer for real time RT-PCR
LY23-142		GCAACGGCAGCTAAAACTAAGTTTTAGAGCTAGAAATAGCAAGTTAAA	Forward primer for amplification of sgRNA scaffold with overlapping *VAC8* guide sequence

LY23-143		TTAGTTTTAGCTGCCGTTGCCAAATTAAAAATAGTTTACGCAAGTC	Reverse primer for amplification of SNR52 promoter with overlapping *VAC8* guide sequence
LY23-144		TTTTAATCTTCATCATACATAAATAACAACAACAACAACAACAACAACAATCGCATAACAACAATCAAGAAGATATCAAACTCGAGGTCGACGGTATCG	Forward primer for amplification of *VAC8* deletion cassette
LY23-145		ATAAATATATTGATATATATATATATATACATATATATAATTTATATATTTATCCCACTTAGAACTGTTGGTCCTTTCTCCCAATACGCAAACCGCC	Reverse primer for amplification of *VAC8* deletion cassette
LY23-146	VAC8 Chk/F	GCGGGATTAAACAACAAACAACA	Forward primer at the upstream of *VAC8* ORF
LY23-147	VAC8 Int Chk/R	CTTCGGAATCAGCCAGTTGT	Reverse primer in the *VAC8* ORF

Supplementary Figure legends

Supplementary Fig. 1. Strain constructions of the *vam6* null mutant. For detailed explanations of the techniques, please see Supplementary methods section. **a** Disruption of target gene in one step by CRISPR-Cas9 system. Small arrows represent orientation and approximate position of primers (Supplementary Table 2) used for PCR and confirmation of the disruption. **b** PCR confirmation of the disruption of *VAM6* by genomic DNA. The *vam6* mutant (*vam6*Δ/Δ) and the parental strain (C) were analyzed by genomic DNA amplified with the primers indicated at the top of the electrophoresis gel image. Red arrows indicate the correct construction.

Supplementary Fig. 2. Reintegration of the target genes restored the vacuole morphology and hyphal formation. a Reintegration of the target gene at the *CaHIS1* locus. **b-c** PCR confirmation of the disruption of *VPS41* (**b**) or *YPT72* (**c**) by genomic DNA. **d-f** The re-introduction of *VAM6* (*vam6*Δ/Δ *VAM6*) (**d**), *VPS41* (*vps41*Δ/Δ *VPS41*) (**e**), and *YPT72* (*ypt72*Δ/Δ *YPT72*) (**f**). **g** Imaging of vacuole membrane stained by FM4-64. Scale bars = 10 μm. **h** Imaging of vacuole membrane stained by VacuRed. Scale bars = 10 μm. **i** Imaging of vacuole compartment stained by quinacrine. Scale bars = 7.5 μm. The representative images shown are from 3 biological replicates (**g**, **h**, **i**). **j** Application of N-terminal GFP fusion system by using of pCPC160. **k** PCR confirmation of GFP fusion at the N-terminal of *VAM6*. **l** Application of N-terminal GFP fusion system by using of pCPC158. **m-n** PCR confirmation of GFP fusion at the N-terminal of *VPS41*(**m**) and *YPT72* (**n**). **o** Disruption of *VAM6* gene in one step by CRISPR-Cas9 system. **p** PCR confirmation of the disruption of *VAM6* by genomic DNA in the Yvc1-GFP strain. **q** The hyphal morphology of *C. albicans* strains stained by calcoflour white. *C. albicans* strains were cultured in liquid medium at 37 °C for 4 h. Scale bars = 50 μm. The length of hyphae was measured by Image J. Box plots indicate median (middle line), 25th, 75th percentile (box) and minimum and maximum value as well as outliers (single points). n = 50 hyphae. **r** Vertical sections of hyphal colonies on solid

media were observed by microscopy. The representative images shown are from 3 biological replicates The W and D values (mm) were quantified by Image J. Scale bars = 1 mm. Data were presented as mean ± SD; n = 3 colonies. Two-tailed unpaired t-test (**q**, **r**). Red arrows indicate the correct constructions (**b**, **c**, **d**, **e**, **f**, **k**, **m**, **n**, **p**). Small arrows represent orientation and approximate position of primers (Supplementary Table 2) used for PCR and confirmation of the disruption (**a**, **j**, **l**, **o**). Source data are provided as a Source Data file.

Supplementary Fig. 3. Reintegration of the target genes restored the pathogenicity. a The percentage of PI-positive murine peritoneal macrophages cells co-incubated with *C. albicans* strains for 3 h or 6 h (MOI=1). Data were presented as mean ± SD. n = 3 samples. **b** Murine peritoneal macrophages were co-incubated with *C. albicans* (MOI=1) for 3 h, stained with PI. The plate was photographed by fluorescence microscopy. Scale bars = 50 µm. **c** The survival of mice infected with *C. albicans* strains. n = 10 mice. **d** The fungal burden in kidneys of mice were determined 5 days after fungal inoculation. Data were presented as mean ± SD; n = 5 mice. **e** The kidneys of mice were taken out 5 days after fungal inoculation and made pathological sections with PAS staining. Scale bars = 100 µm. The hyphal length in kidneys was measured by Image J. n = 22 hyphae. **f** The exposed back skin with fungal-infected damage on day 7. **g** The fungal burden in damaged skin of mice were determined 3 days after fungal inoculation. Data were presented as mean ± SD; n = 5 mice. **h** The pathological images of infected skin stained by PAS. The dimension of skin damage was quantified by the maximum depth of penetrated hyphae measured by Image J. Scale bars = 100 µm. **i** The hyphal length in damaged skin was measured by Image J. n = 20 hyphae. Log-rank (Mantel-Cox) test (**c**), two-tailed unpaired t-test (**a**, **d**, **e**, **g**, **i**). Box plots indicate median (middle line), 25th, 75th percentile (box) and minimum and maximum value as well as outliers (single points) (**e**, **i**). Source data are provided as a Source Data file.

Supplementary Fig. 4. Reintegration of the target genes restored the sensitivity to rapamycin. a SAPs secretion assay. *C. albicans* were spotted on the YCB-BSA agar and cultured at 30 °C for 48 h. The size of each halo was measured by Image J. Data were presented as mean ± SD; n = 3 colonies. **b** Rapamycin sensitivity examined by spot assay. *C. albicans* strains were serial 5-fold diluted, spotted on YPD agar containing 2.5 nM rapamycin and cultured at 30 °C for 48 h. **c-d** The phosphorylation of RPS6 protein (P-S6) examined by western blotting. The representative images shown are from 3 biological replicates. **e** The expression of *ALS1*, *ALS3*, *ALS5*, *HWP1*, *ECE1*, *SAP2* and *SAP6* examined by quantitative real time RT-PCR in the *C. albicans* strains co-cultured with or without HUVECs for 1.5 h or 3 h compared to the wild type SC5314 at the time point of 0 h. Fungal mRNA was extracted from *C. albicans* incubated with/without HUVECs (MOI=1) cultured in DMEM medium for 1.5 h or 3 h. Data were presented as mean ± SD; n = 3 independent biological repeats. **f** The growth curves in *C. albicans* in liquid YPD medium. **g** The hyphal morphology and length in *C. albicans* SC5314 in liquid media with or without rapamycin. The wild type SC5314 was cultured in RPMI 1640 + 10% FBS or Spider liquid medium containing different concentrations of rapamycin at 37 °C for 4 h. The hyphal length was quantified by Image J. Box plots indicate median (middle line), 25th, 75th percentile (box) and minimum and maximum value as well as outliers (single points); n = 30 hyphae. **h** Agarose gel electrophoresis images of $GTR1^{OE}$ - *vam6*Δ/Δ confirmed by genomic PCR. The primers used are labeled above this gel. Colony names are labeled above each lane. M, DNA markers. C, the control strain. Red arrows indicate the correct constructions. Two-tailed unpaired t-test (**a**, **e**, **g**). Source data are provided as a Source Data file.

Supplementary Fig. 5. Hyphal morphology in GFP-tagged *C. albicans* incubated with HUVECs. a. *C. albicans* Yvc1-GFP was co-incubated with HUVECs for 3 h. Red arrows indicate the small dense vacuoles at the tips of hyphae that interacted with HUVECs. Scale bars = 7.5 µm. **b** Vacuoles stained by VacuRed in hyphae scraped from Spider agar. *C. albicans* strains were spotted on Spider agar at 37 °C for 48 h, scraped, and then stained with 500 nM

VacuRed for 10 min. Scale bars = 7.5 µm. The representative images shown are from 3 biological replicates (**a**, **b**). **c** The hyphal morphology of *C. albicans* strains on Spider solid medium containing $CaCl_2$. *C. albicans* strains were spotted on the Spider solid medium containing 10 mM or 50 mM of $CaCl_2$, and incubated at 37 °C for 5 days. The edge of hyphal colonies were observed by microscope under 4× objective. Scale bars = 1000 µm. **d-e** Parameters C and P of hyphal colonies in **Fig.5g and Fig.5i**, which were on Spider + $CaCl_2$ or RPMI 1640 + 10% FBS solid plates containing different concentrations of agar**,** were measured by Image J. Data were presented as mean ± SD; n = 13 colonies (**d**); n = 7 colonies (**e**). Two-tailed unpaired t-test (**d**, **e**). Source data are provided as a Source Data file.

Supplementary Fig. 6. Strain constructions of the *vac8*Δ/Δ strain. For detailed explanations of the techniques, please see Supplementary Methods section. **a** Disruption of target gene in one step by CRISPR-Cas9 system. Small arrows represent orientation and approximate position of primers (Supplementary Table 2) used for PCR and confirmation of the disruption. **b** PCR confirmation of the disruption of *VAC8* by genomic DNA. The deletion of *VAC8* and the parental strain SC5314 *His1*$^-$ (C) were analyzed by genomic DNA amplified with the primers indicated at the top of the electrophoresis gel image. Red arrows indicate the correct constructions.

Supplementary Fig. 7. a Imaging of vacuole membrane stained by VacuRed. *C. albicans efg1*Δ/Δ *cph1*Δ/Δ mutants were cultured in RPMI 1640 + 10% FBS medium at 37 °C for 90 min, and stained with 500 nM VacuRed. Scale bars = 5 µm.

Supplementary References

1. Vida, T. A.; Emr, S. D., A new vital stain for visualizing vacuolar membrane dynamics and endocytosis in yeast. *J Cell Biol* **1995,** *128* (5), 779-92.

Supplementary Fig. 1

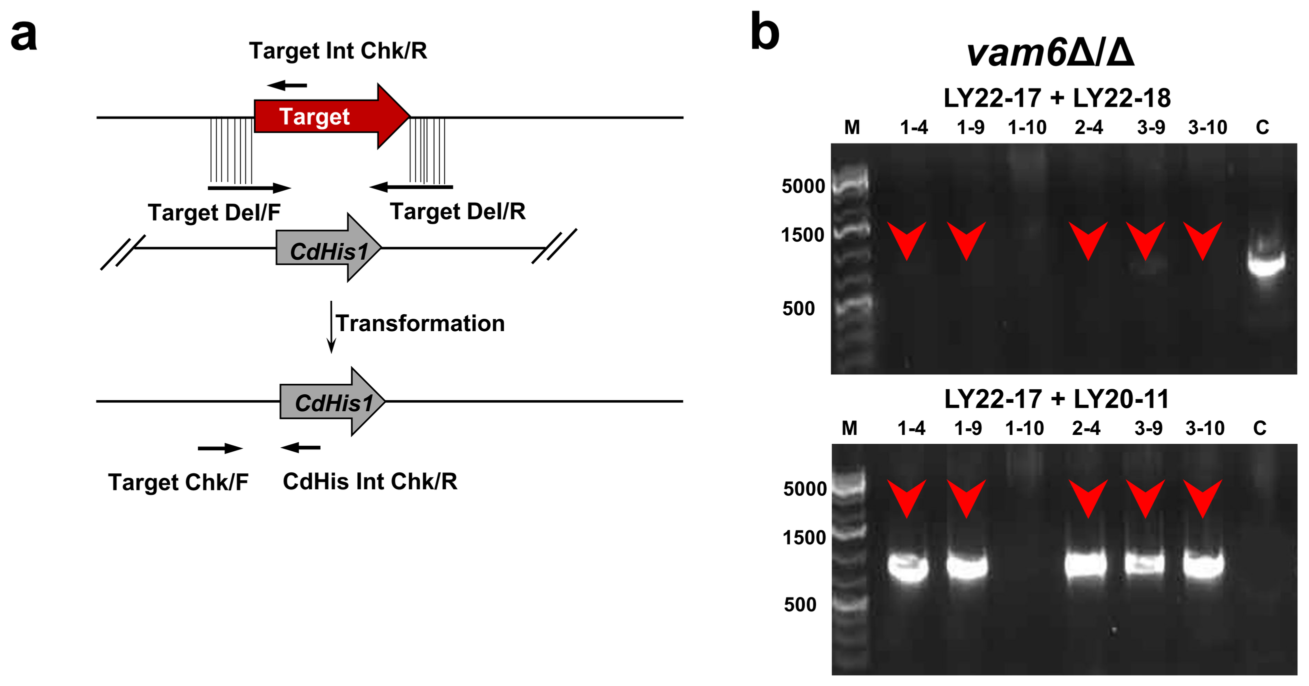

Supplementary Fig. 2

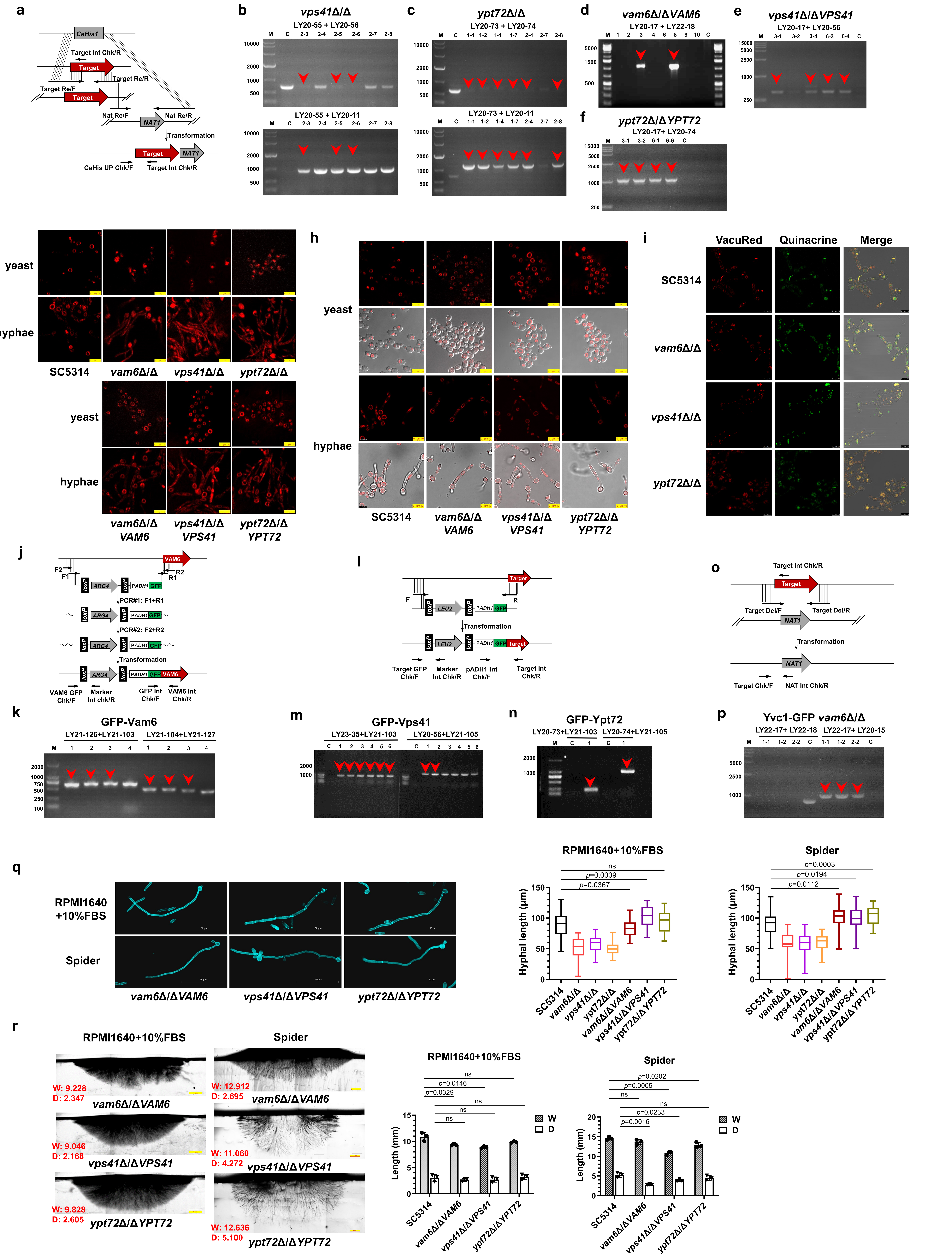

Supplementary Fig. 3

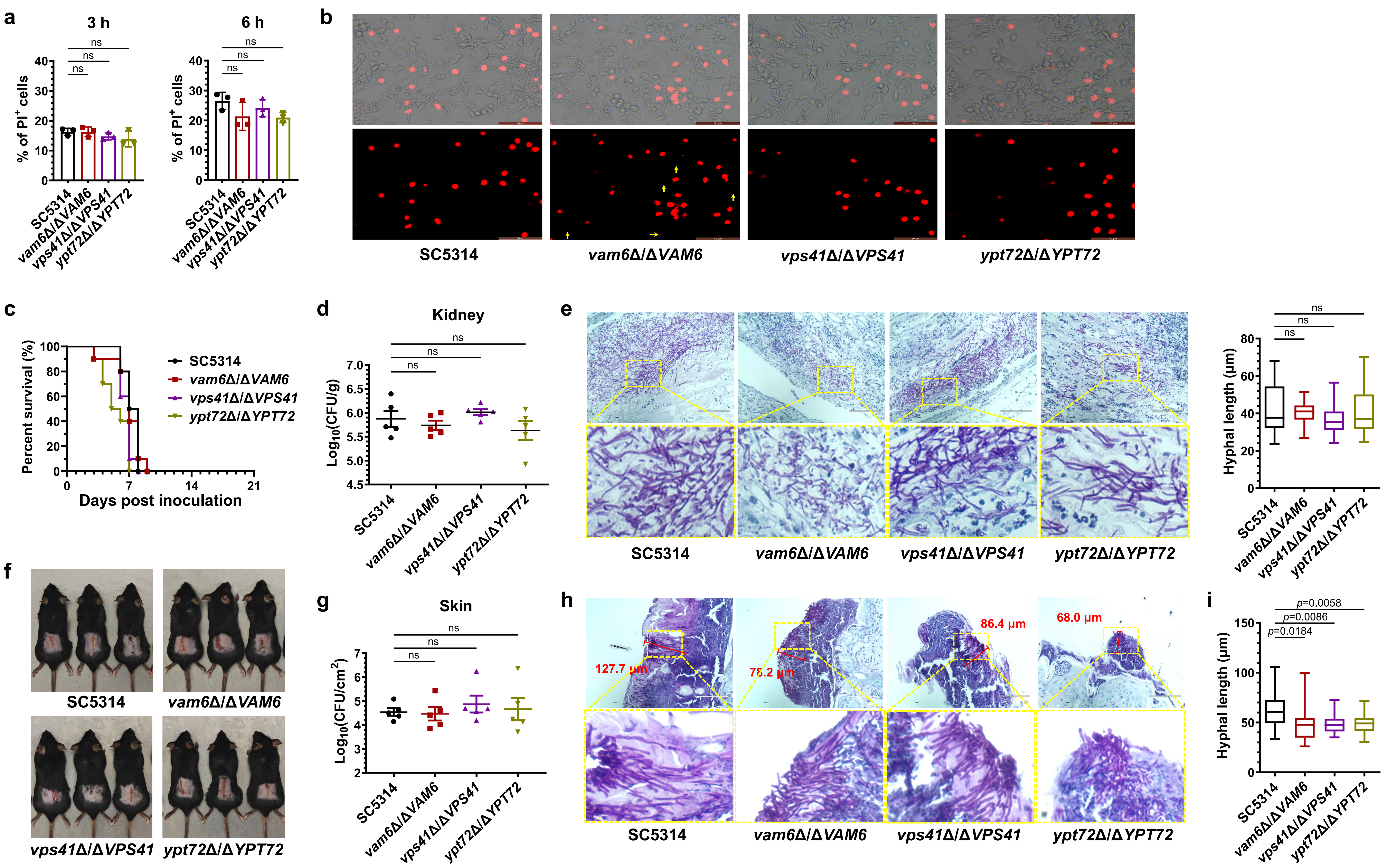

Supplementary Fig. 4

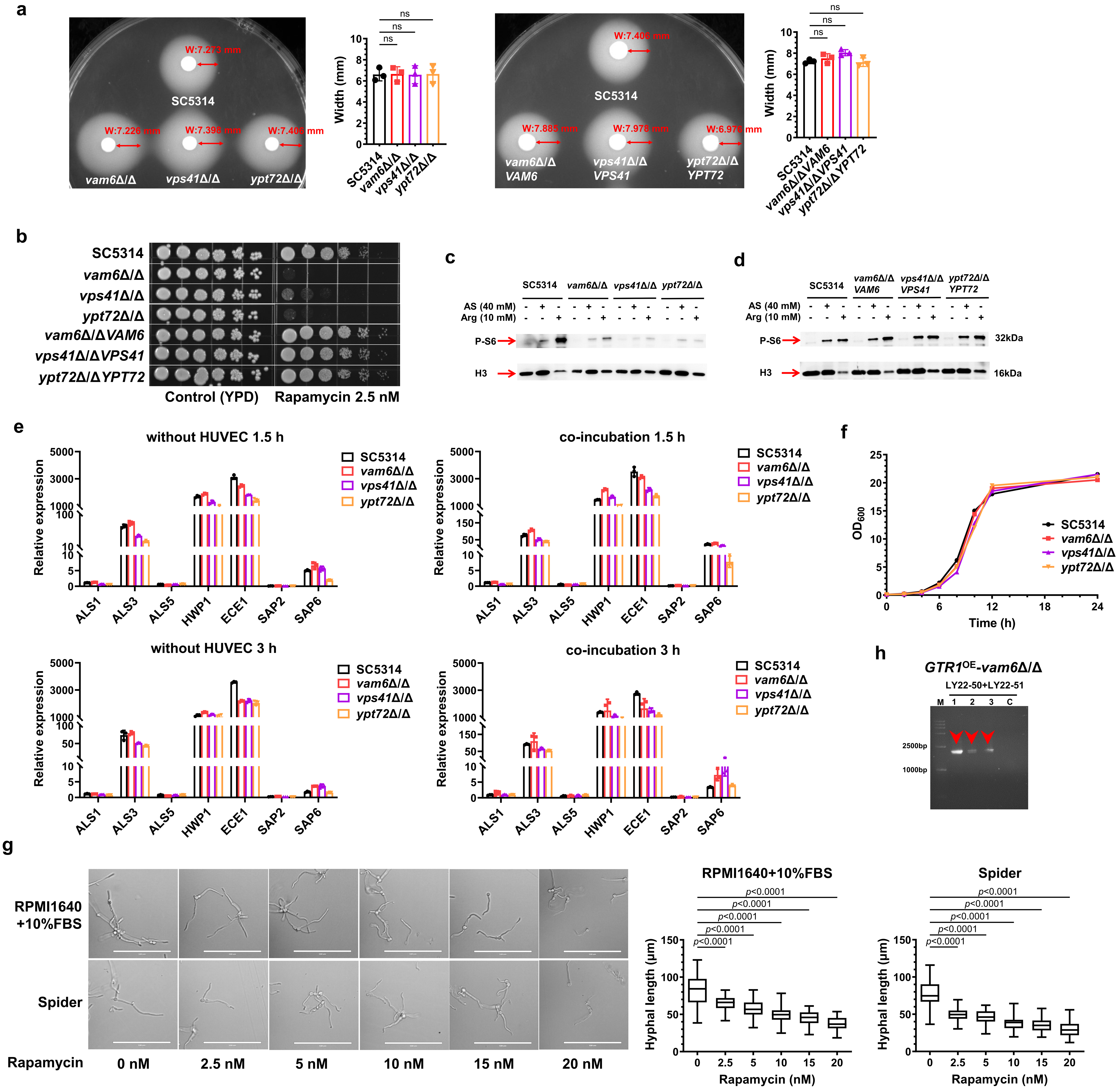

Supplementary Fig. 5

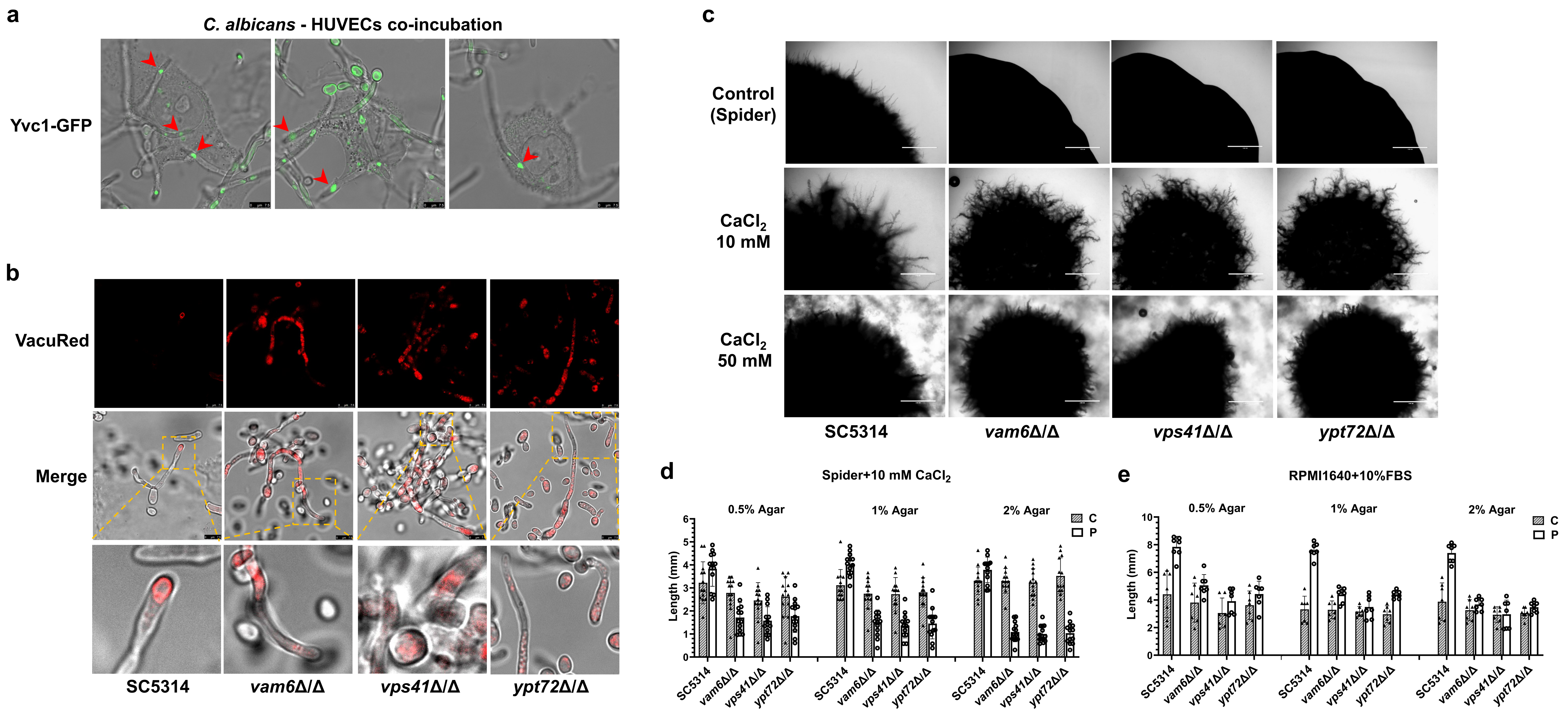

Supplementary Fig. 6

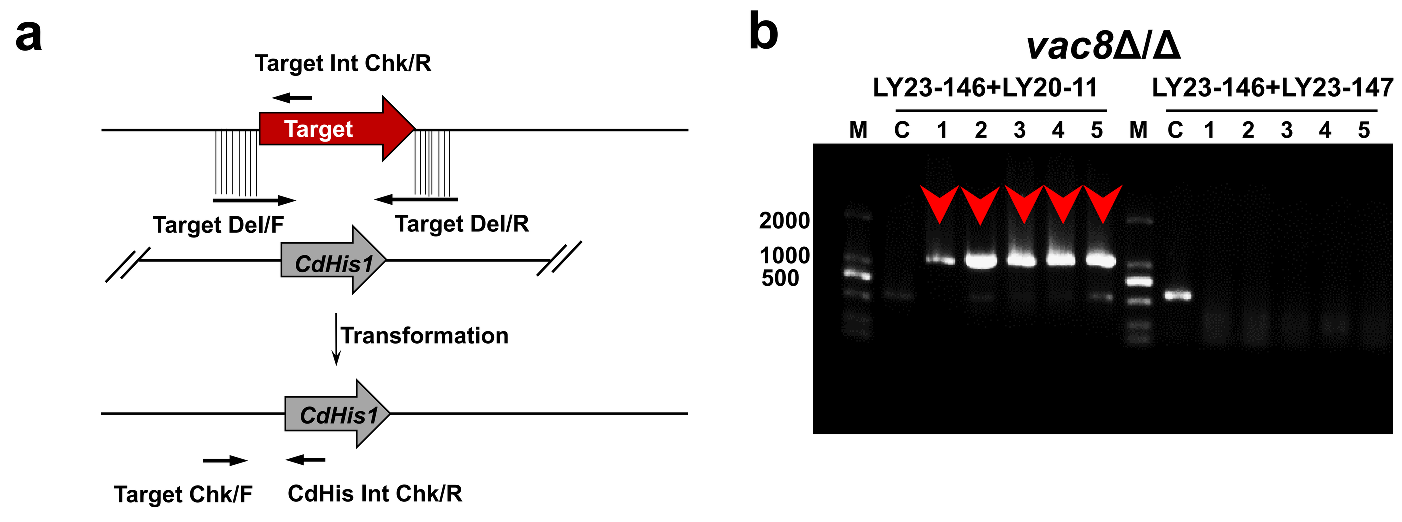

Supplementary Fig. 7

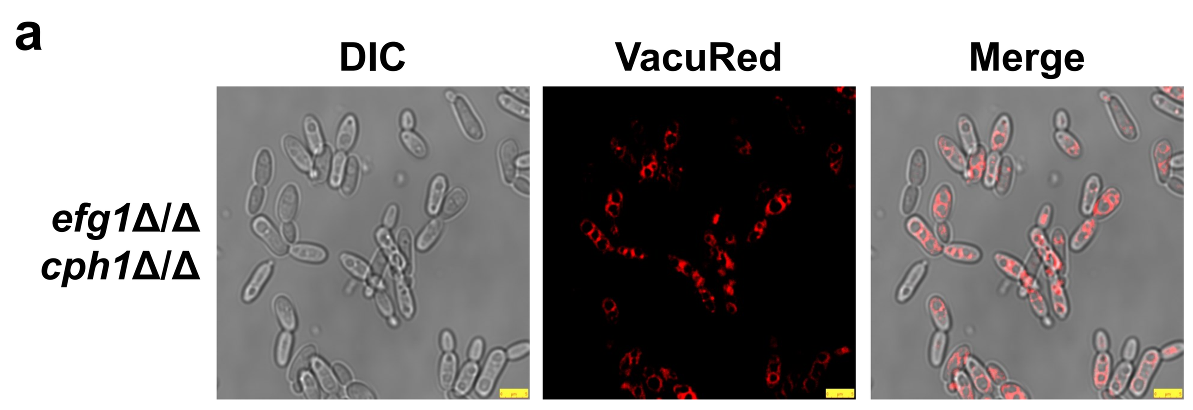

Supplement: Supplementary file 1 — Supplementary Information [file 41467_2024_48525_MOESM1_ESM.pdf]
