# Supplementary material for: The vacuolar fusion regulated by HOPS complex promotes hyphal initiation and penetration in Candida albicans
Source: Nat Commun. 2024 May 16;15:4131. doi: 10.1038/s41467-024-48525-5 (PMC11099166; doi:10.1038/s41467-024-48525-5)
Supplement: Supplementary file 3 — Description of Additional Supplementary Files [file 41467_2024_48525_MOESM3_ESM.pdf]

Supplementary Video 1. The fusion process of vacuoles during hyphal growth in GFP-tagged Yvc1 strains with VacuRed. The Yvc1-GFP strains were cultured in RPMI 1640 + 10% FBS medium at 37 °C for 90 min, and stained with 500 nM VacuRed at 37 °C for 10 min. The vacuoles were observed every 30 s. A 10 min time period is shown. The fluorescence of GFP was excited by a 488 nm laser and collected at 507 nm. Videos were exported using LAS AF Lite (2.6.1_7314). Scale bar = 2.5 μm.

Supplementary Video 2. The fusion process of vacuoles during hyphal growth in GFP-tagged Yvc1 strains with VacuRed. The Yvc1-GFP strains were cultured in RPMI 1640 + 10% FBS medium at 37 °C for 90 min, and stained with 500 nM VacuRed at 37 °C for 10 min. The vacuoles were observed every 30 s. A 10 min time period is shown. Fluorescence of VacuRed was excited by a 545 nm laser and collected between 590 nm and 670 nm. The fluorescence of GFP was excited by a 488 nm laser and collected at 507 nm. Videos were exported using LAS AF Lite (2.6.1_7314). Scale bar = 2.5 μm.

Supplementary Video 3. No obvious fusion process of vacuoles during hyphal growth in GFP-tagged Yvc1 *vam6*Δ/Δ strains with VacuRed. The Yvc1-GFP *vam6*Δ/Δ strains were cultured in RPMI 1640 + 10% FBS medium at 37 °C for 90 min, and stained with 500 nM VacuRed at 37 °C for 10 min. The vacuoles were observed every 30 s. A 10 min time period is shown. The fluorescence of GFP was excited by a 488 nm laser and collected at 507 nm. Videos were exported using LAS AF Lite (2.6.1_7314). Scale bar = 2.5 μm.

Supplementary Video 4. No obvious fusion process of vacuoles during hyphal growth in GFP-tagged Yvc1 *vam6*Δ/Δ strains with VacuRed. The Yvc1-GFP *vam6*Δ/Δ strains were cultured in RPMI 1640 + 10% FBS medium at 37 °C for 90 min, and stained with 500 nM VacuRed at 37 °C for 10 min. The vacuoles were observed every 30 s. A 10 min time period is shown. Fluorescence of VacuRed was excited by a 545 nm laser and collected between 590 nm and 670 nm. The fluorescence of GFP was excited by a 488 nm laser and collected at 507 nm. Videos were exported using LAS AF Lite (2.6.1_7314). Scale bar = 2.5 μm.

Supplementary Video 5. No obvious fusion process of vacuoles during hyphal growth in *vam6*Δ/Δstrains. The *C. albicans* strains were cultured in RPMI 1640 + 10% FBS medium at 37 ℃ for 90 min, and stained with 500 nM VacuRed at 37 ℃ for 10 min. The vacuoles were observed every 30 s. A 10 min time period is shown. Fluorescence of VacuRed was excited by a 545 nm laser and collected between 590 nm and 670 nm. Videos were exported using LAS AF Lite (2.6.1_7314). Scale bar = 7.5 μm.

Supplementary Video 6. No obvious fusion process of vacuoles during hyphal growth in *vps41*Δ/Δstrains. The *C. albicans* strains were cultured in RPMI 1640 + 10% FBS medium at 37 ℃ for 90 min, and stained with 500 nM VacuRed at 37 ℃ for 10 min. The vacuoles were observed every 30 s. A 10 min time period is shown. Fluorescence of VacuRed was excited by a 545 nm laser and collected between 590 nm and 670 nm. Videos were exported using LAS AF Lite (2.6.1_7314). Scale bar = 7.5 μm.

Supplementary Video 7. No obvious fusion process of vacuoles during hyphal growth in *ypt72*Δ/Δ strains. The *C. albicans* strains were cultured in RPMI 1640 + 10% FBS medium at 37 ℃ for 90 min, and stained with 500 nM VacuRed at 37 ℃ for 10 min. The vacuoles were observed every 30 s. A 10 min time period is shown. Fluorescence of VacuRed was excited by a 545 nm laser and collected between 590 nm and 670 nm. Videos were exported using LAS AF Lite (2.6.1_7314). Scale bar = 7.5 μm.
