# Supplementary material for: The vacuolar fusion regulated by HOPS complex promotes hyphal initiation and penetration in Candida albicans
Source: Nat Commun. 2024 May 16;15:4131. doi: 10.1038/s41467-024-48525-5 (PMC11099166; doi:10.1038/s41467-024-48525-5)
Supplement: Supplementary file 11 — Reporting Summary [file 41467_2024_48525_MOESM11_ESM.pdf]

nature portfolio

Corresponding author(s): Lan Yan

Last updated by author(s): Apr 22, 2024

Reporting Summary

Nature Portfolio wishes to improve the reproducibility of the work that we publish. This form provides structure for consistency and transparency in reporting. For further information on Nature Portfolio policies, see our Editorial Policies and the Editorial Policy Checklist.

Statistics

For all statistical analyses, confirm that the following items are present in the figure legend, table legend, main text, or Methods section.

n/a	Confirmed	
☐	☒	The exact sample size (*n*) for each experimental group/condition, given as a discrete number and unit of measurement
☐	☒	A statement on whether measurements were taken from distinct samples or whether the same sample was measured repeatedly
☐	☒	The statistical test(s) used AND whether they are one- or two-sided<br>*Only common tests should be described solely by name; describe more complex techniques in the Methods section.*
☐	☒	A description of all covariates tested
☐	☒	A description of any assumptions or corrections, such as tests of normality and adjustment for multiple comparisons
☐	☒	A full description of the statistical parameters including central tendency (e.g. means) or other basic estimates (e.g. regression coefficient) AND variation (e.g. standard deviation) or associated estimates of uncertainty (e.g. confidence intervals)
☐	☒	For null hypothesis testing, the test statistic (e.g. *F*, *t*, *r*) with confidence intervals, effect sizes, degrees of freedom and *P* value noted<br>*Give P values as exact values whenever suitable.*
☒	☐	For Bayesian analysis, information on the choice of priors and Markov chain Monte Carlo settings
☒	☐	For hierarchical and complex designs, identification of the appropriate level for tests and full reporting of outcomes
☒	☐	Estimates of effect sizes (e.g. Cohen's *d*, Pearson's *r*), indicating how they were calculated

Our web collection on statistics for biologists contains articles on many of the points above.

Software and code

Policy information about availability of computer code

Data collection	LAS X 4.6.1.27508, Tannon ABL X5 with the AllDoc_X, Tanon-2500B with the AllDoc_x
Data analysis	Image J 1.53e for image analysis, Leica LAS AF Lite 3.3.0_10134 for image analysis, Leica LAS AF Lite 2.6.1_7314 for image analysis and video export, Graphpad Prism version 9.0.0 (121), Graphpad Prism version 8.3.0 (538) for data and statistical analysis, SPSS Statistics 24 for statistical analysis, and Microsoft office 2020 for image and data analysis.

For manuscripts utilizing custom algorithms or software that are central to the research but not yet described in published literature, software must be made available to editors and reviewers. We strongly encourage code deposition in a community repository (e.g. GitHub). See the Nature Portfolio guidelines for submitting code & software for further information.

Data

Policy information about availability of data

All manuscripts must include a data availability statement. This statement should provide the following information, where applicable:

- Accession codes, unique identifiers, or web links for publicly available datasets
- A description of any restrictions on data availability
- For clinical datasets or third party data, please ensure that the statement adheres to our policy

The data supporting the findings from this study are available within the article file, supplementary information, and source data file. Any other raw data in this study are available from the corresponding author upon request. Source data are provided as a Source Data file.

Research involving human participants, their data, or biological material

Policy information about studies with human participants or human data. See also policy information about sex, gender (identity/presentation), and sexual orientation and race, ethnicity and racism.

Reporting on sex and gender	NA
Reporting on race, ethnicity, or other socially relevant groupings	NA
Population characteristics	NA
Recruitment	NA
Ethics oversight	NA

Note that full information on the approval of the study protocol must also be provided in the manuscript.

Field-specific reporting

Please select the one below that is the best fit for your research. If you are not sure, read the appropriate sections before making your selection.

☒ Life sciences ☐ Behavioural & social sciences ☐ Ecological, evolutionary & environmental sciences

For a reference copy of the document with all sections, see nature.com/documents/nr-reporting-summary-flat.pdf

Life sciences study design

All studies must disclose on these points even when the disclosure is negative.

Sample size	No sample-size calculation was performed. Sample sizes were estimated based on standards of the field and preliminary experiments. For in vitro experiment, one fungal strain sample means one colony from the 48 h-cultured Candida albicans strains on the solid medium. Such colony was overnight cultured in the liquid medium and usually reached to 10^8 cells/ml. 3-50 samples was used for analysis. For hyphal-length measurement, 20-50 hyphal cells were used in different microscopy fields for each fungal strain. For in vivo experiment, each group contains 5-10 mice. These sample sizes were sufficient to detect biological difference with good reproducibility. All the sample size were clearly provided in our figure legends and methods.
Data exclusions	No data was excluded from the analyses.
Replication	Each experiment was replicated at least three times and all attempts at replicating assay were successful.
Randomization	In the in vivo experiments, mice were randomly allocated to different experimental groups and inoculated with the wild type and the mutant C.albicans. In the HUVEC or macrophages co-cultured with C. albicans assay, HUVECs or macrophages were randomly allocated to different experimental groups and cultured with the wild type and the mutant C. albicans.
Blinding	Investigators were blinded to group allocation during data collection and analysis.

Reporting for specific materials, systems and methods

We require information from authors about some types of materials, experimental systems and methods used in many studies. Here, indicate whether each material, system or method listed is relevant to your study. If you are not sure if a list item applies to your research, read the appropriate section before selecting a response.

Materials & experimental systems

n/a	Involved in the study	
☐	☒	Antibodies
☐	☒	Eukaryotic cell lines
☒	☐	Palaeontology and archaeology
☐	☒	Animals and other organisms
☒	☐	Clinical data
☒	☐	Dual use research of concern
☒	☐	Plants

Methods

n/a	Involved in the study	
☒	☐	ChIP-seq
☒	☐	Flow cytometry
☒	☐	MRI-based neuroimaging

Antibodies

Antibodies used	Anti-phospho (S/T) Akt substrate rabbit polyclonal antibody, Cell Signaling Technology, catalog # 9611; 1: 1000 for WB;<br>Total S6 (anti-S6 sheep polyclonal antibody (R&D, catalog # AF5436); 1: 1000 for WB;<br>Anti-histone H3 rabbit polyclonal antibody, Cell Signaling Technology, catalog # 4499; 1: 1000 for WB;<br>Peroxidass-conjugrated Affinipure goat Anti-Rabbit, Proteintech, catalog # SA00001-2; 1: 2000 for WB;<br>Peroxidass-conjugrated Affinipure Rabbit Anti-Sheep, Proteintech, catalog # SA00001-16; 1: 2000 for WB.
Validation	All antibodies used in this study are commercially available antibodies. Validations for each of these commercial antibodies are provided on the manufacturers' websites and include specificity and quality control testing via flow cytometry and testing for contaminants including endotoxin. The specific antibody information are as follows.<br>Phospho-(Ser/Thr) Akt Substrate Antibody preferentially recognizes peptides and proteins containing phospho-Ser/Thr preceded by Lys/Arg at positions -5 and -3, in a manner largely independent of other surrounding amino acids. Some cross-reactivity is observed for peptides that contain phospho-Ser/Thr preceded by Arg/Lys at positions -3 and -2. No cross-reactivity is observed with the corresponding nonphosphorylated sequences or with other phospho-Ser/Thr-containing motifs.<br>https://www.cellsignal.cn/products/primary-antibodies/phospho-ser-thr-akt-substrate-antibody/9611<br>Ribosomal protein S6 (rpS6; also Phosphoprotein NP33) is a 32-34 kDa member of the ribsosmal protein S6e family of molecules.<br>https://www.rndsystems.com/cn/products/human-mouse-rat-ribosomal-protein-s6-rps6-antibody_af5436<br>Histone H3 (D1H2) XP ® Rabbit mAb can detect endogenous levels of H3 total histones (including isotypes H3.1, H3.2, and H3.3). This antibody can also detect the histone H3 variant CENP-A. This antibody will not cross react with other core histones.<br>https://www.cellsignal.cn/products/primary-antibodies/histone-h3-d1h2-xp-rabbit-mab/4499

Eukaryotic cell lines

Policy information about cell lines and Sex and Gender in Research

Cell line source(s)	HUVEC cells; Peritoneal macrophages were collected from female ICR mice and distinguished from other cells by adhering to the well.
Authentication	HUVEC cells were obtained from the ATCC® CRL-1730.
Mycoplasma contamination	Without mycoplasma contamination
Commonly misidentified lines (See ICLAC register)	No commonly misidentified cell lines are used.

Animals and other research organisms

Policy information about studies involving animals; ARRIVE guidelines recommended for reporting animal research, and Sex and Gender in Research

Laboratory animals	Female ICR mice (6-8 weeks, 20-22 g) were purchased from Bikaikeji Biotechnology Co., LTD. Femail C57BL/6 mice (6-8 weeks, 20-22 g) were purchased from Leigen Biotechnology Co., LTD. Mice were routinely maintained in a clean room at a temperature of 18-25°C, relative humidity of 30-70% and under a constant 12-h light/dark cycle. Mice were given free access to food and water throughout the study.
Wild animals	This study did not involve wilde animals.
Reporting on sex	Female mice were mainly used for the C. albicans systemic and skin infection models in this study. Choosing female mice was mainly due to the stable results, which exhibited a low incidence of fatal biting.
Field-collected samples	This study did not involve field-collected samples.
Ethics oversight	All animal experiments were approved by the Committee on Ethics of Medicine, Naval Medical University.

Note that full information on the approval of the study protocol must also be provided in the manuscript.

Plants

Seed stocks	NA
Novel plant genotypes	NA
Authentication	NA
