# Supplementary material for: The vacuolar fusion regulated by HOPS complex promotes hyphal initiation and penetration in Candida albicans
Source: Nat Commun. 2024 May 16;15:4131. doi: 10.1038/s41467-024-48525-5 (PMC11099166; doi:10.1038/s41467-024-48525-5)

**Source data of Fig. 1b-1c. Images used to measure the length of hyphae, and to count the number of hyphae with the first and the secondary branching, in the wild type and the *vam6* null mutant.**


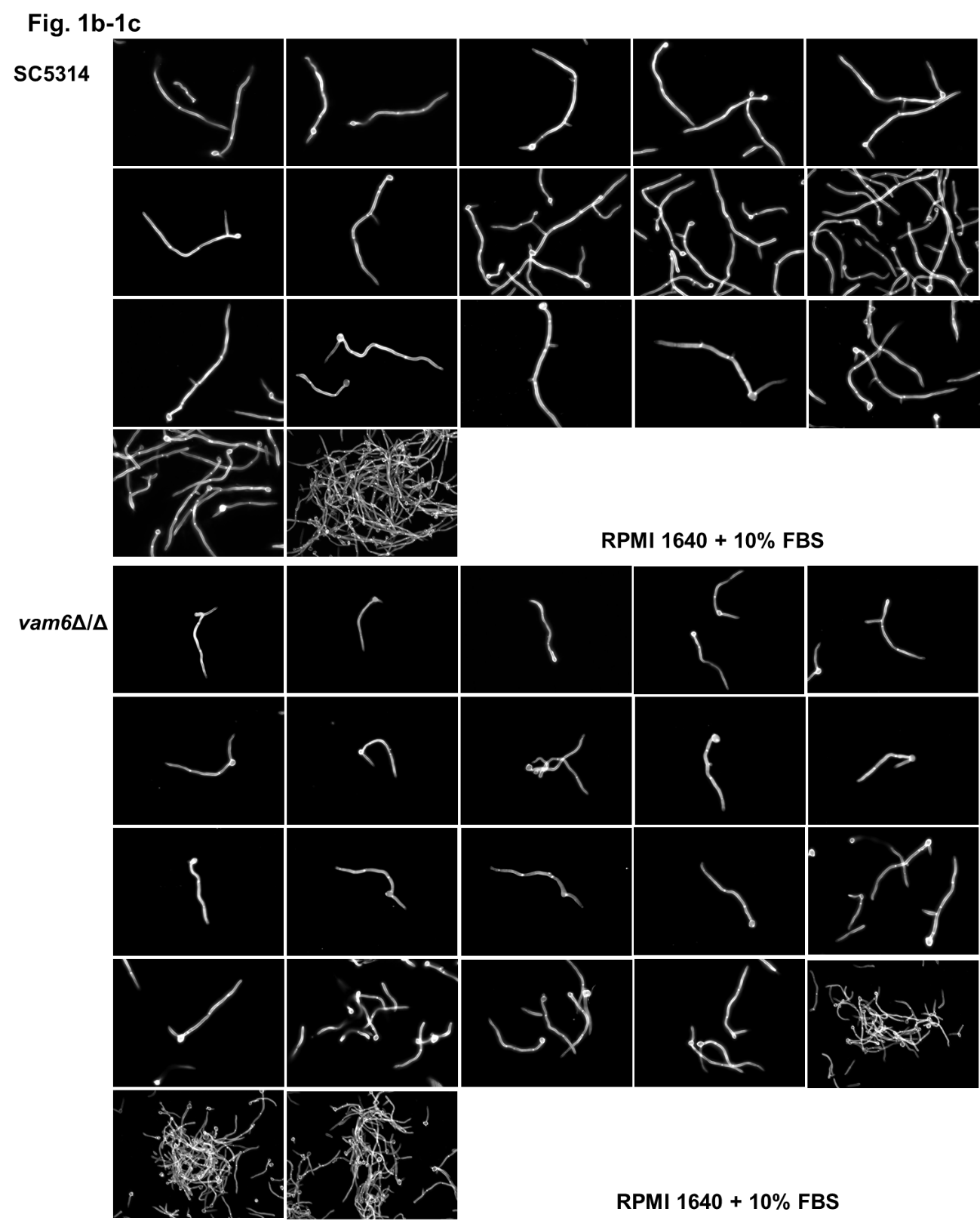


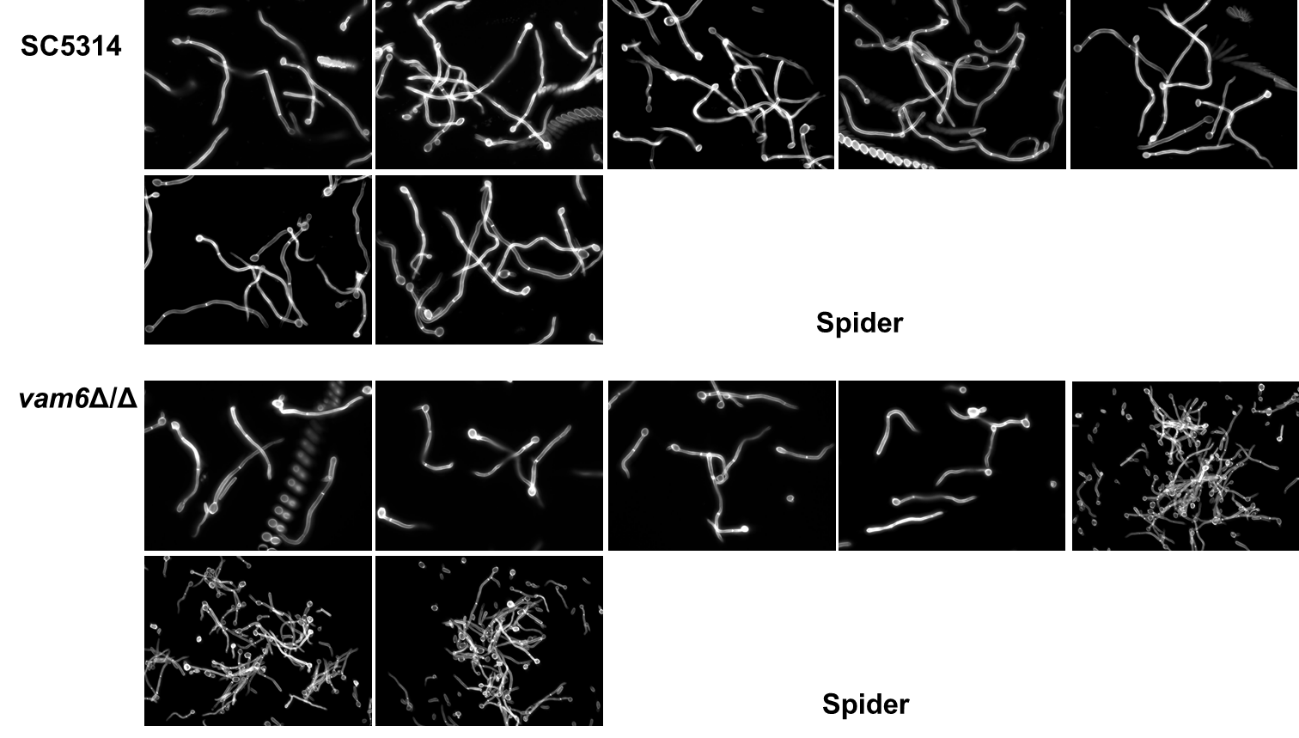

Supplement: Supplementary file 12 — Source Data [file 41467_2024_48525_MOESM12_ESM.zip › Source Data of Figure 1.docx]
