# Supplementary material for: The vacuolar fusion regulated by HOPS complex promotes hyphal initiation and penetration in Candida albicans
Source: Nat Commun. 2024 May 16;15:4131. doi: 10.1038/s41467-024-48525-5 (PMC11099166; doi:10.1038/s41467-024-48525-5)
Supplement: Supplementary file 12 — Source Data [file 41467_2024_48525_MOESM12_ESM.zip › Source Data of Figure 2.docx]

**Source data of Figure 2.**


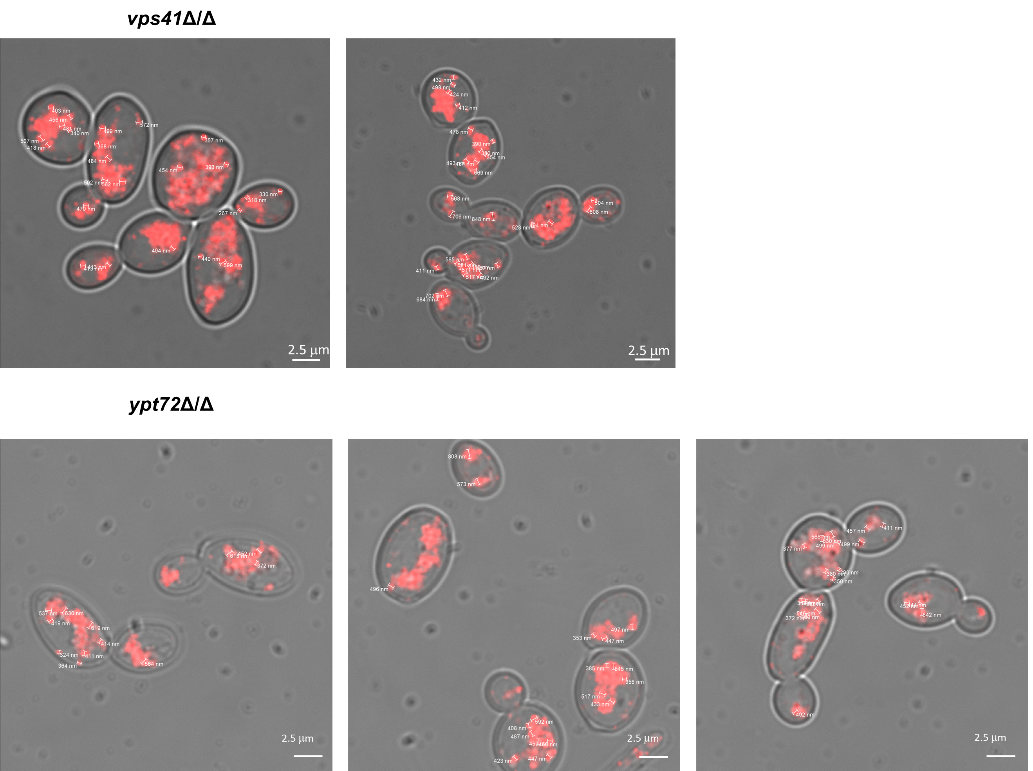
Fig. 2c. Images to measure the vacuolar diameters in *C. albicans*.


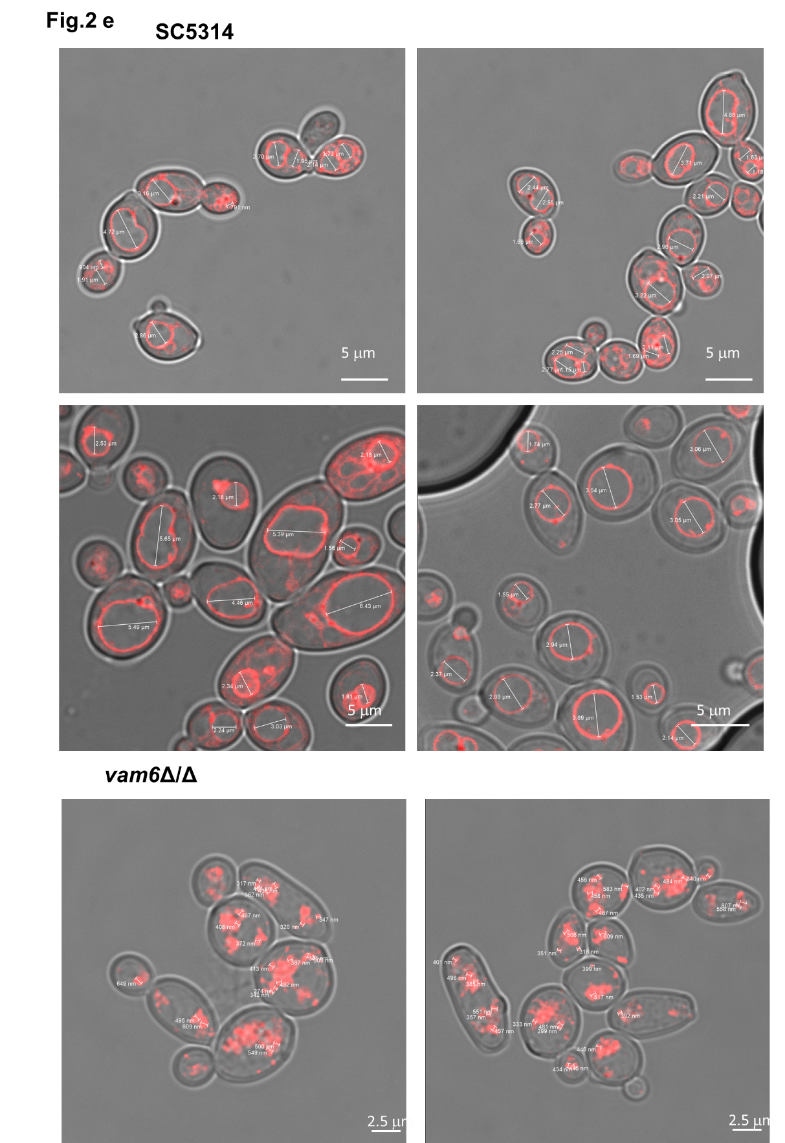

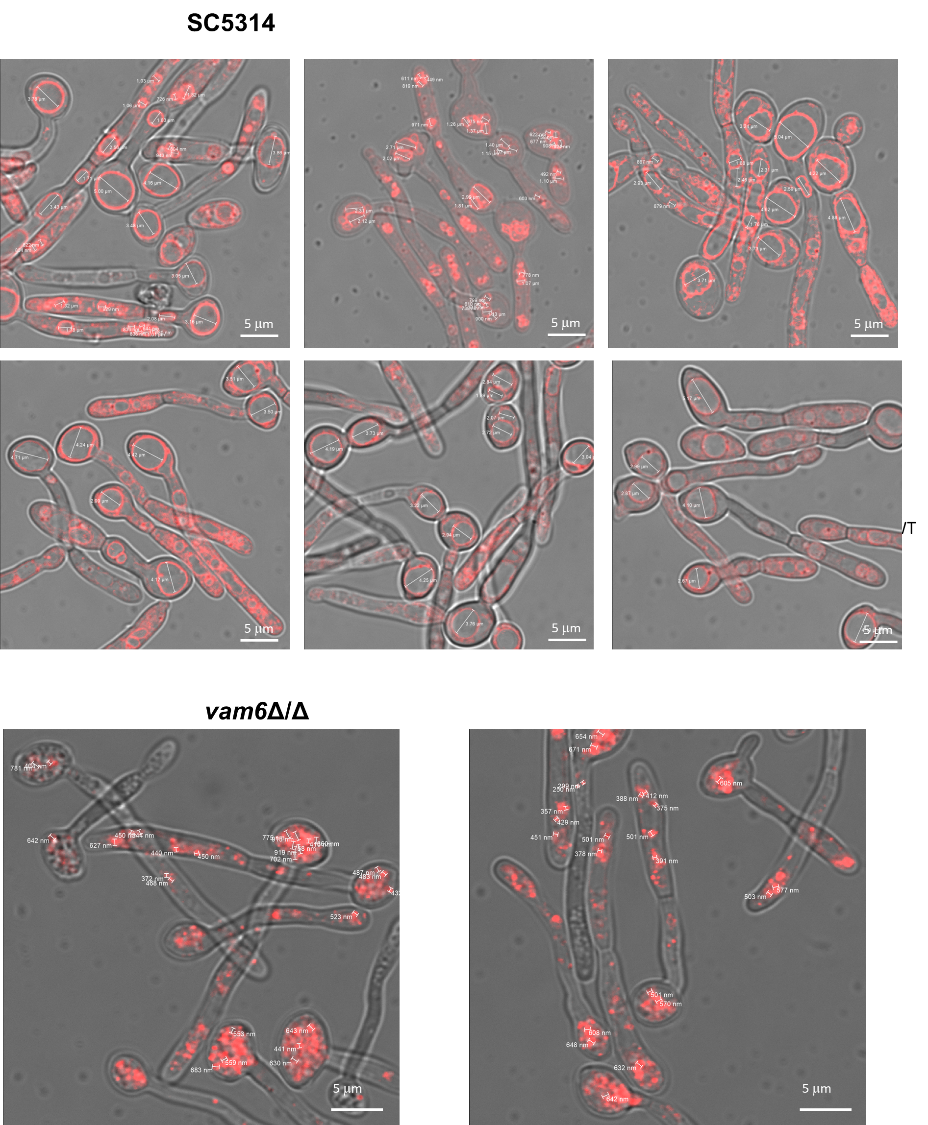


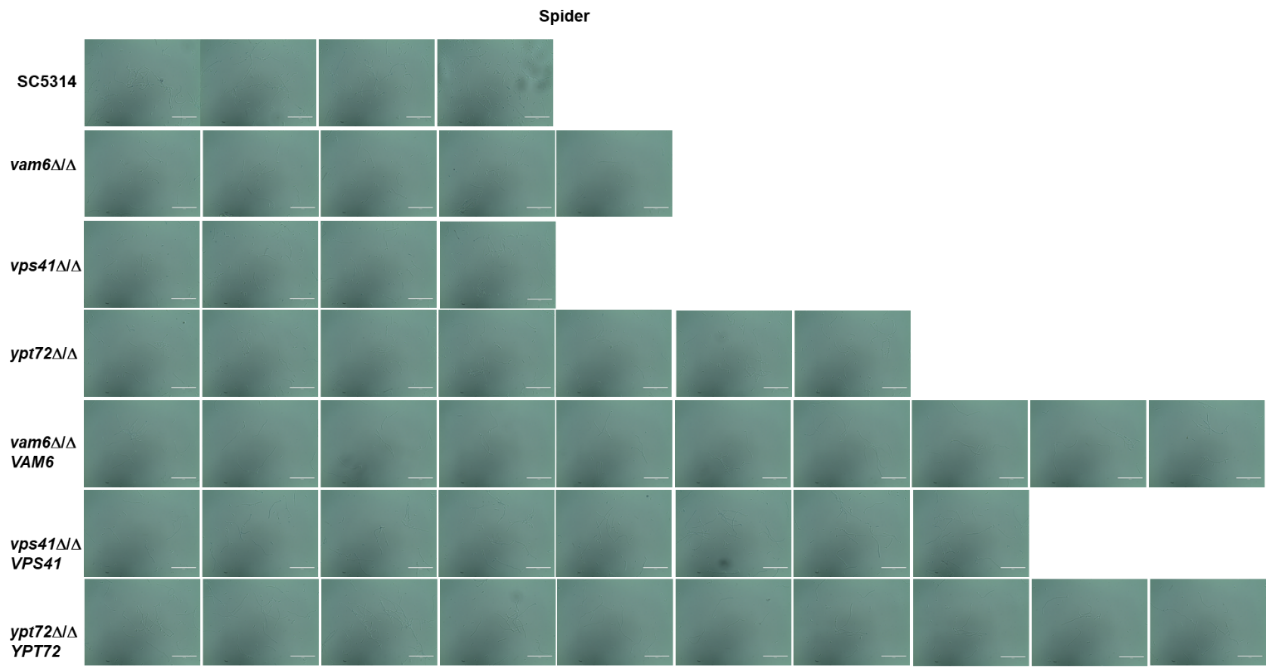

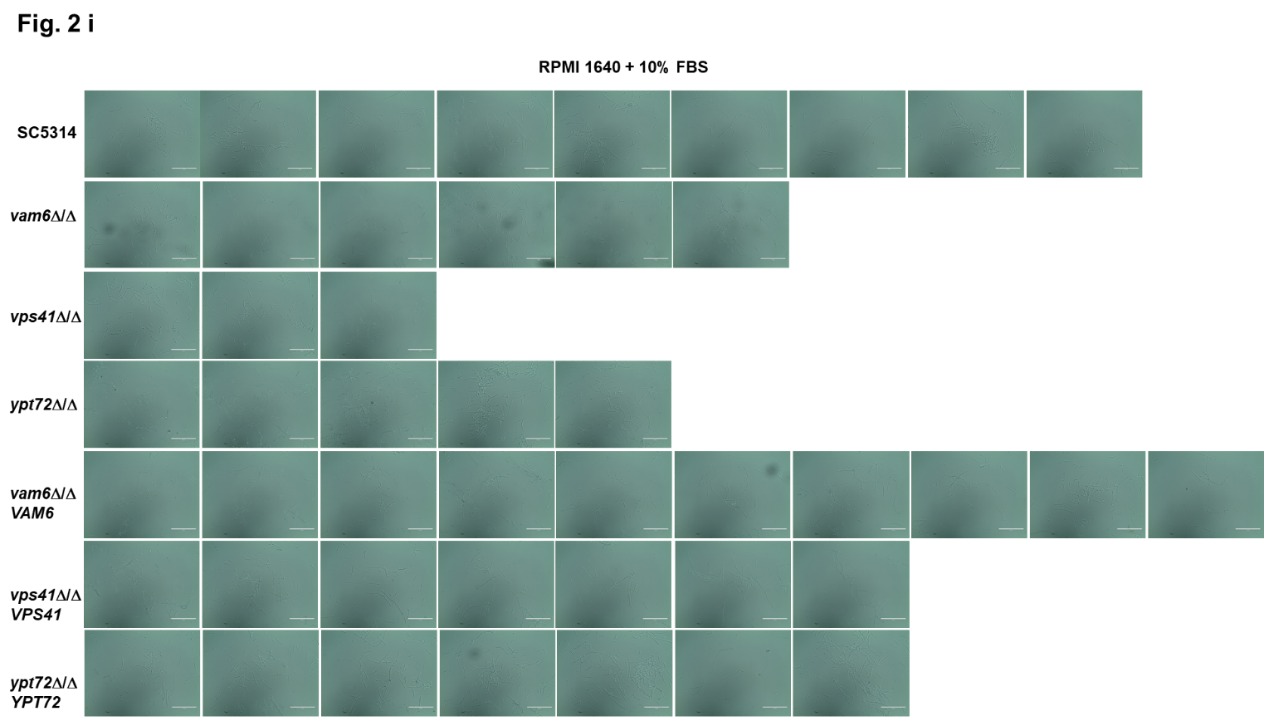
Fig. 2i and Fig. S2q. Images used to measure the length of hyphae.


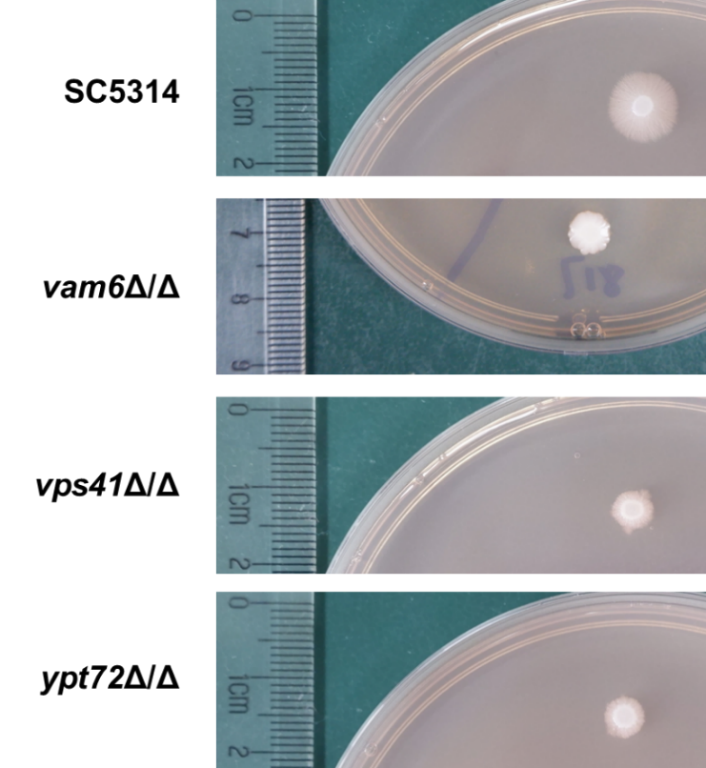

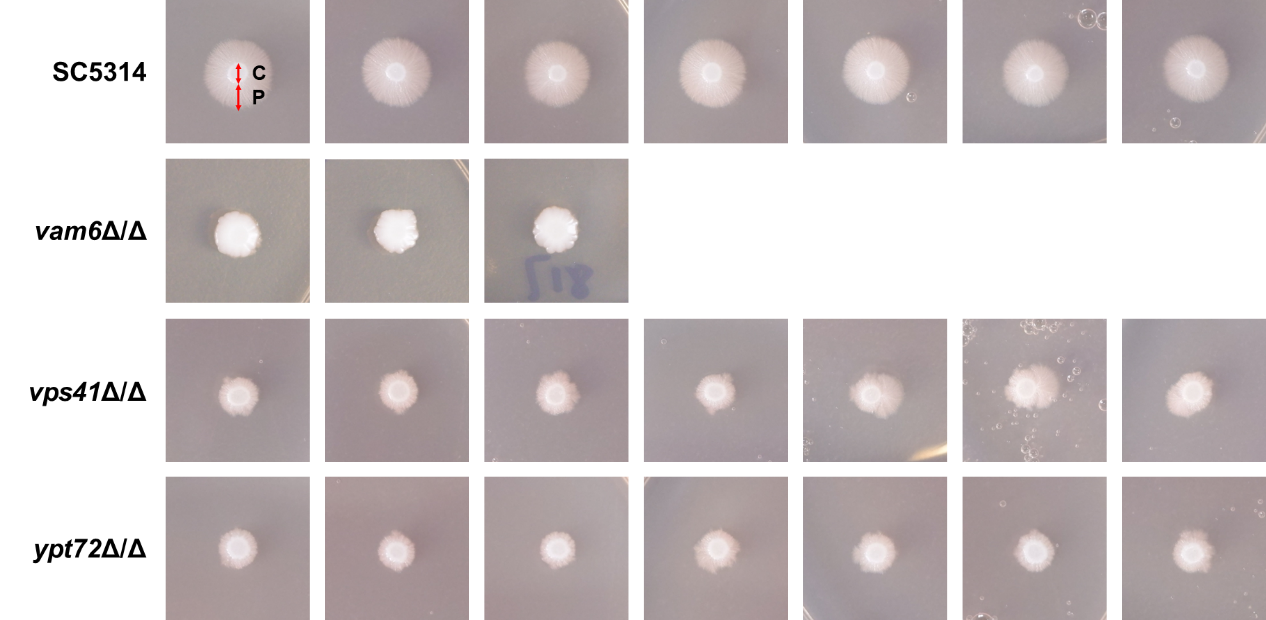

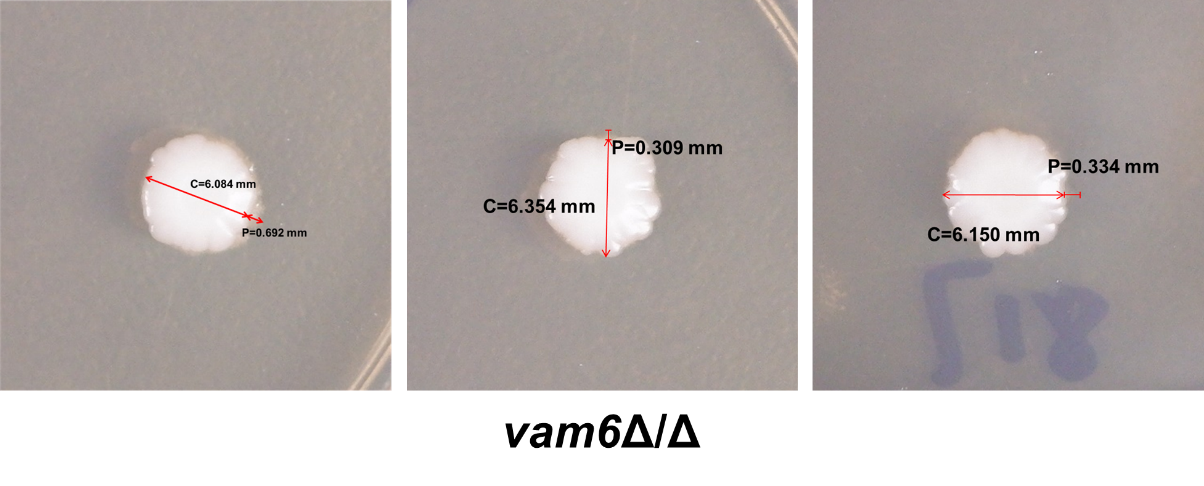
Fig. 2k. Images used to measure the C and P of hyphal colonies on solid media of RPMI1640 + 10% FBS. The images were in size of 2 cm × 2 cm, and the measurement of length was based on the ruler scale as shown.


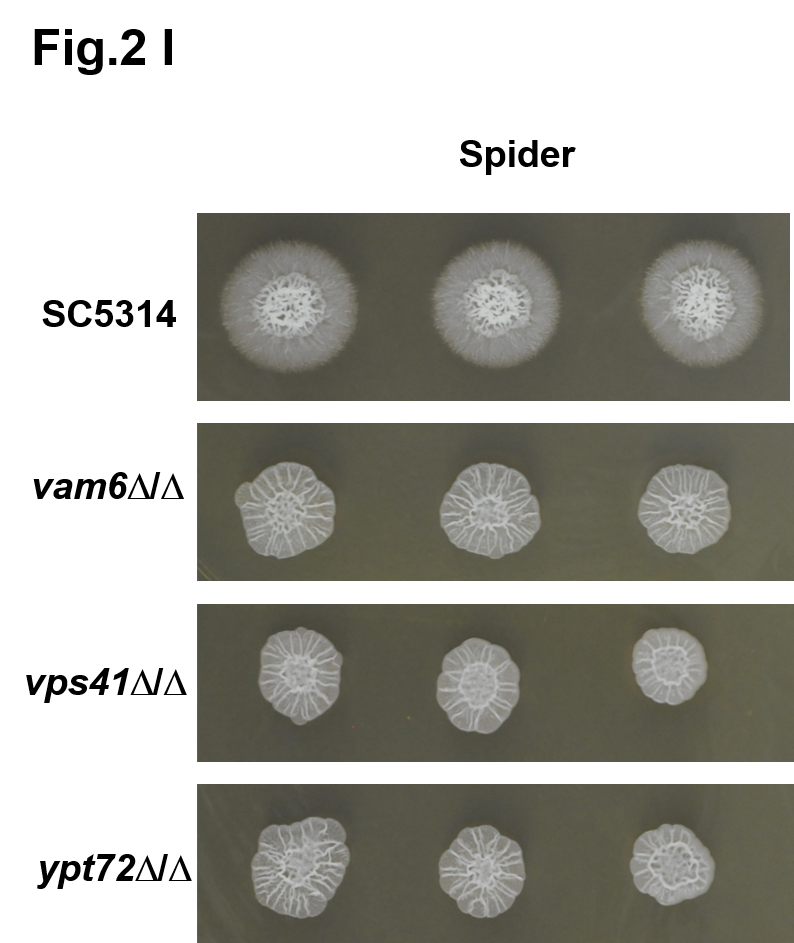
Fig. 2l. Images used to measure the C and P of hyphal colonies on solid media of Spider. The measurement of length was based on the ruler scale.


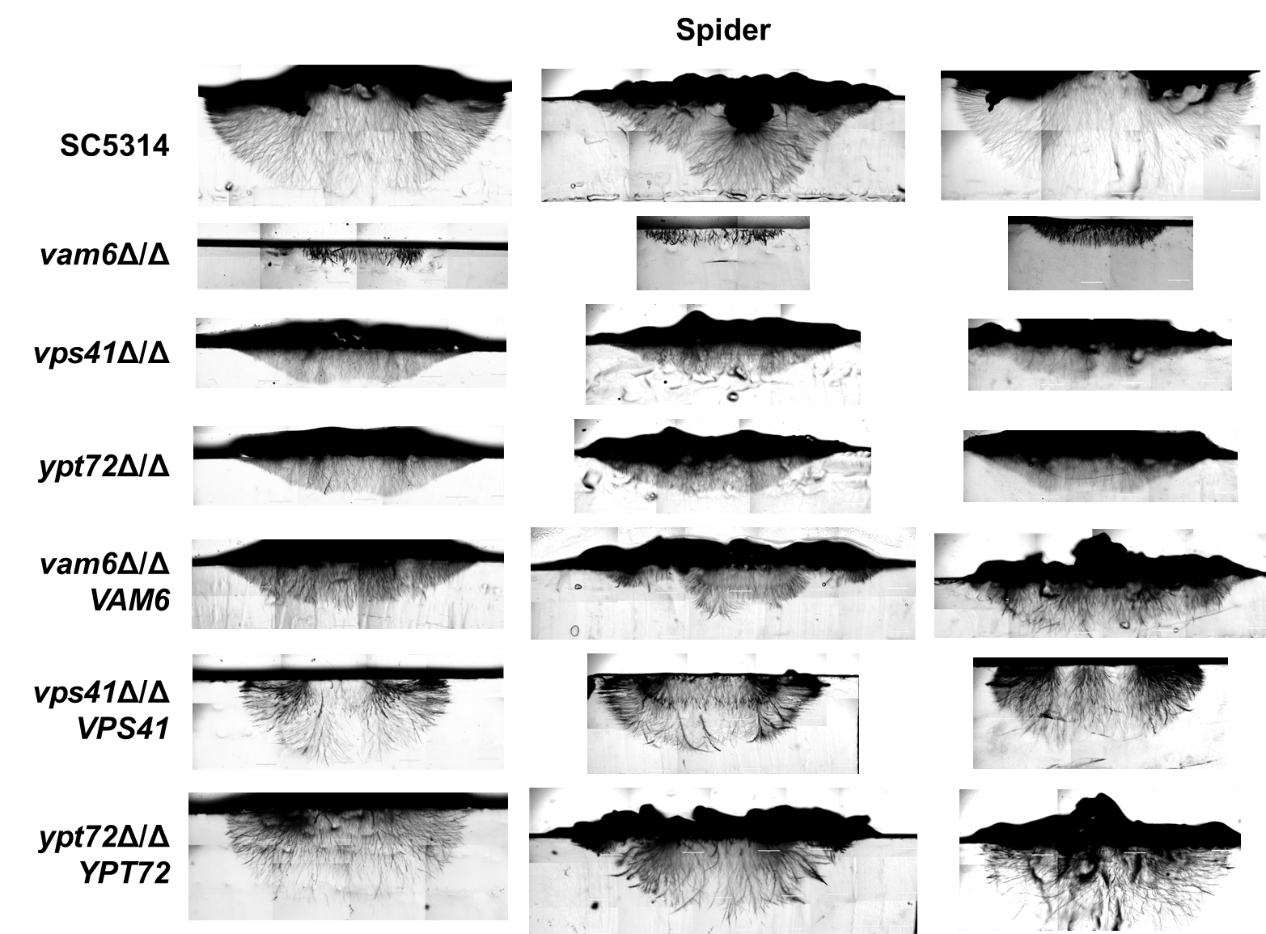

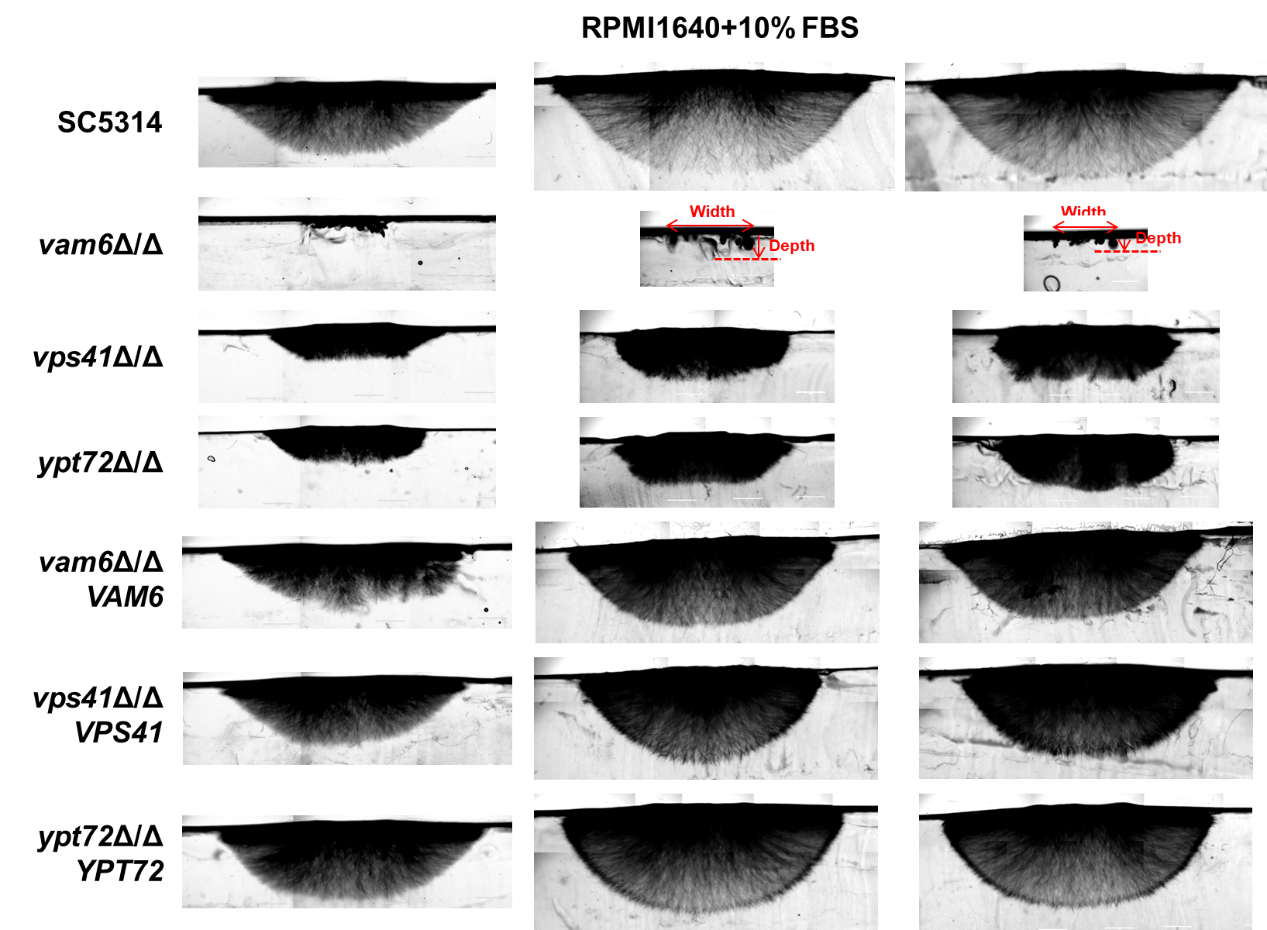
Fig 2n and Fig S2r. Images used to measure the width (W) and depth (D) of hyphal colonies on solid media. Three biological replicates of the vertical sections were observed by microscopy.
