# Supplementary material for: The vacuolar fusion regulated by HOPS complex promotes hyphal initiation and penetration in Candida albicans
Source: Nat Commun. 2024 May 16;15:4131. doi: 10.1038/s41467-024-48525-5 (PMC11099166; doi:10.1038/s41467-024-48525-5)
Supplement: Supplementary file 12 — Source Data [file 41467_2024_48525_MOESM12_ESM.zip › Source Data of Figure 3.docx]

**Source data of Figure 3.**


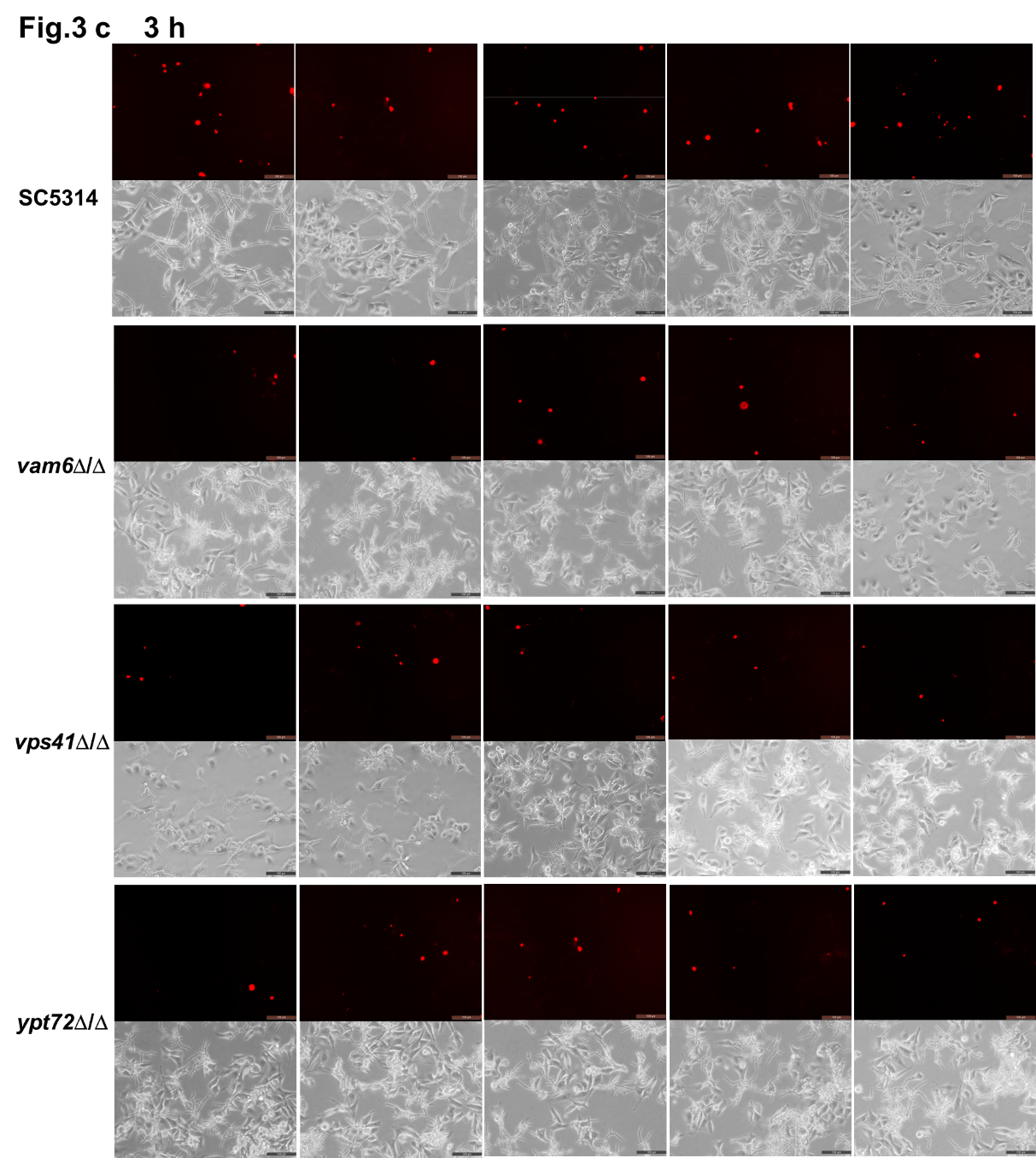
Fig. 3c. Images used to calculate the percentage of PI-positive macrophages co-incubated with *C. albicans*.


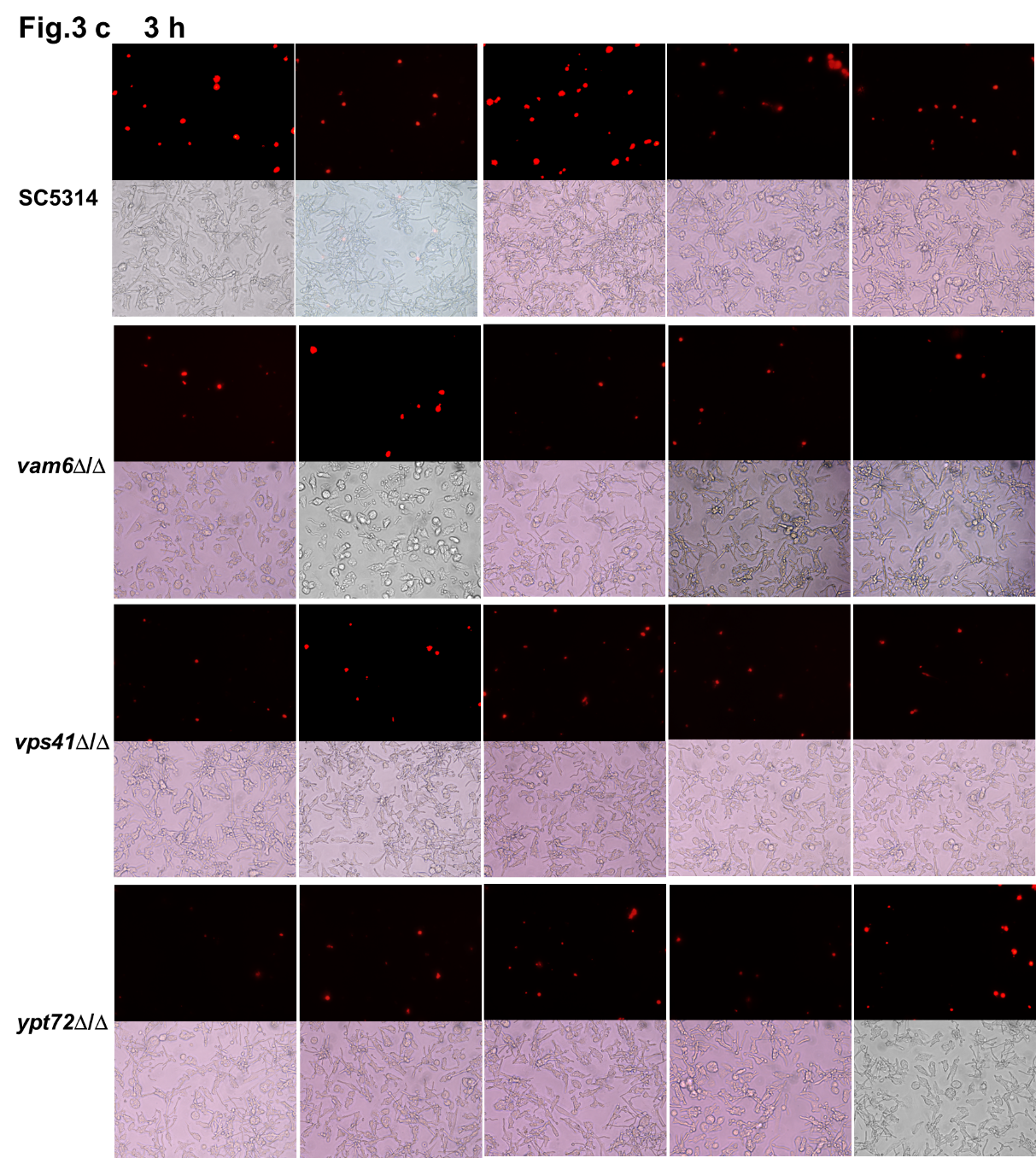


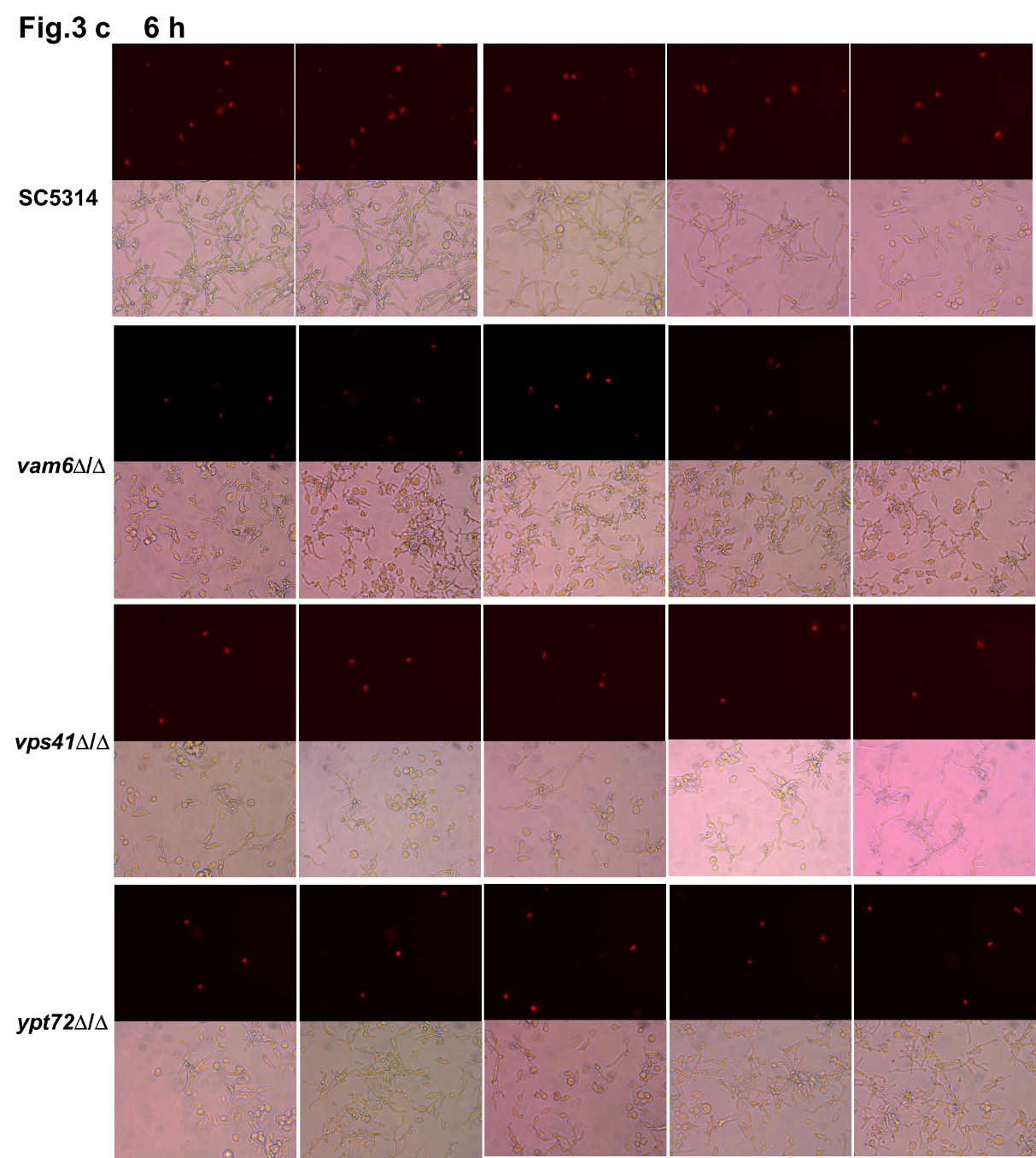


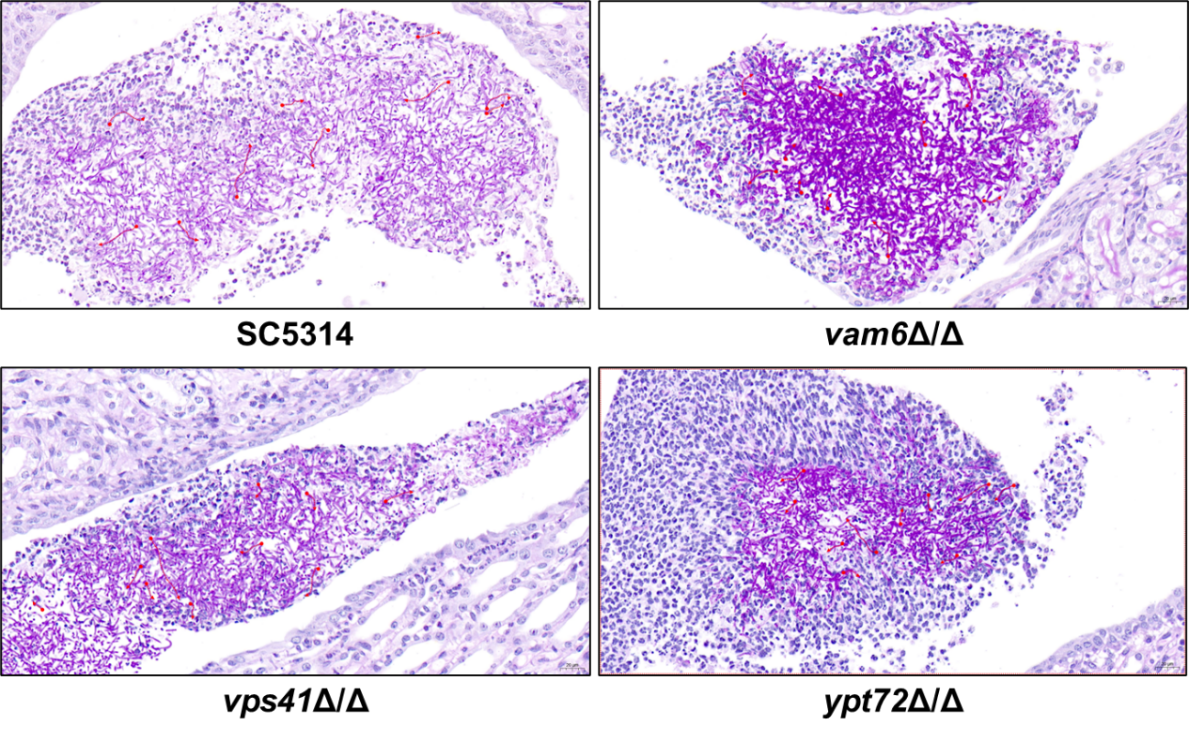
Fig. 3i. Images used to measure the length of hyphae in kidneys in mice infected with *C. albicans*. Some of the measured hyphae have been shown in red lines.


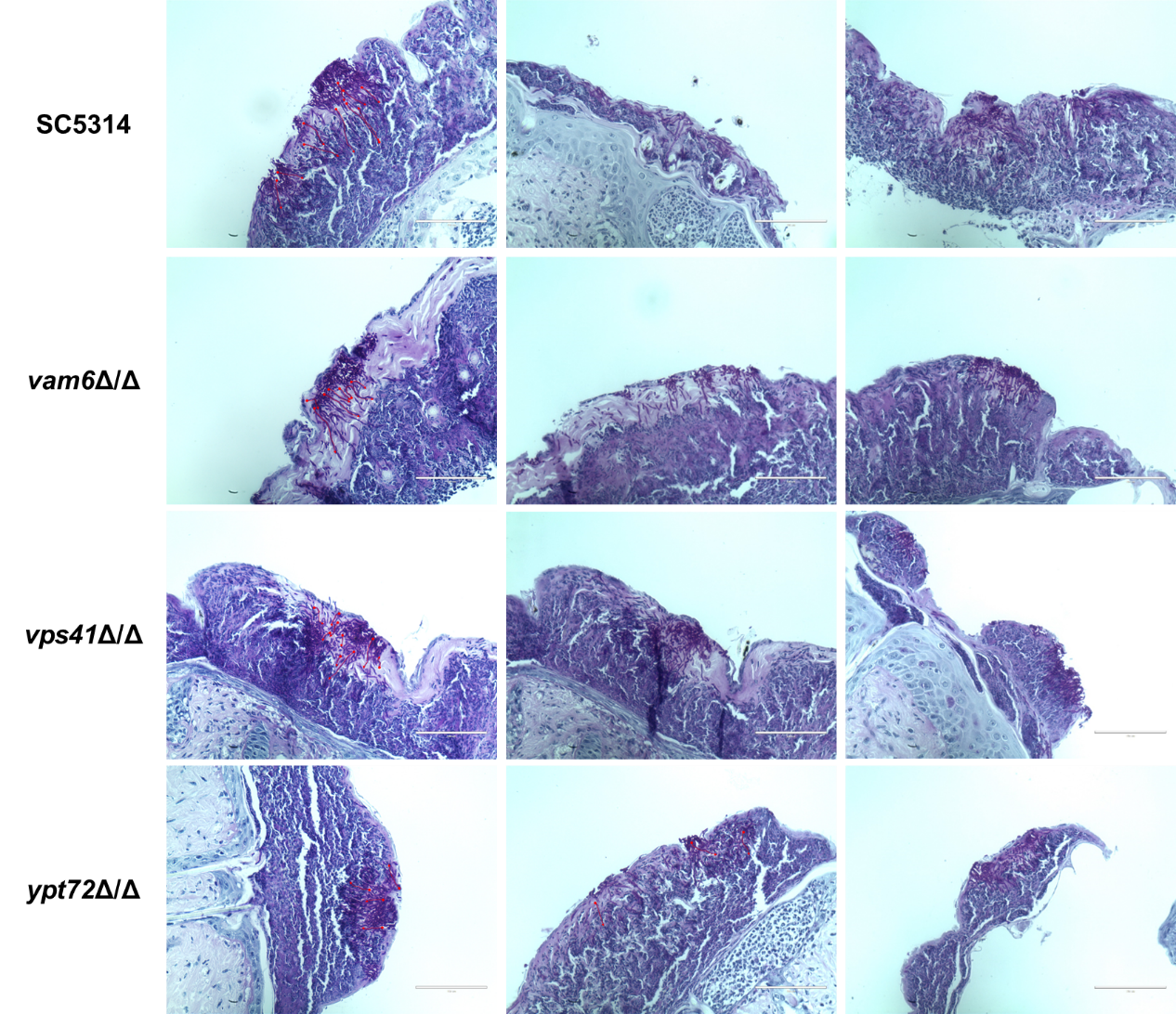
Fig. 3n. Images used to measure the length of hyphae in skin in mice infected with *C. albicans*. Some of the measured hyphae have been shown in red lines.
