# Supplementary material for: The vacuolar fusion regulated by HOPS complex promotes hyphal initiation and penetration in Candida albicans
Source: Nat Commun. 2024 May 16;15:4131. doi: 10.1038/s41467-024-48525-5 (PMC11099166; doi:10.1038/s41467-024-48525-5)
Supplement: Supplementary file 12 — Source Data [file 41467_2024_48525_MOESM12_ESM.zip › Source Data of Figure 4.docx]

**Source data of Figure 4.**


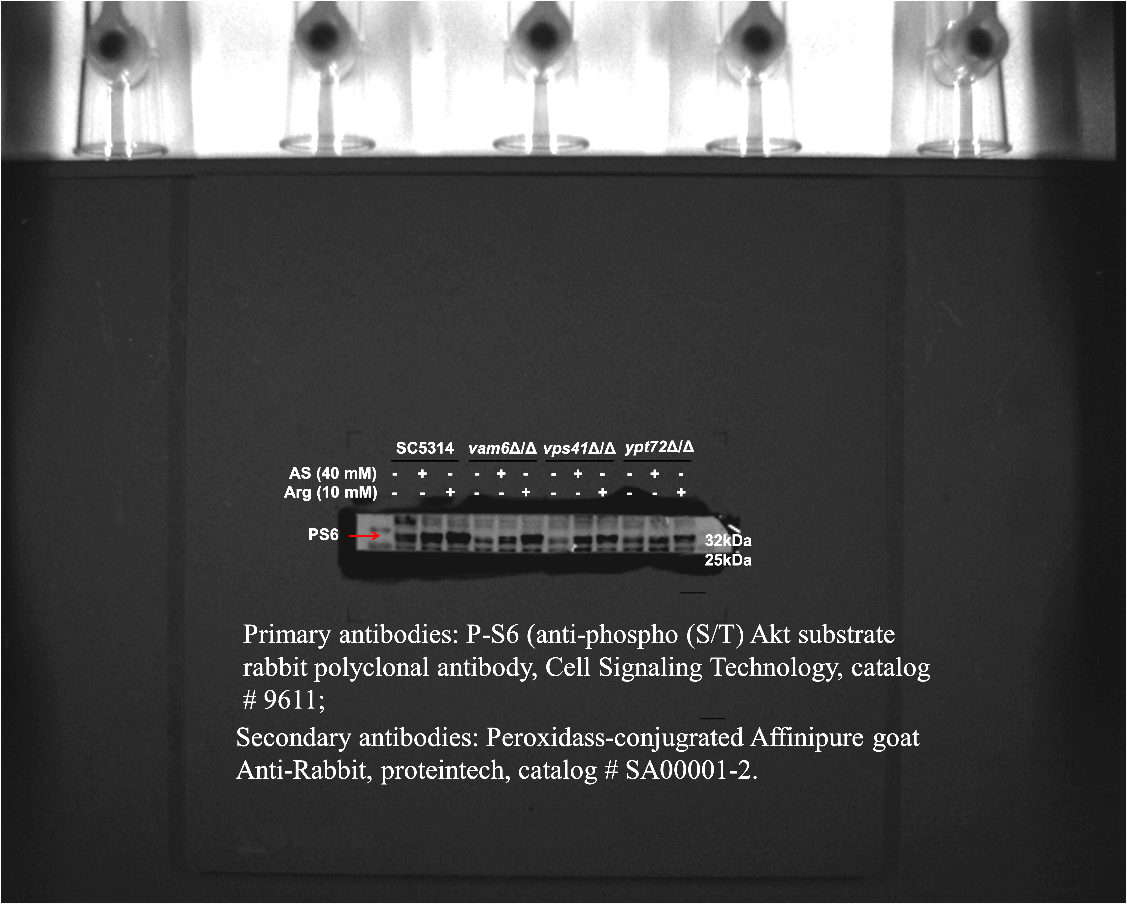
Fig. 4b. Original images of Western blots.


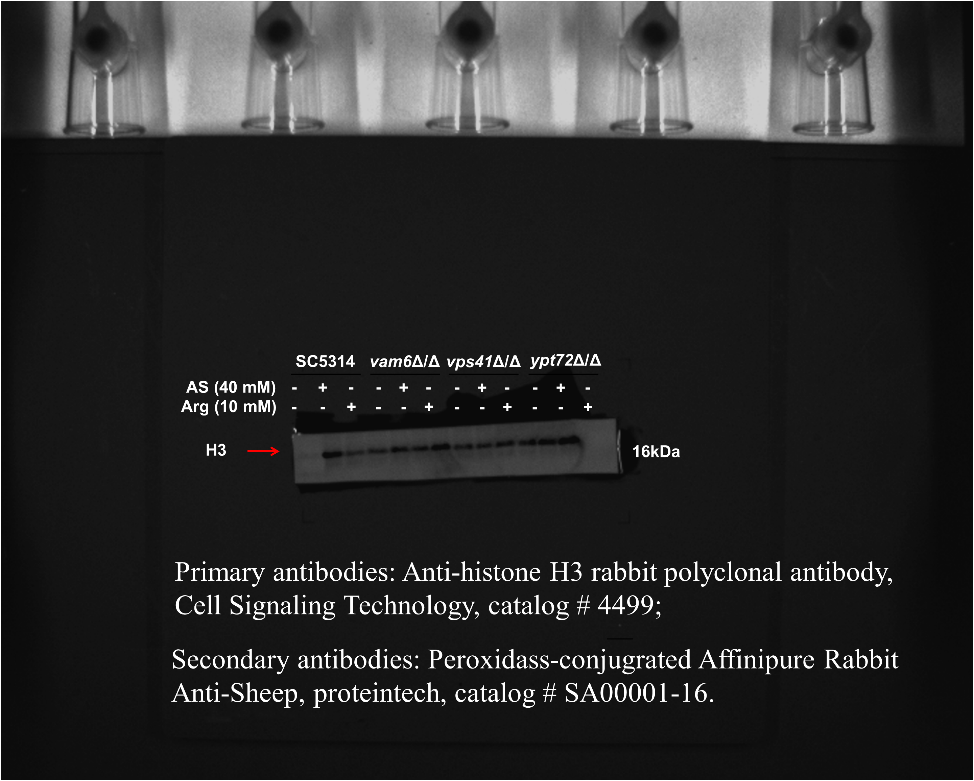

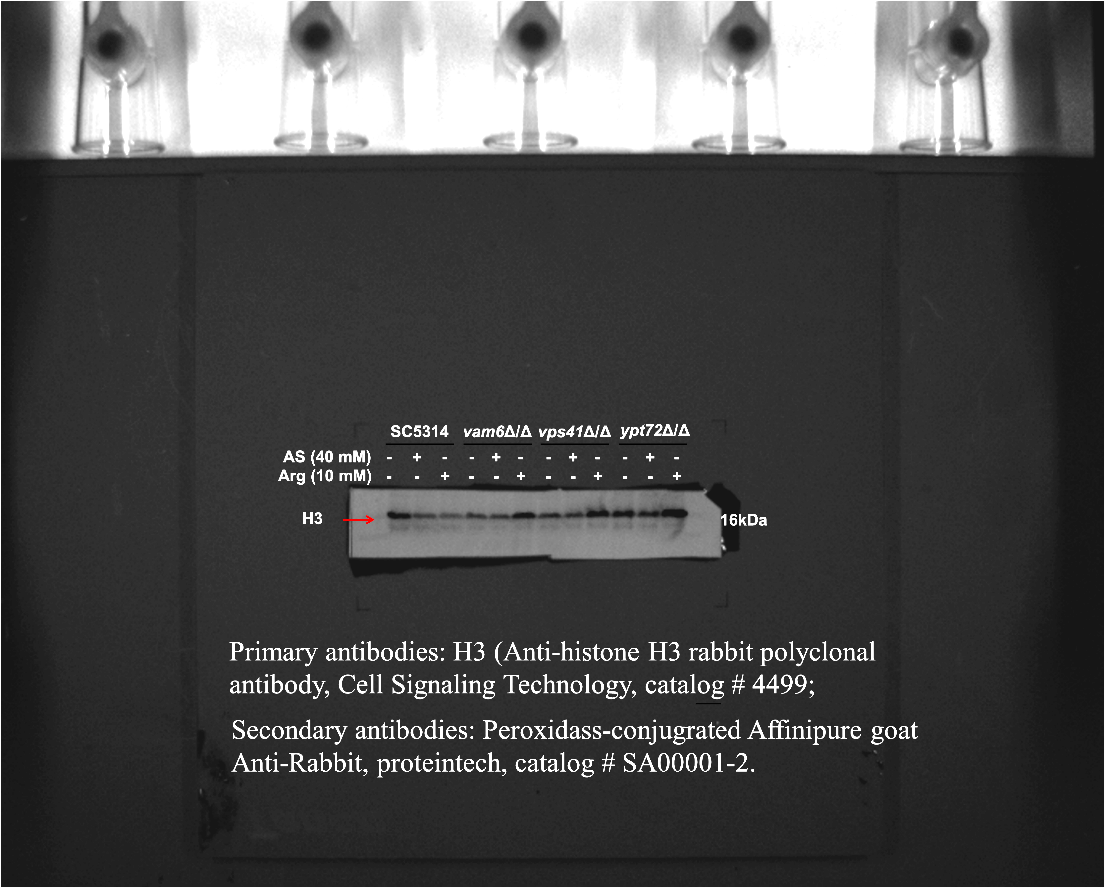

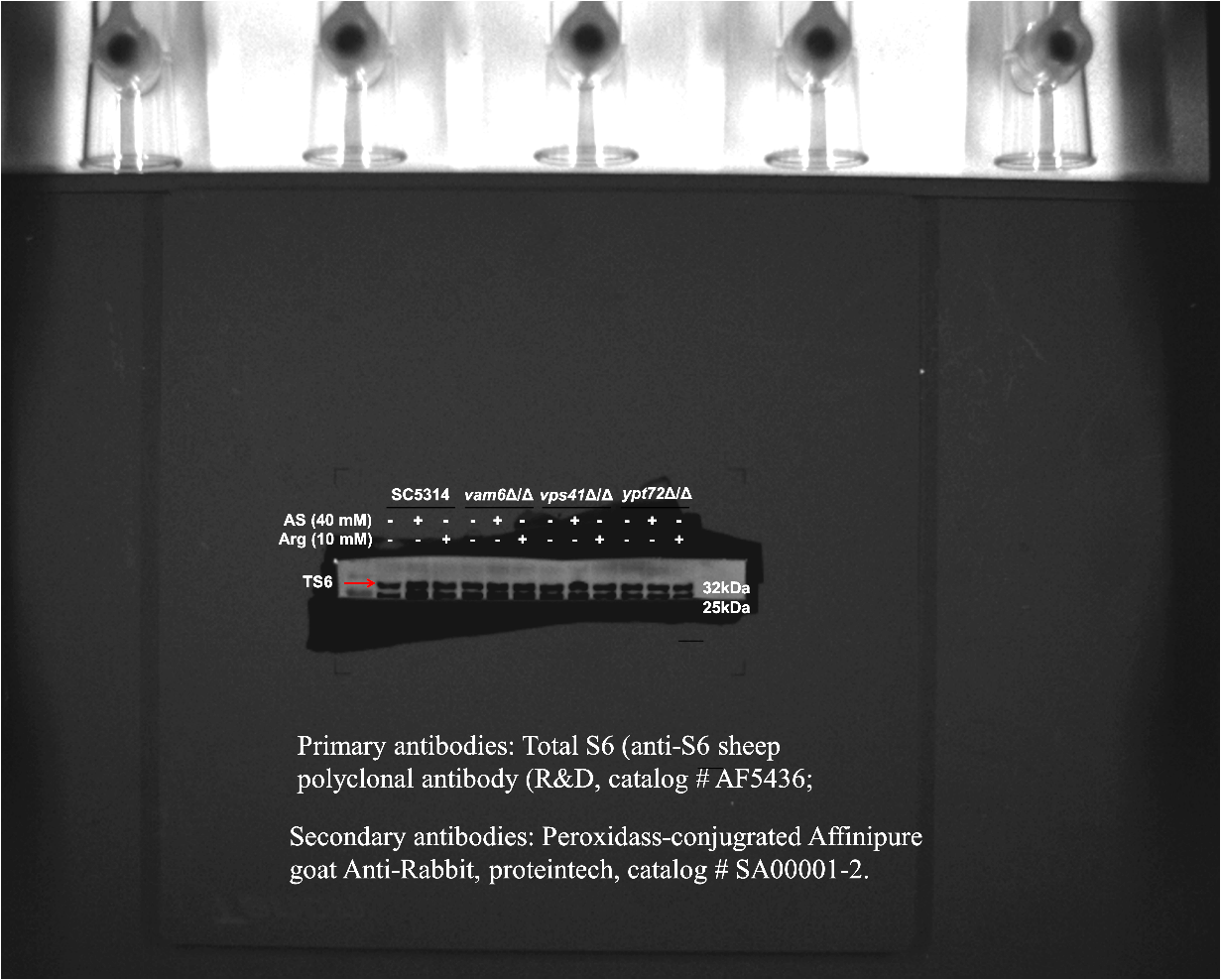
Fig. 4c. Images used to measure the width (W) and depth (D) of hyphal colonies on solid
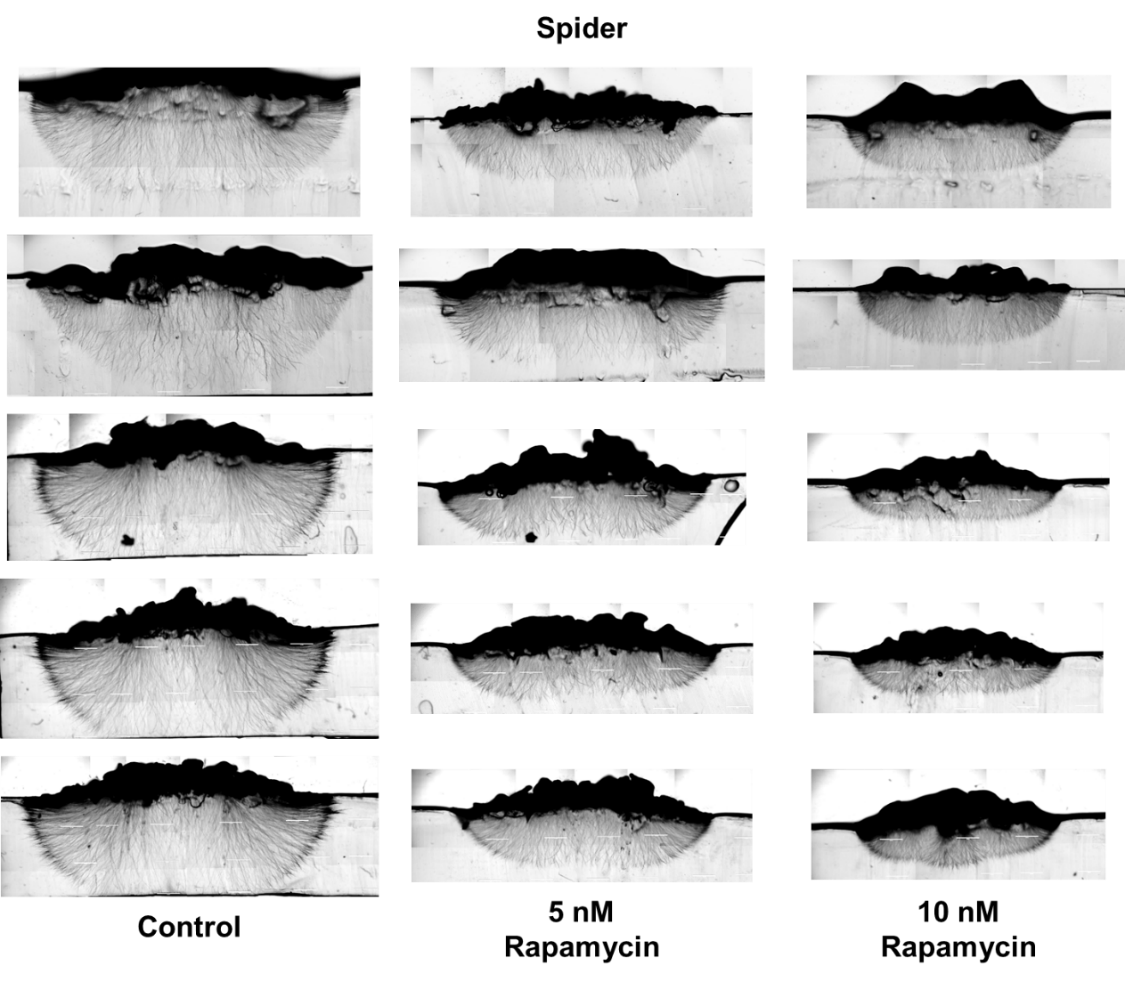
media. Five biological replicates of the vertical sections were observed by microscopy.


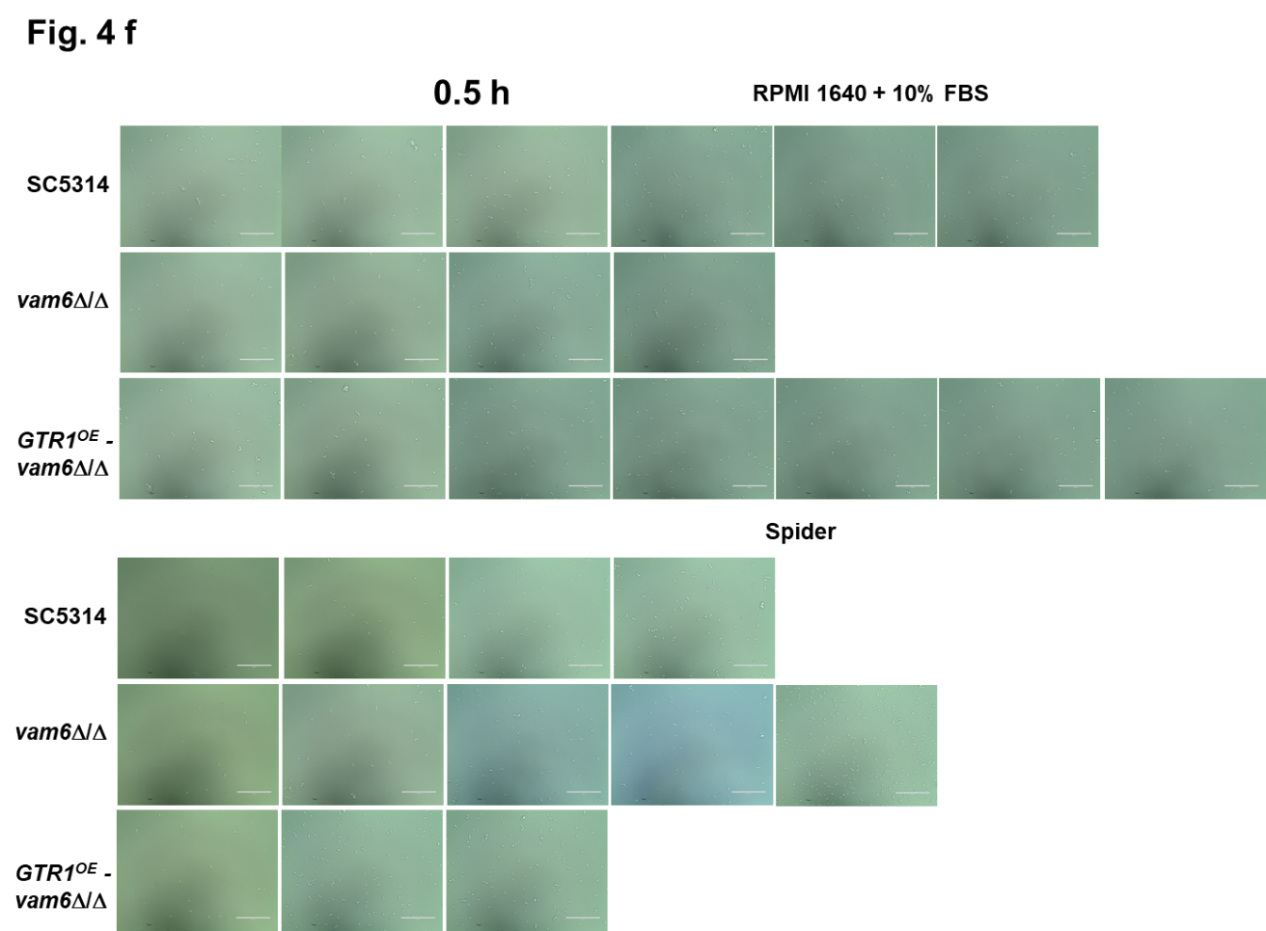
Fig. 4f. Images used to calculate the ratio of germination.

Fig. 4g. Images used to measure the length of hyphae.


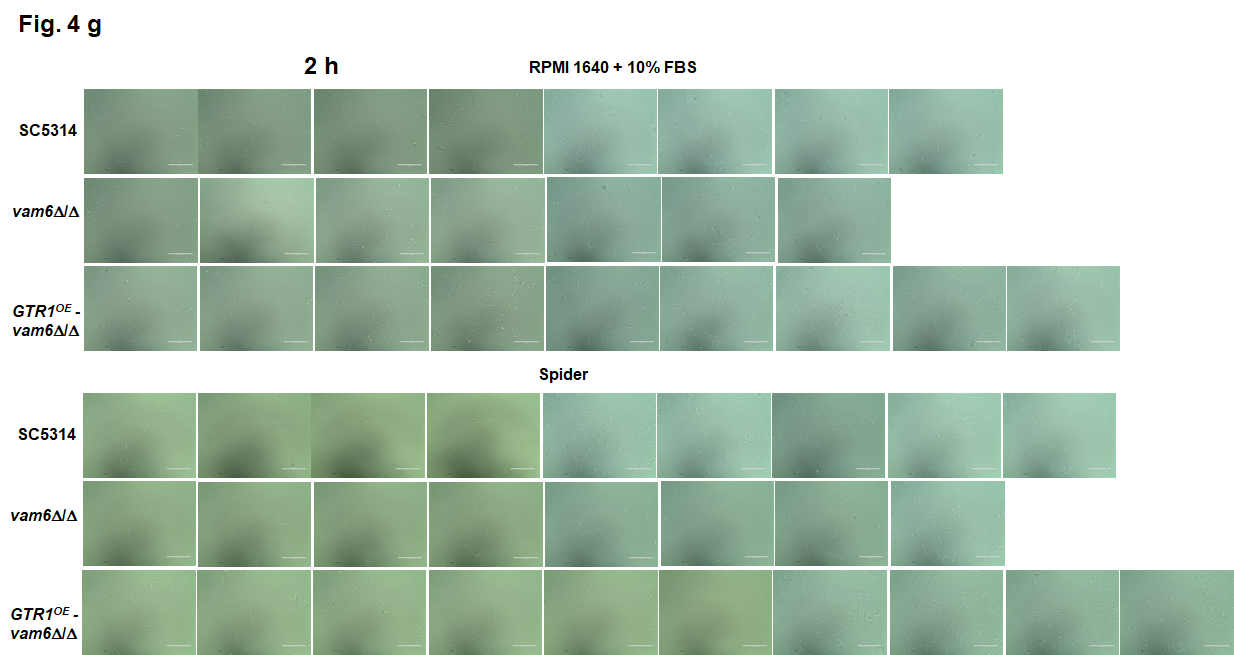


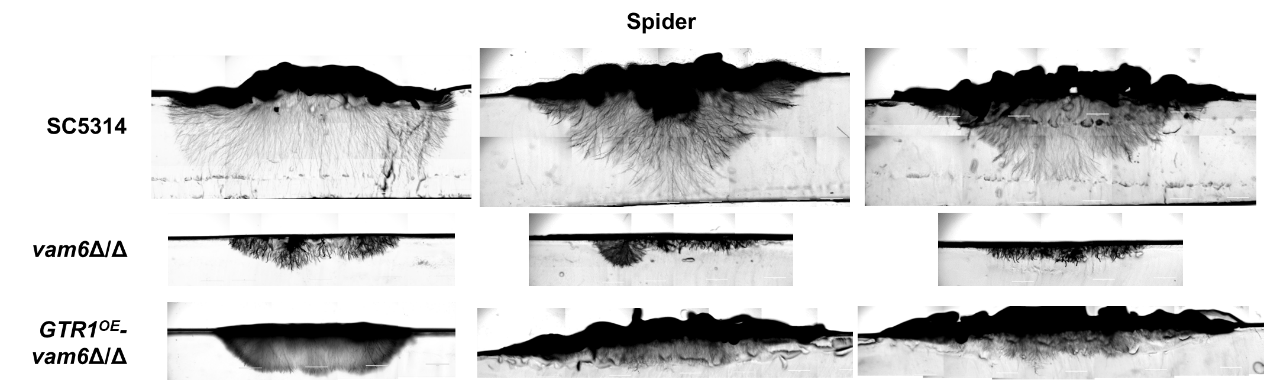

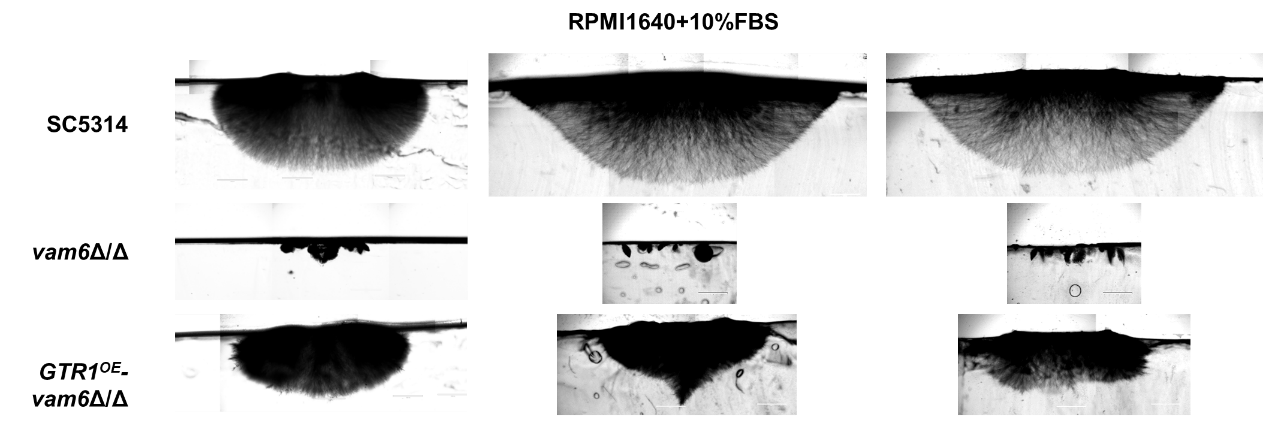
Fig. 4h. Images used to measure the width (W) and depth (D) of hyphal colonies on solid media. Three biological replicates of the vertical sections were observed by microscopy.
