# Supplementary material for: The vacuolar fusion regulated by HOPS complex promotes hyphal initiation and penetration in Candida albicans
Source: Nat Commun. 2024 May 16;15:4131. doi: 10.1038/s41467-024-48525-5 (PMC11099166; doi:10.1038/s41467-024-48525-5)

**Source data of Figure 5.**


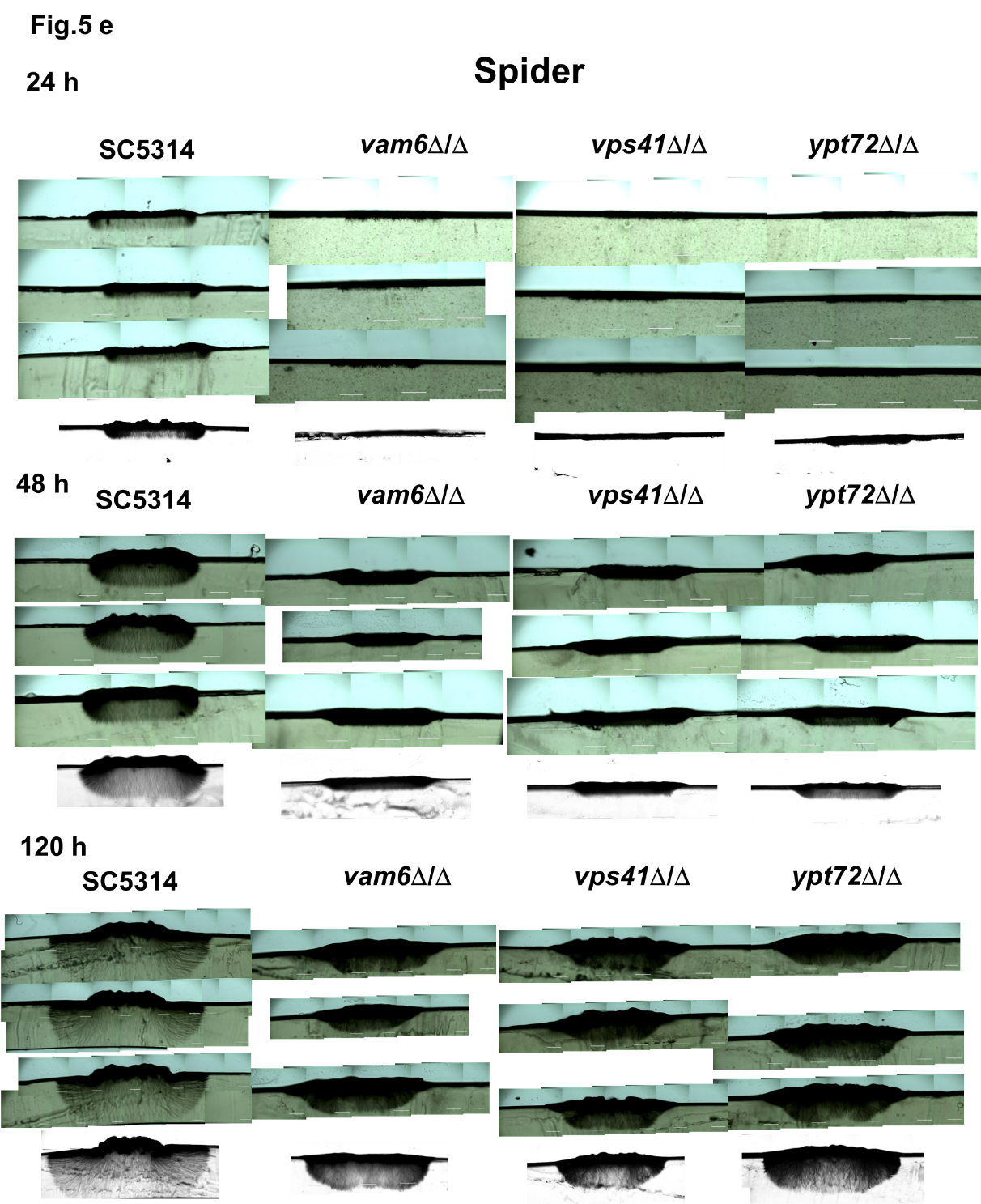
Fig. 5e and 5f. Images used to measure the width (W) and depth (D) of hyphal colonies on solid media.


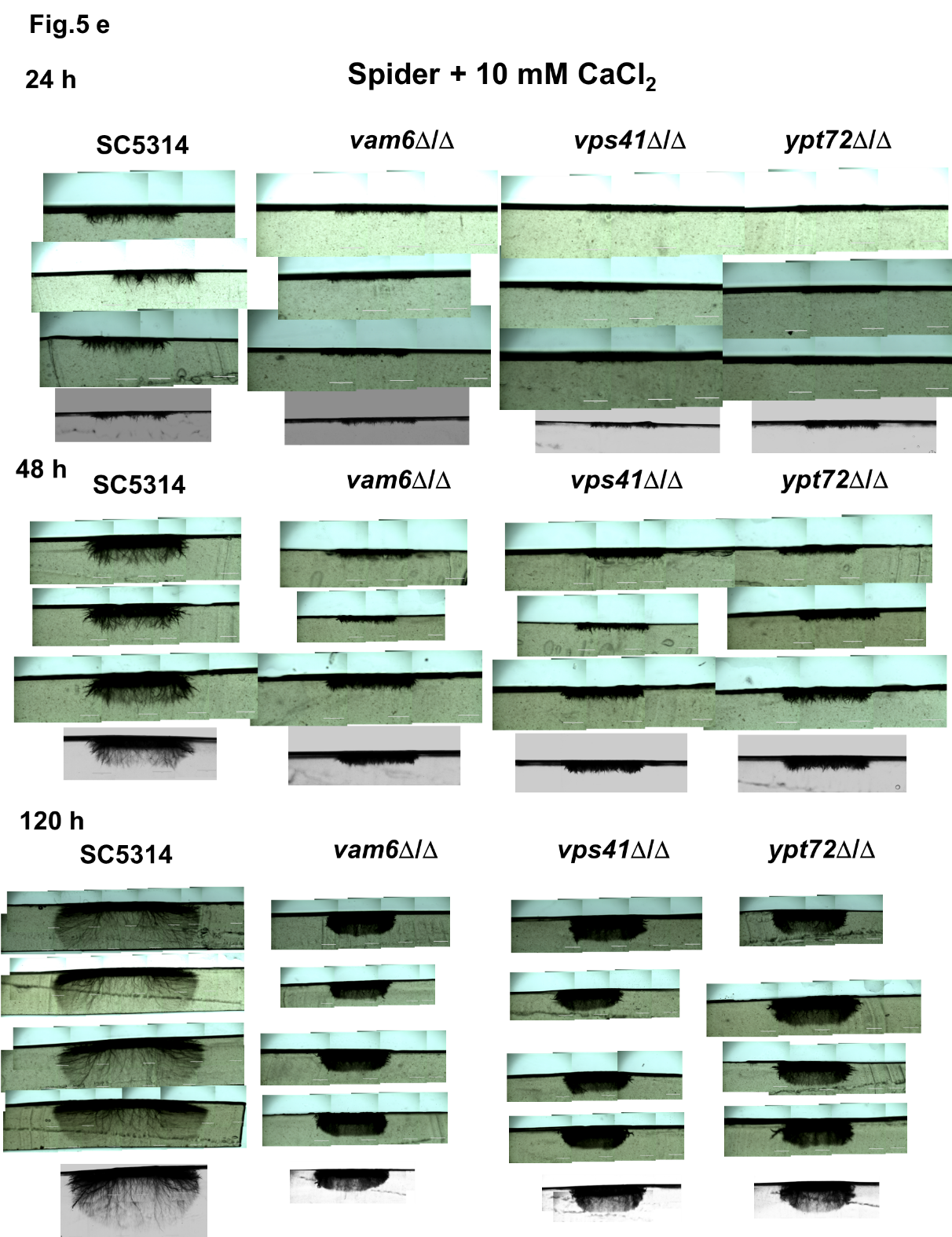

Supplement: Supplementary file 12 — Source Data [file 41467_2024_48525_MOESM12_ESM.zip › Source Data of Figure 5.docx]
