# Supplementary material for: The vacuolar fusion regulated by HOPS complex promotes hyphal initiation and penetration in Candida albicans
Source: Nat Commun. 2024 May 16;15:4131. doi: 10.1038/s41467-024-48525-5 (PMC11099166; doi:10.1038/s41467-024-48525-5)
Supplement: Supplementary file 12 — Source Data [file 41467_2024_48525_MOESM12_ESM.zip › Source Data of Figure 7.docx]

**Source data of Figure 7.**


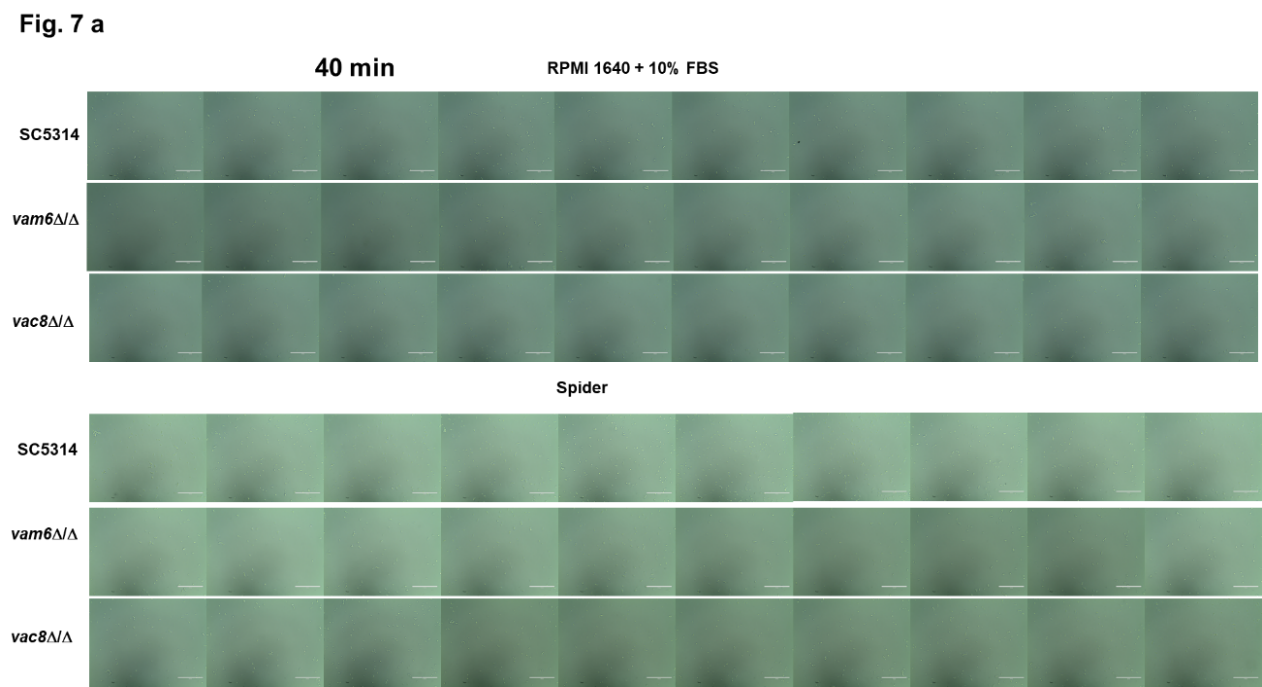
Fig. 7a. Images used to calculate the ratio of germination.


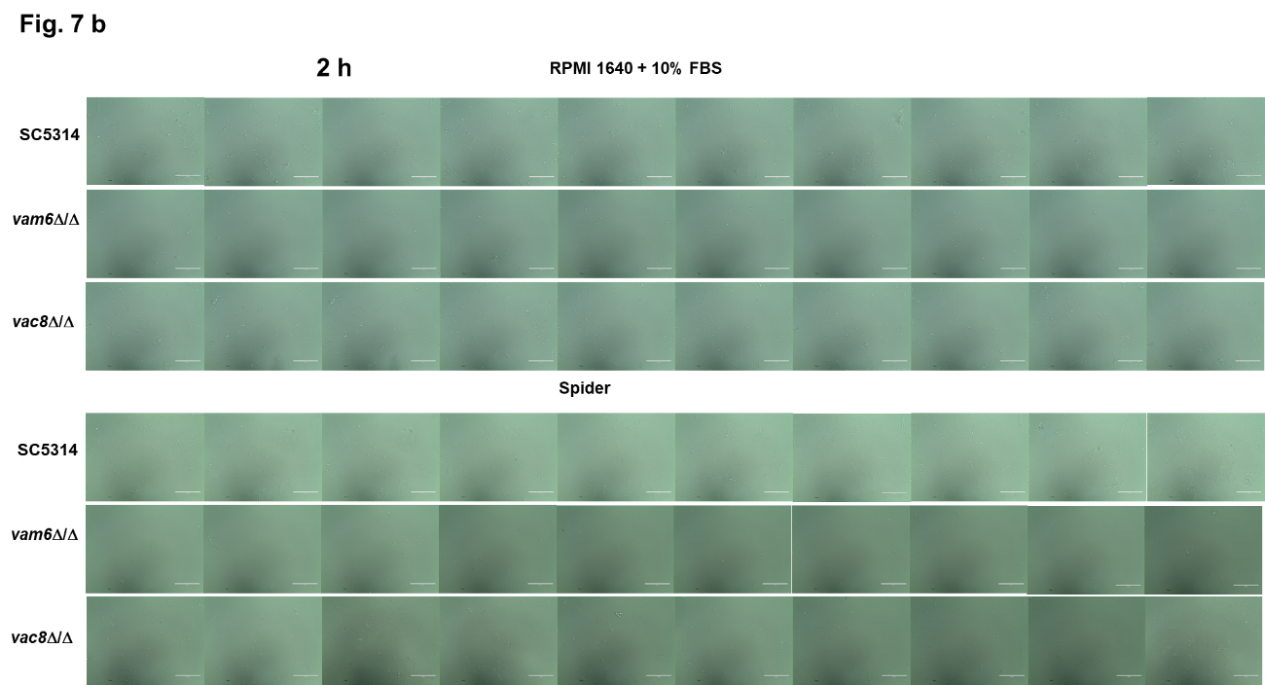
Fig. 7b and 7c. Images used to measure the length of hyphae.


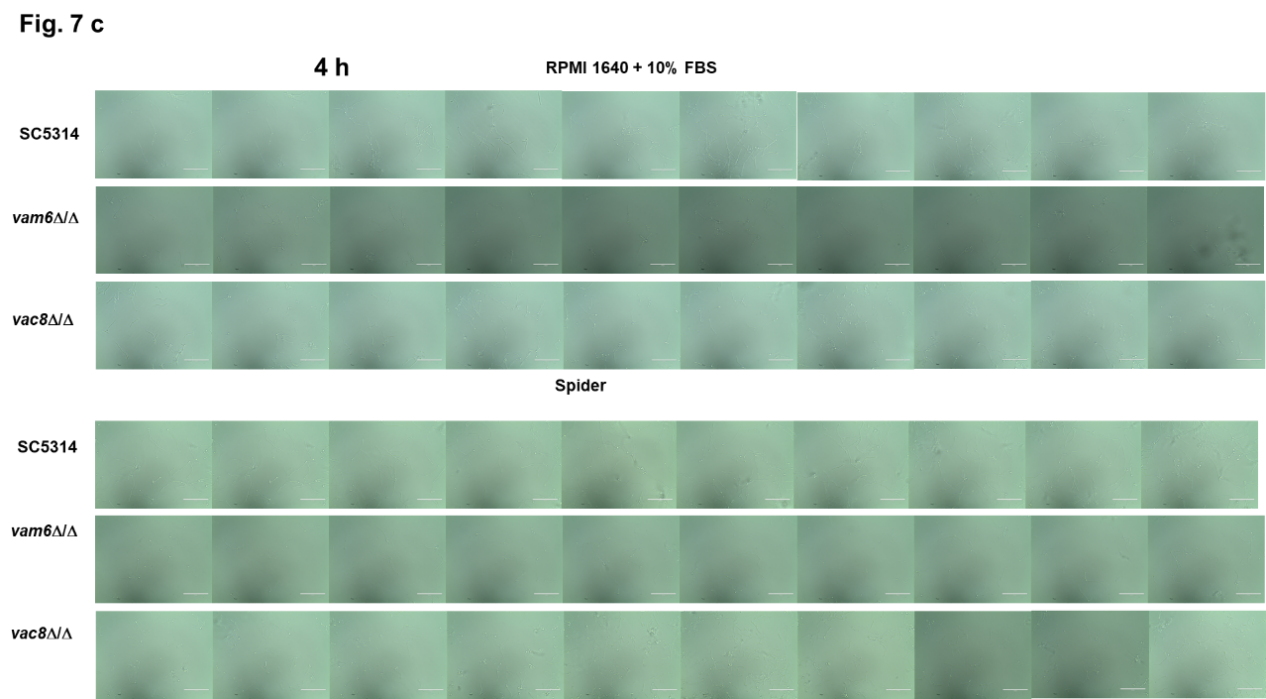


Fig. 7d and 7e. Images used to measure the width (W) and depth (D) of hyphal colonies on solid media.


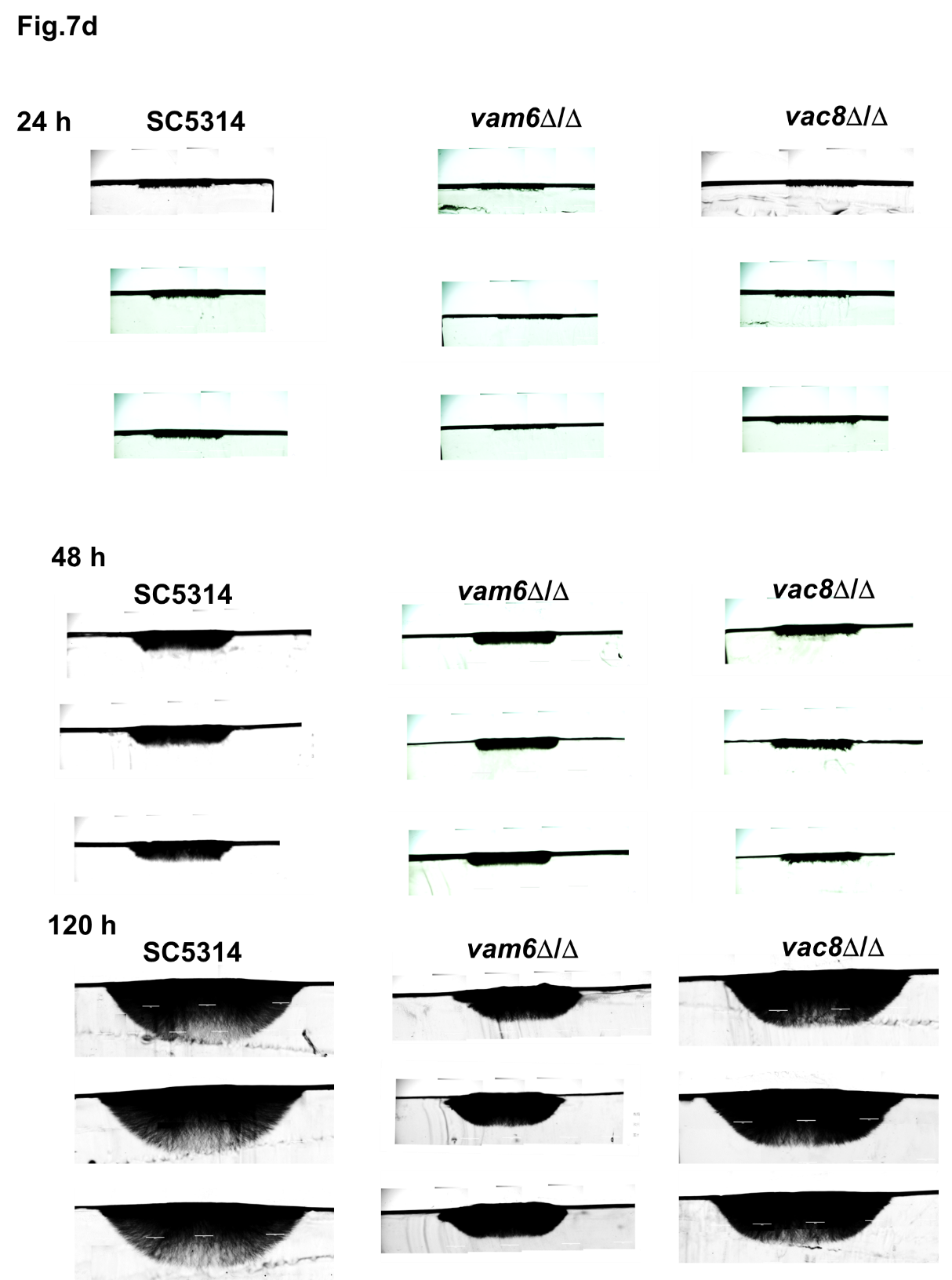

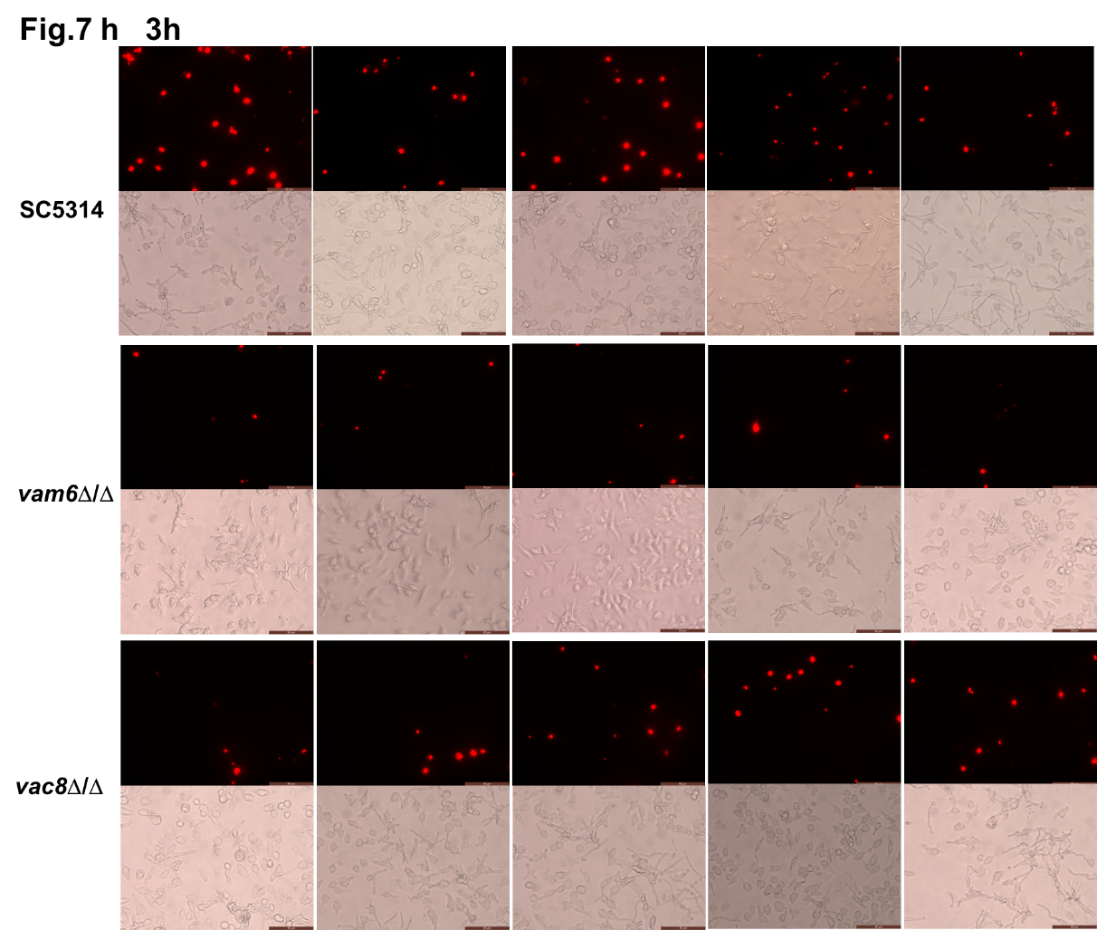

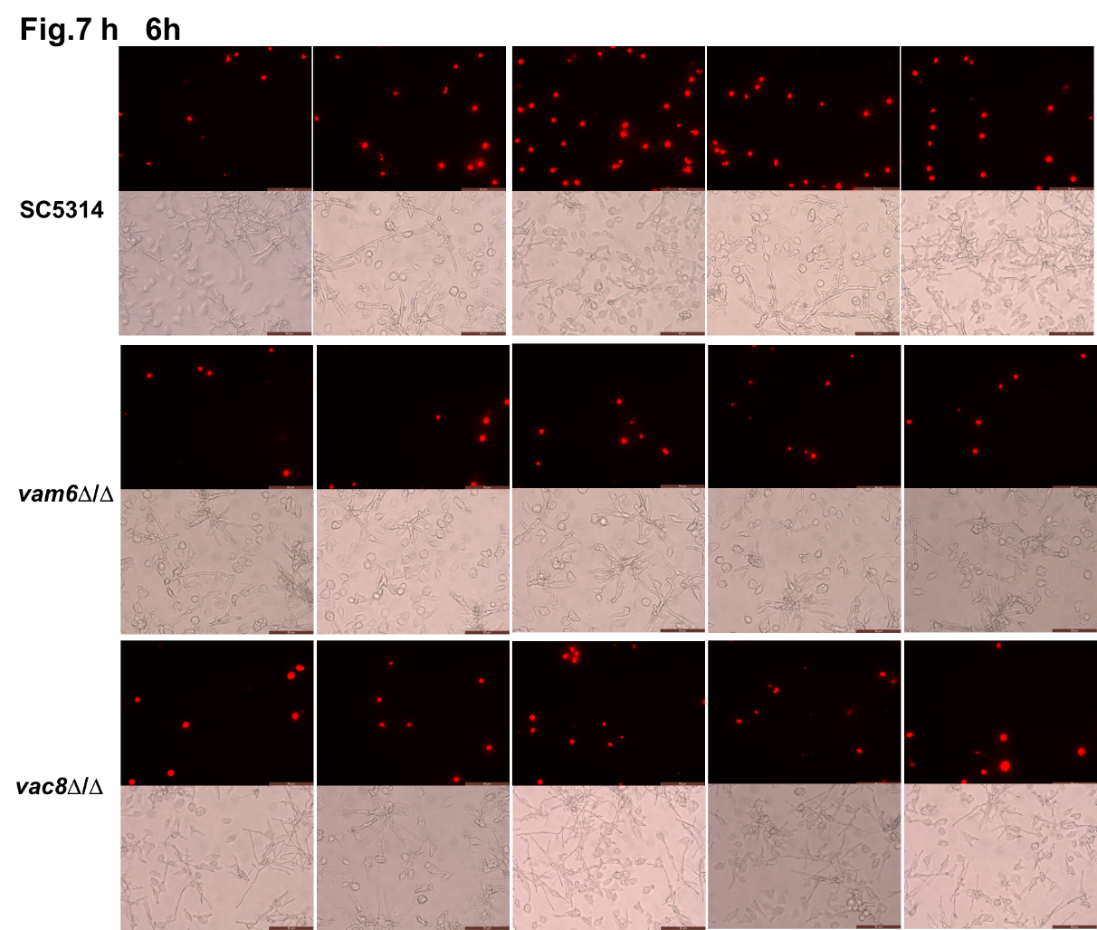
Fig. 7h-j. Images used to calculate the percentage of PI-positive macrophages co-incubated with *C. albicans*.


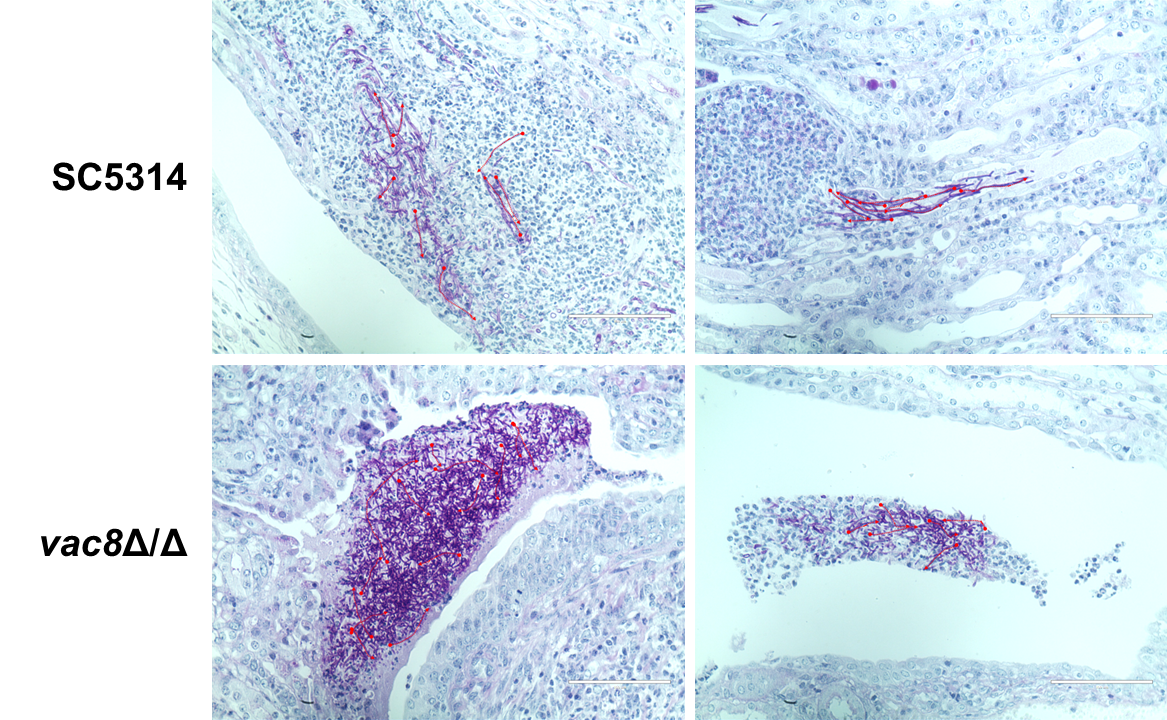
Fig. 7n. Images used to measure the length of hyphae in kidneys in mice infected with *C. albicans*. Some of the measured hyphae have been shown in red lines.


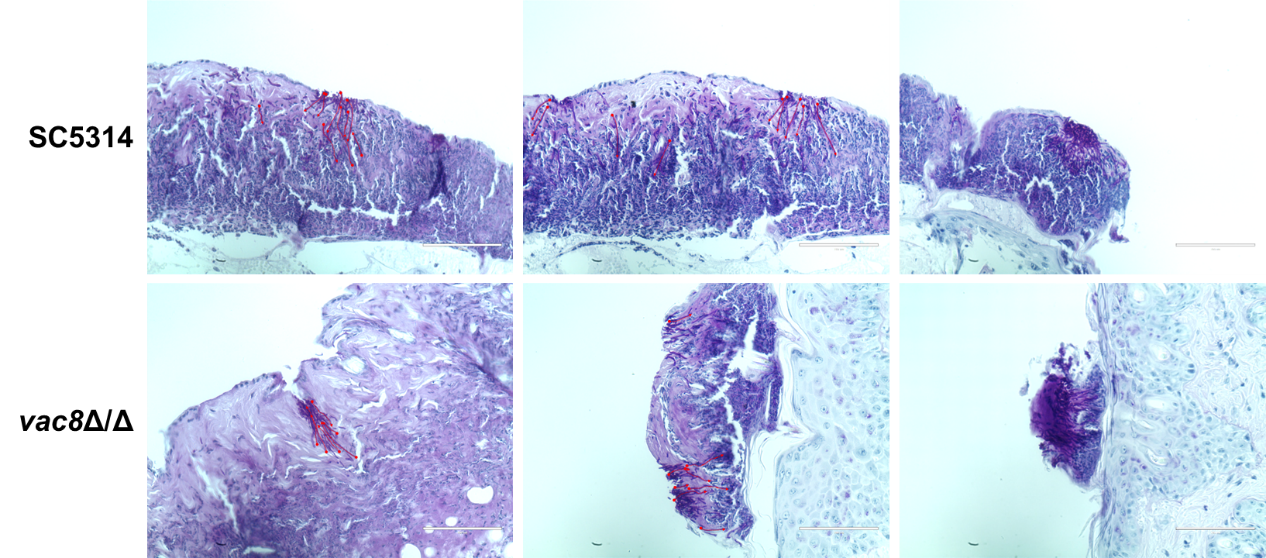
Fig. 7r. Images used to measure the length of hyphae in skin in mice infected with *C. albicans*. Some of the measured hyphae have been shown in red lines.
