# Supplementary material for: The vacuolar fusion regulated by HOPS complex promotes hyphal initiation and penetration in Candida albicans
Source: Nat Commun. 2024 May 16;15:4131. doi: 10.1038/s41467-024-48525-5 (PMC11099166; doi:10.1038/s41467-024-48525-5)

**Source data of Supplementary Figures.**


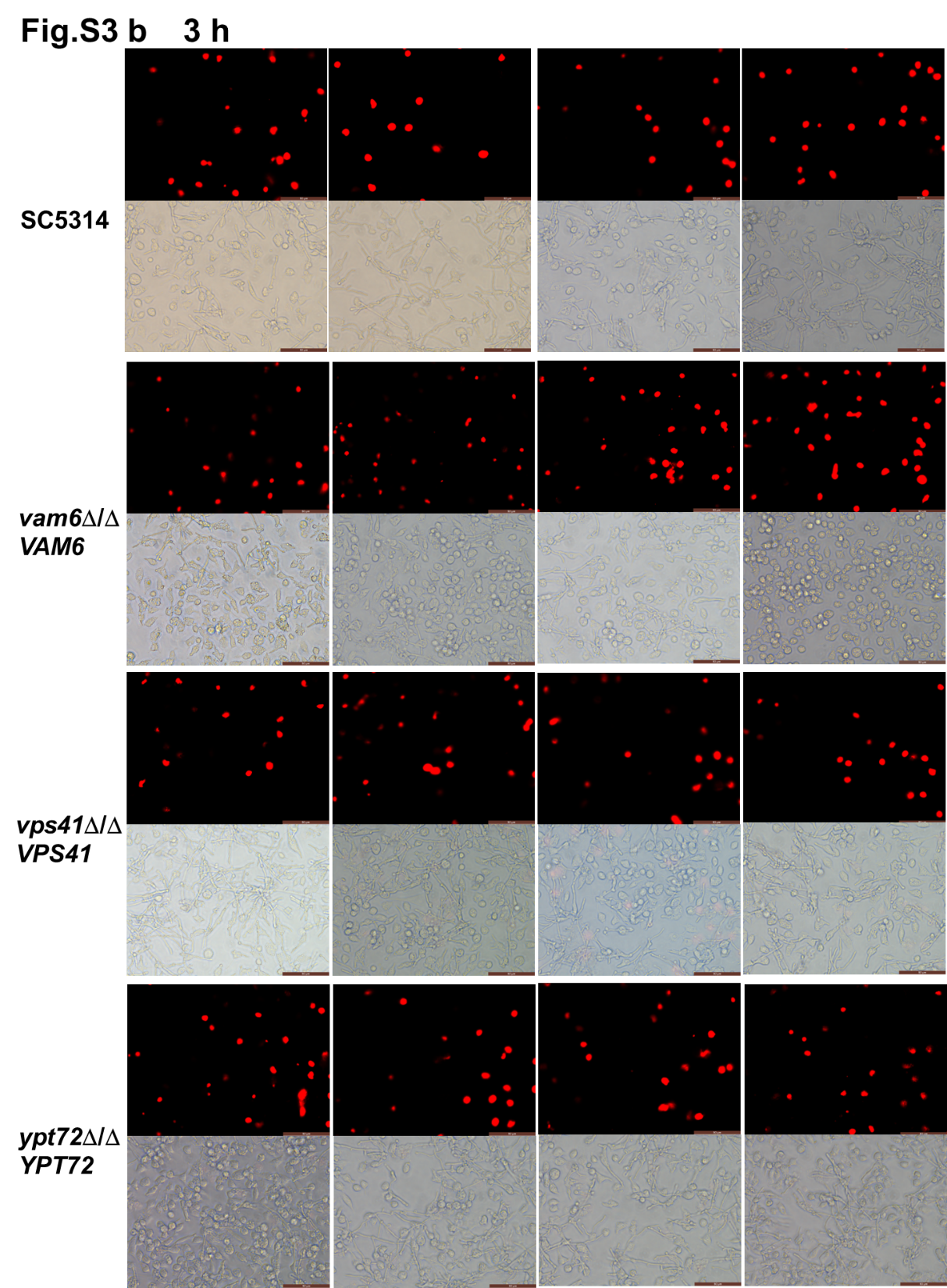
Fig. S3b. Images used to calculate the percentage of PI-positive macrophages co-incubated with *C. albicans*.


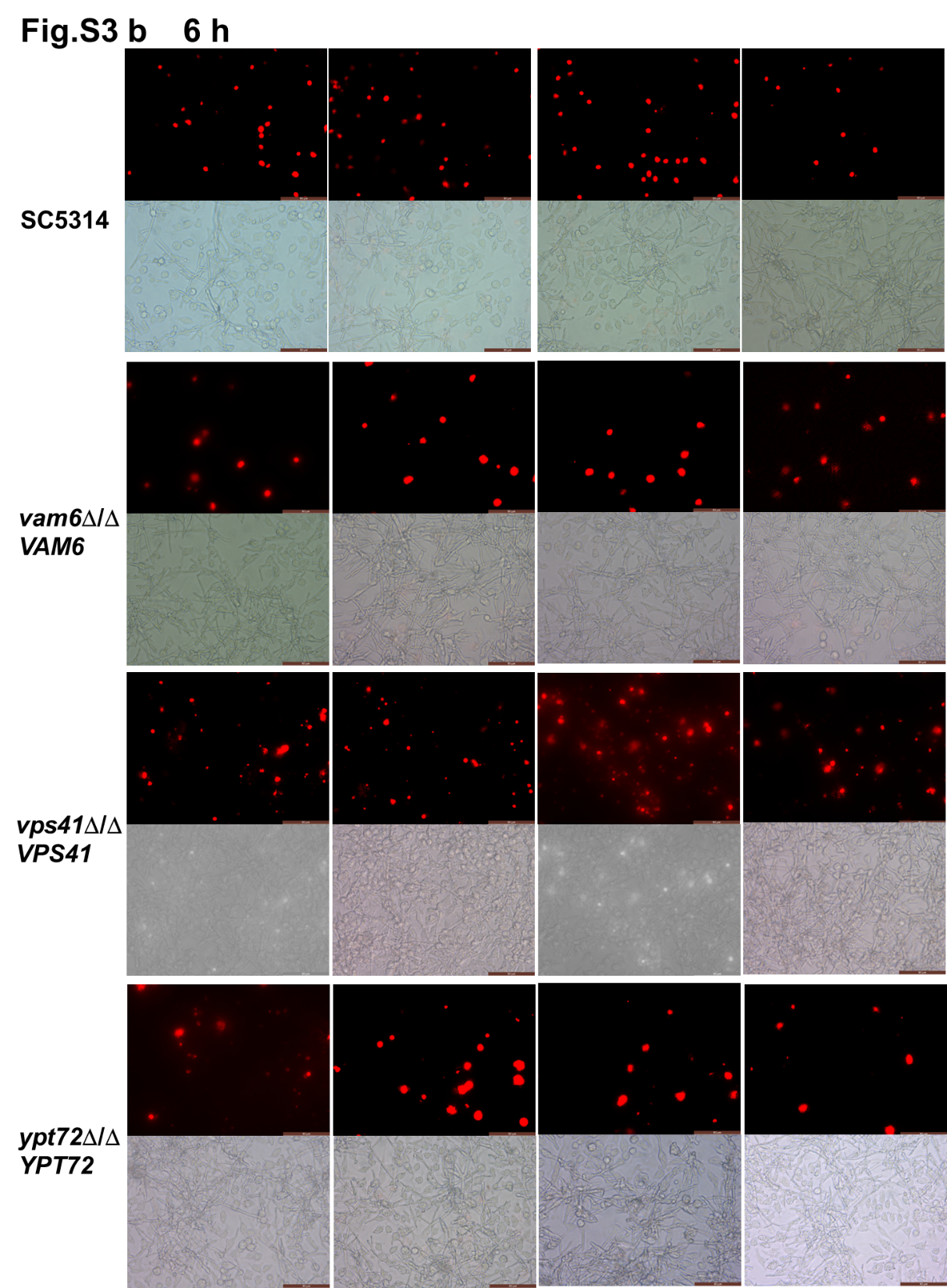


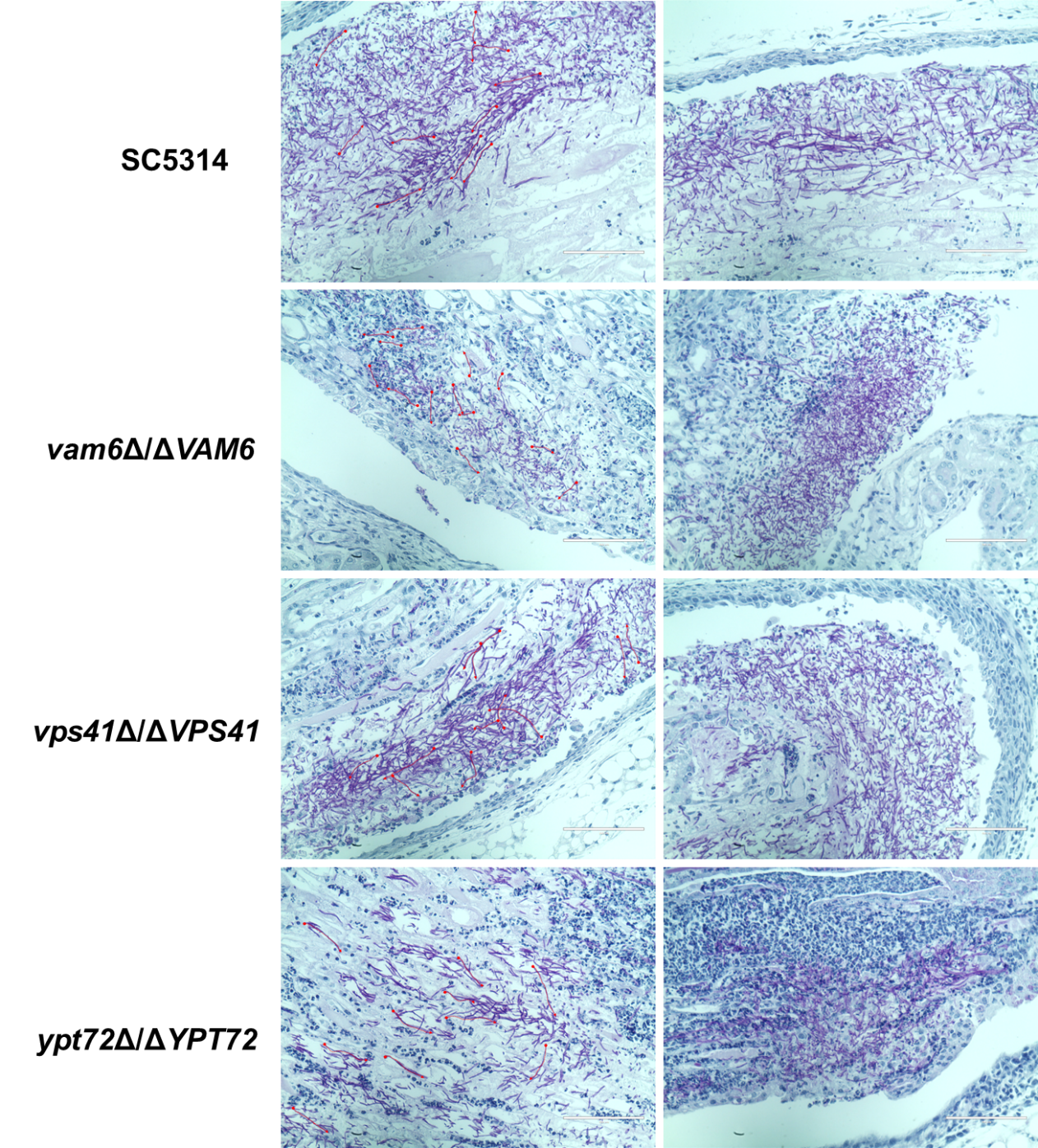
Fig. S3e. Images used to measure the length of hyphae in kidneys in mice infected with *C. albicans*. Some of the measured hyphae have been shown in red lines.


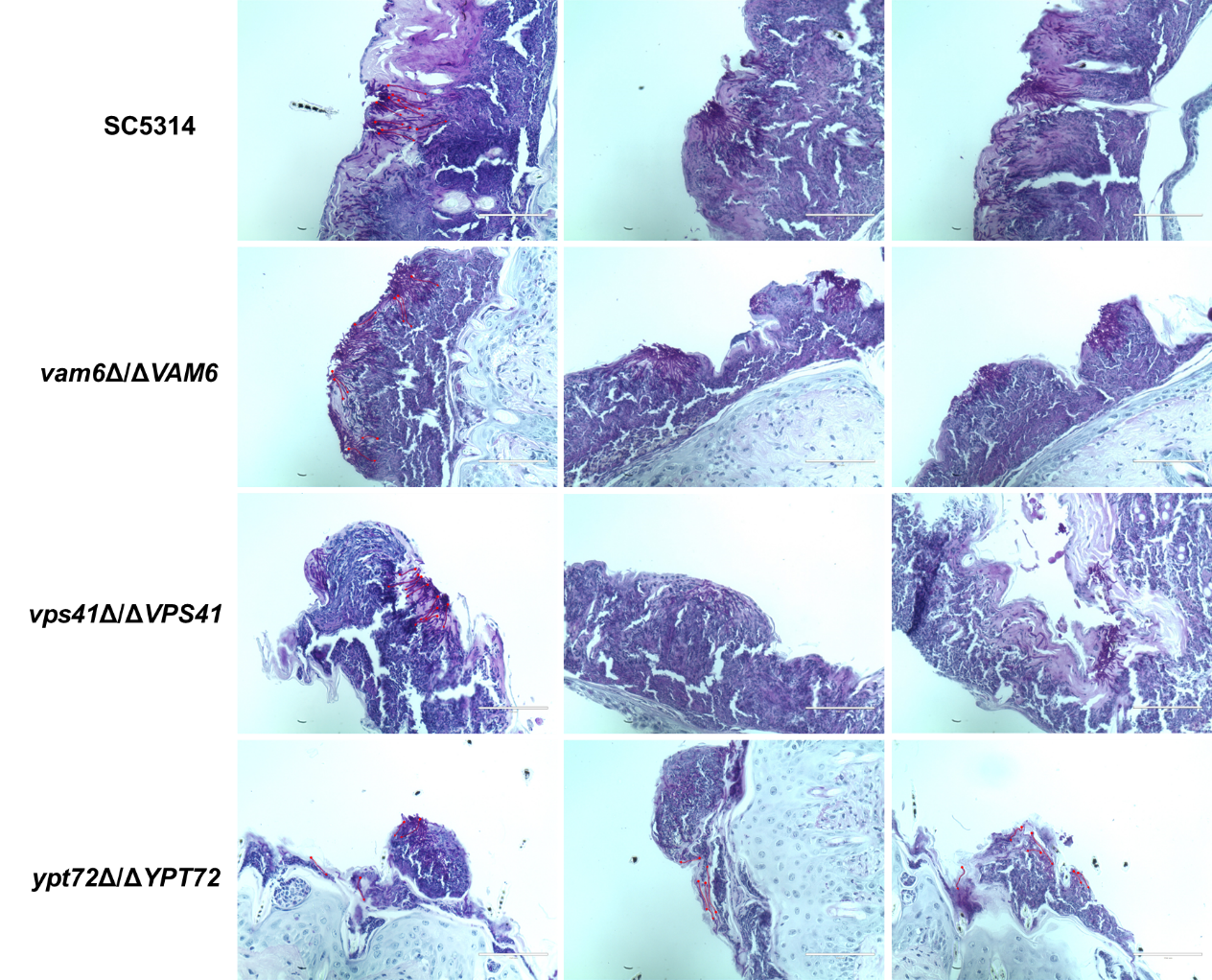
Fig. S3i. Images used to measure the length of hyphae in skin in mice infected with *C. albicans*. Some of the measured hyphae have been shown in red lines.


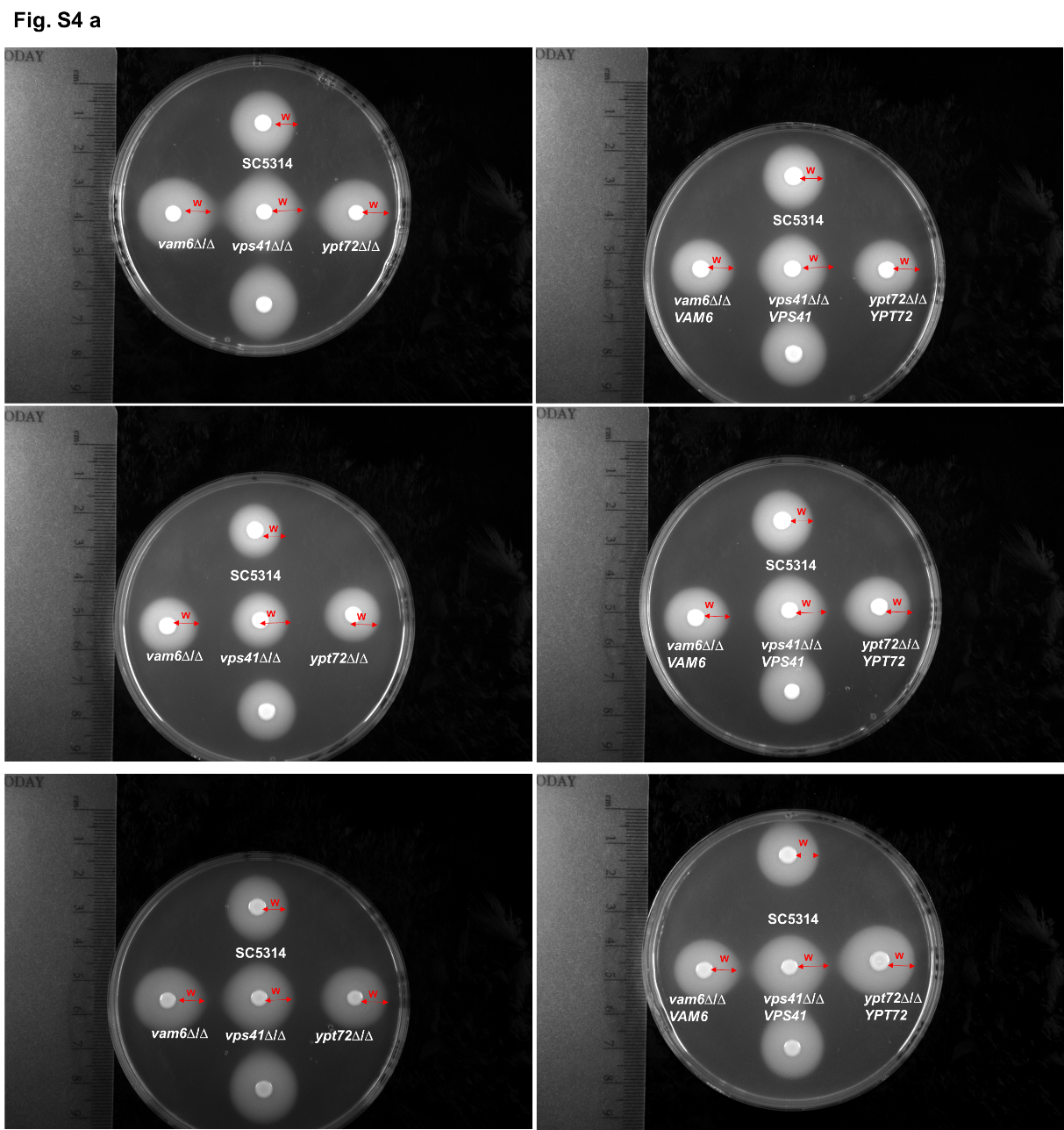
Fig. S4a. Images used to measure the sizes of halo around the colonies.

**
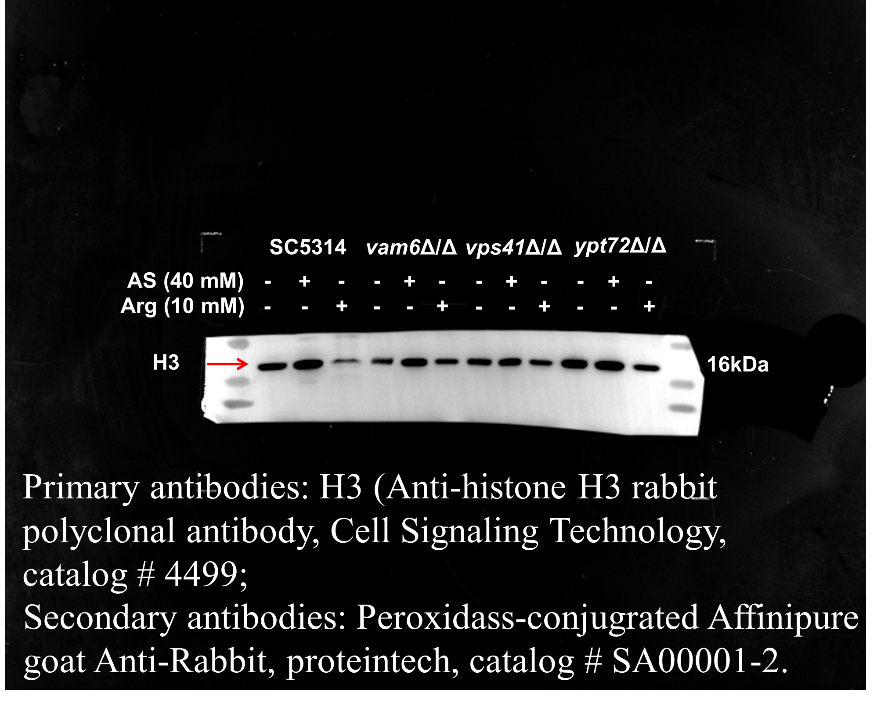
**
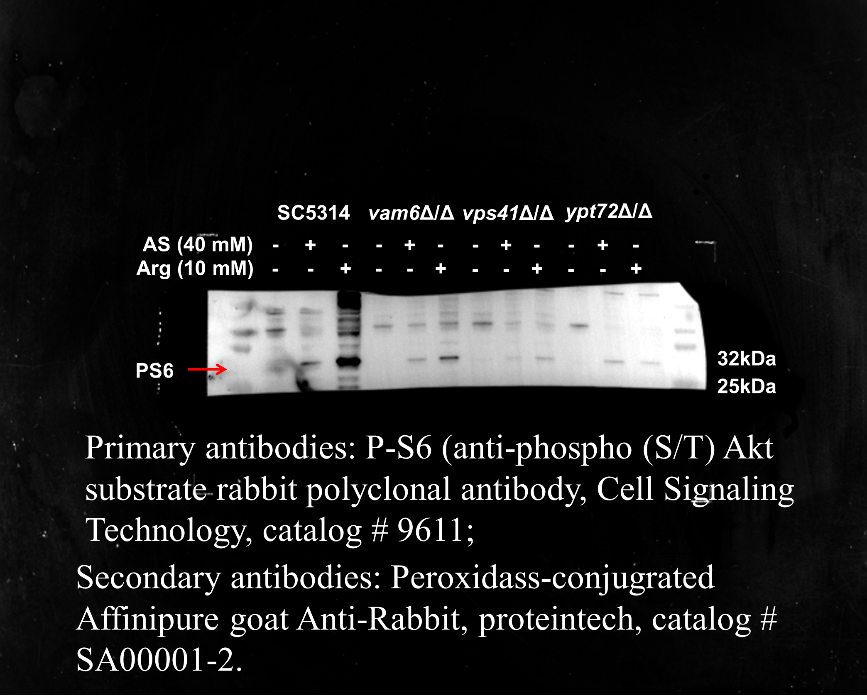
Fig. S4c. Original images of Western blots.


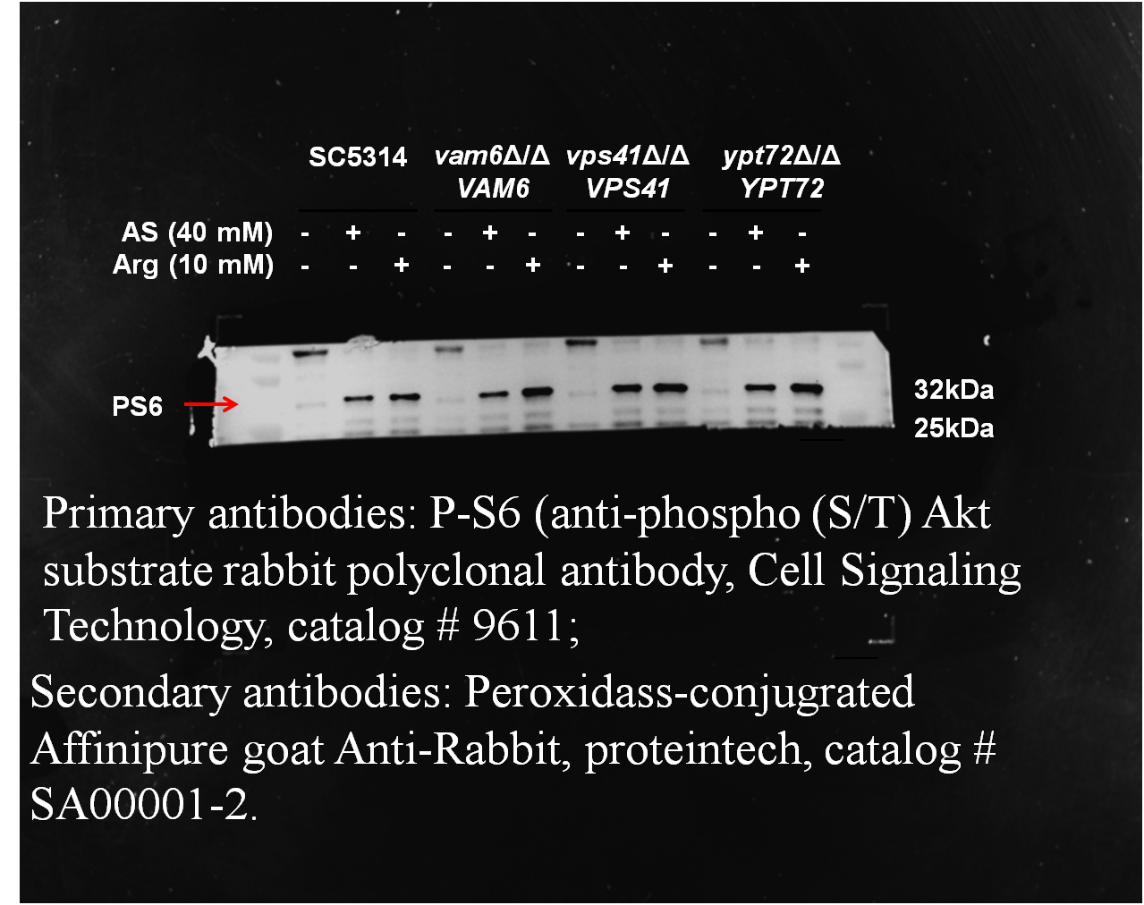

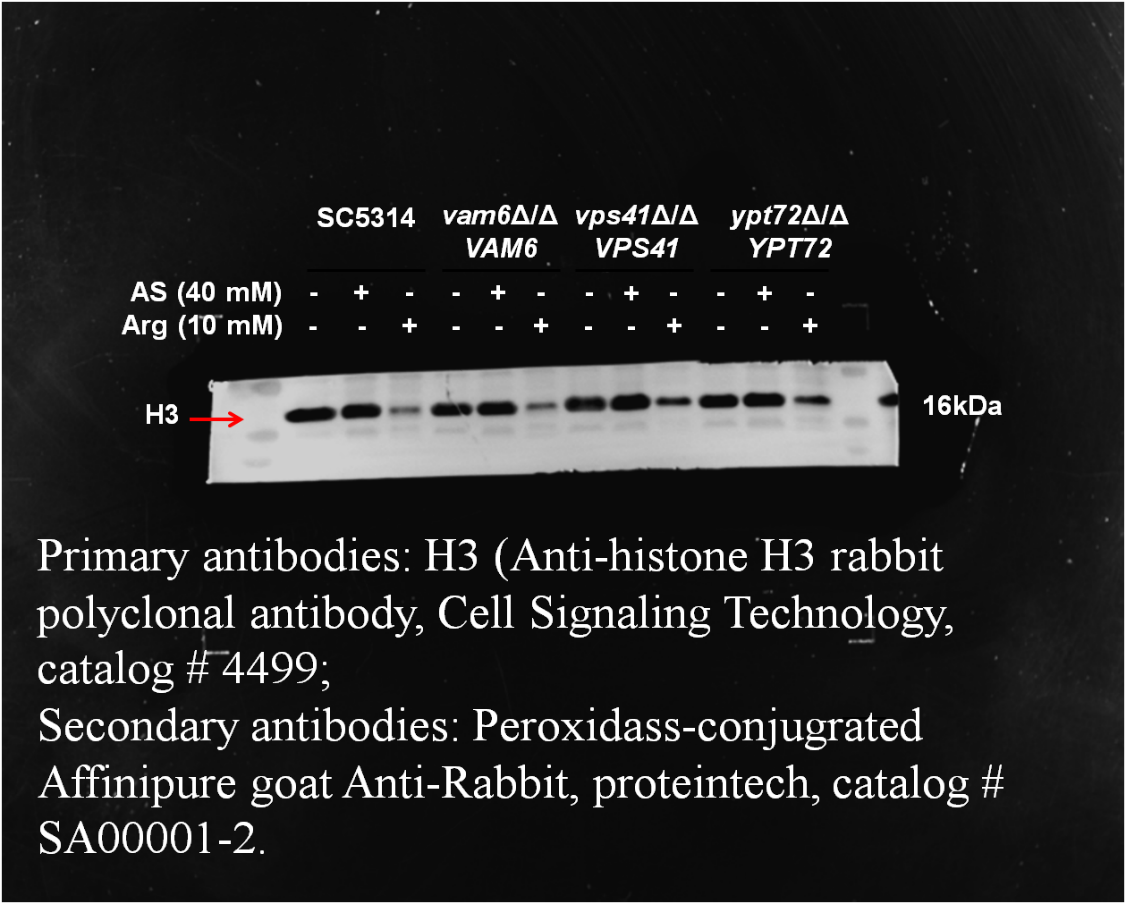
Fig. S4d. Original images of Western blots.


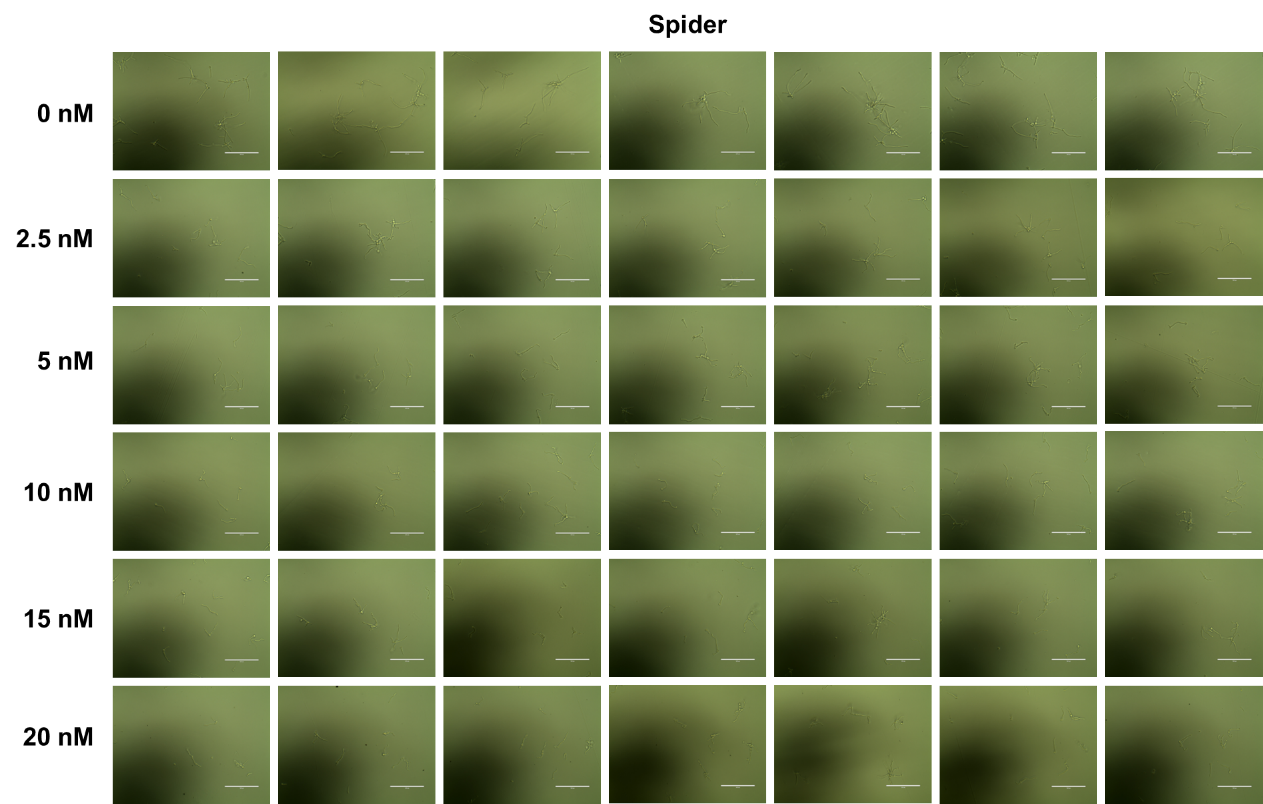
Fig. S4g. Images used to measure the length of hyphae.


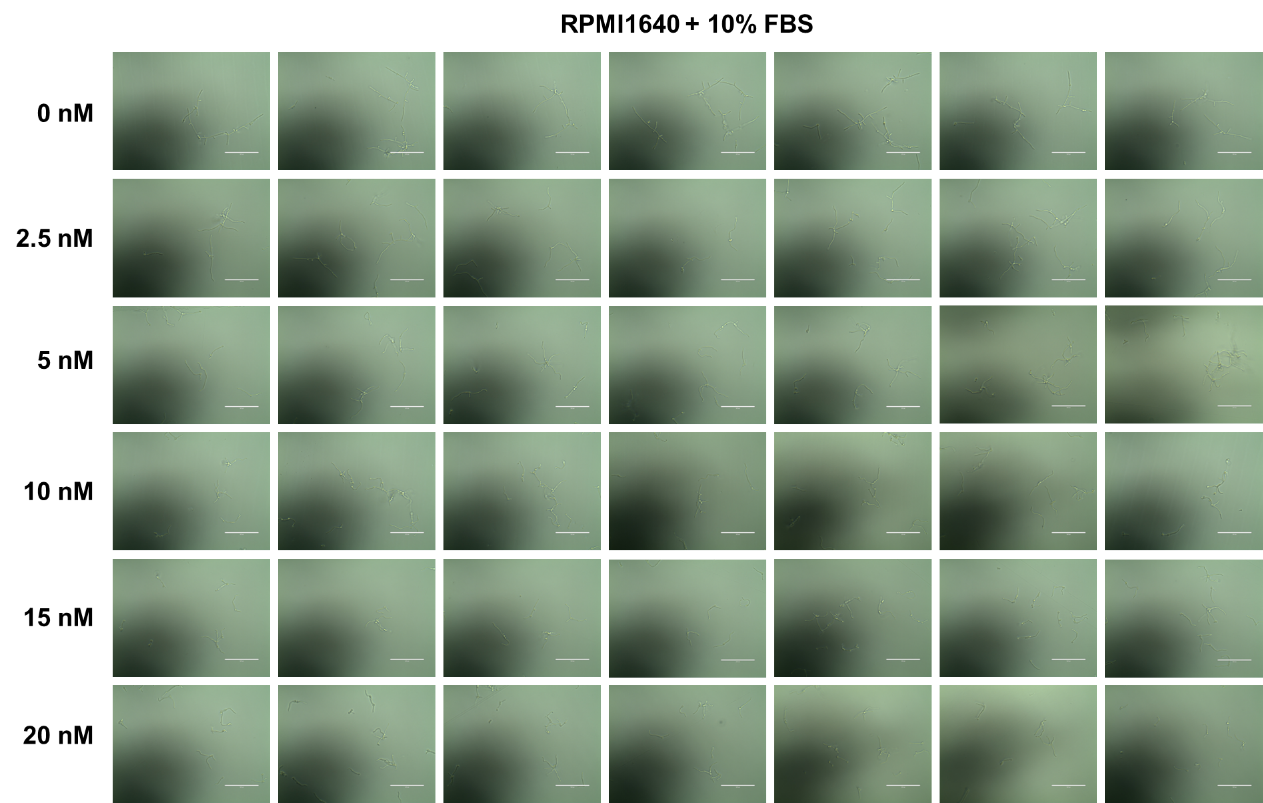

Supplement: Supplementary file 12 — Source Data [file 41467_2024_48525_MOESM12_ESM.zip › Source Data of Supplementary Figures.docx]
